# Staple oligomers induce a stable RNA G-quadruplex structure for protein translation inhibition in therapeutics

**DOI:** 10.1038/s41551-025-01515-4

**Published:** 2025-10-15

**Authors:** Yousuke Katsuda, Takuto Kamura, Tomoki Kida, Rinka Ohno, Shuhei Shiroto, Yua Hasegawa, Kaito Utsumi, Yuki Sakamoto, Shinichiro Nakamura, Taishi Nakamura, Kenichi Tsujita, Yusuke Kitamura, Yukiko Kamiya, Hiroyuki Asanuma, Toshihiro Ihara, Masaki Hagihara, Shin-ichi Sato

**Affiliations:** 1 Division of Materials Science and Chemistry, Faculty of Advanced Science and Technology, Kumamoto University, Kumamoto, Japan; 2 StapleBio Inc., Kumamoto, Japan; 3 Graduate School of Science and Technology, Hirosaki University, Aomori, Japan; 4 Faculty of Medicine, Osaka University, Osaka, Japan; 5 Priority Organization for Innovation and Excellence Laboratory for Data Science, Kumamoto University, Kumamoto, Japan; 6 Department of Medical Information Science, Graduate School of Medical Sciences, Kumamoto University, Kumamoto, Japan; 7 Department of Cardiovascular Medicine, Graduate School of Medical Sciences, Kumamoto University, Kumamoto, Japan; 8 Department of Biomolecular Engineering, Graduate School of Engineering, Nagoya University, Nagoya, Japan; 9 Laboratory of Bioanalytical Chemistry, Kobe Pharmaceutical University, Kobe, Japan; 10 Institute for Chemical Research, Kyoto University, Kyoto, Japan

**Keywords:** Gene therapy, RNA

## Abstract

Gene-silencing mechanisms using RNA interference (RNAi) and antisense oligomers have drawn attention to nucleic acid medicines. However, several challenges remain such as low in vivo stability and inadequate target selectivity. Here we report a versatile and highly selective method for suppressing gene expression with a short oligonucleotide. The oligonucleotide, a Staple oligomer, hybridizes specifically to a target mRNA and artificially induces a higher-order structure, an RNA G-quadruplex (rG4), on the mRNA. This results in the rG4 effectively suppressing the target protein’s translation. The method is validated by successfully regulating translation of TPM3, MYD88 and TRPC6 mRNAs in a cell-free system and in living mammalian cells. Unlike RNAi and antisense technologies, the present technology does not require cooperation with bioprocesses, permitting the quick and easy development of fully non-natural nucleic acid-based Staple oligomers without compromising their effectiveness. In vivo application of the technology to TRPC6 mRNA helps prevent cardiac hypertrophy in thoracic aortic constriction-treated mice without detectable off-target effects. This technology provides new insights into gene therapy after RNAi and antisense technologies.

## Abstract (web summary)

A nucleic acid-based therapeutic uses short Staple oligomers to hybridize with target mRNAs and induce an RNA G-quadruplex, suppressing protein translation.

## Main

Developing small and medium-sized molecular compounds that act as orphan drugs or therapeutic agents for rare diseases remains challenging. Gene therapy is a promising medical approach that now allows more precise and personalized treatment of serious and rare diseases since gene therapeutics can sequence-specifically target a disease-causing gene. Nucleic acids can be used for drugs as seed molecules based on the sequence of the target gene. Nucleic acid drugs can target DNA and RNA, unlike most conventional low-molecular-weight compounds. For example, RNA interference (RNAi) is used to suppress target gene expression in a sequence-specific manner through mRNA degradation or translational inhibition^1^. In 2008, Patisiran was approved as the first commercial RNAi-based therapeutic by the US Food and Drug Administration and the European Medicines Agency for the treatment of hereditary amyloidogenic transthyretin amyloidosis with polyneuropathy in adults^2^. Nusinersen, an antisense oligonucleotide whose mechanism of action is exon inclusion, was recently developed and successfully demonstrated to have a substantial therapeutic effect on spinal muscular atrophy^3^, for which no treatment has yet been established.

Despite the advantages, the development of nucleic acid drugs that suppress gene expression remains challenging. While small interfering RNAs are the first choice in in vitro studies for suppressing the expression of target genes, few nucleic acid drugs use the RNAi mechanism, partially due to their off-target effects^4–6^, low stability in vivo^7^, drug delivery^8,9^ and induction of innate immunity^10^. There is a huge gap between experimental and pharmaceutical technologies in use, making practical application difficult. Various attempts have been made to bridge this gap. For example, computational chemistry-based software is being developed that uses basic thermodynamic stability information to avoid off-target effects^11–15^. Meanwhile, since genetic information contains many sequences similar to the target gene, methods based on computational chemistry may be ineffective in the presence of homologous target genes. To prevent in vivo degradation, artificial nucleic acids with non-natural structures are introduced to nucleic acid drugs, such as BNA (bridged nucleic acid)^16,17^, GNA (glycol nucleic acid)^18–20^, TNA (threose nucleic acid)^21,22^, morpholino^23,24^ and other nucleic acid analogues with various substitutions in the ribose ring^25,26^. Although such non-natural nucleic acids remarkably stabilize nucleic acid drugs, nucleic acid-based therapeutics still pose a challenge because their intrinsic ability to suppress gene expression is greatly impaired by their structures being incompatible for critical bioprocessing, including enzymatic reactions by Argonaute or RNaseH. More specifically, seed molecules for nucleic acid medicine can be developed in a short period of time, but the cost required for optimization is not much different from the cost of developing conventional small-molecule medicines. Thus, developing a technology that compensates for the shortcomings of conventional nucleic acid drugs would be a promising medical approach, allowing more precise and personalized treatment of serious and rare diseases. Here, we report a versatile and highly selective technology to modify mRNA using short nucleic acids, named Staple oligomers, by inducing a stable RNA G-quadruplex (rG4) structure that inhibits protein translation. We call this RNA hacking (RNAh).

**Fig. 1:**
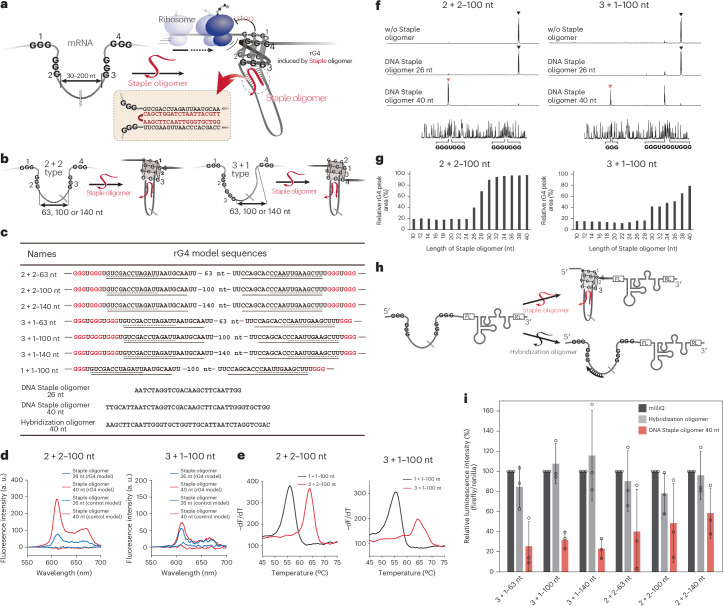
Characterizing RNAh technology. **a**, Mechanism of action of RNAh. rG4 induced by RNAh technology inhibits ribosome scanning, resulting in translational suppression. **b**, rG4 models have four G-tracts that are split into a 2 + 2 or 3 + 1 arrangement by 63-, 100- or 140-nt-long loops. **c**, Nucleotide sequences of rG4 models, Staple oligomer and Hybridization oligomer. G-tracts are shown in red. The dashed and solid underlines represent the 26-nt or 40-nt Staple oligomer recognition sites. **d**, Evaluation of rG4 formation with NMM. The blue and red curves show the fluorescence emission spectra of NMM in the presence of 26-nt or 40-nt DNA Staple oligomers, respectively. The solid and dashed curves represent the model sequence with G-tracts and the control sequence with A-tracts, respectively. **e**, Thermal denaturation profiles of Staple oligomer hybrids. An RNA strand containing either the 2 + 2–100-nt or the 1 + 1–100-nt sequence was complexed with a 5′-end FAM-labelled Staple strand, and the fluorescence intensity of the samples was measured from 20 °C to 100 °C. The graph displays the negative first derivative of the melting curve data (−*dF*/*dT*) versus temperature. Thermal melting curves of 2 + 2–100-nt and 1 + 1–100-nt sequences are shown in black and red, respectively. **f**, Identification of sequencing spectra on 2 + 2–100-nt and 3 + 1–100-nt rG4 model sequences by the RTase stop assay. The red arrowheads indicate the arrest site of RTase with rG4 induced by the Staple oligomers,and black arrowheads indicate the RTase elongation end without (w/o) rG4 induction. To indicate the location of the stop products, all sequencing spectra are represented below each specific sequencing spectrum. **g**, Evaluation of the effect of nucleotide length of DNA Staple oligomers on rG4 induction by the RTase stop assay. **h**, Dual reporter genes encoding FL and RL in tandem were used to validate the mechanism of action of RNAh. rG4 model sequence was placed in the 5′ UTR of FL. RL was placed downstream of an IRES as an internal standard. Staple oligomers induce rG4 formation (top), whereas hybridization oligomers do not (bottom). **i**, The effect of a 40-nt DNA Staple oligomer on the translation of reporter RNA in vitro. Data are mean ± s.d. of three independent experiments.

rG4 is a thermodynamically stable nucleic acid structure that inhibits ribosome scanning in the 5′ untranslated region (UTR) of mRNA and suppresses protein translation^27^. It has also been reported that rG4 of FMR1 mRNA acts as a regulatory element for its alternative splicing^28^. To form rG4, a minimal requirement is four consecutive guanine triplets (G_3_; G-tracts). Huppert et al. reported that an rG4 is formed in the genes whose sequences conform to the (G_3_–N_1–__7_–G_3_–N_1–__7_–G_3_–N_1–__7_–G_3_) rule^29^. Various other subtypes of rG4 formation that is not rule bound have also been identified, including bulge rG4, long-loop rG4 and 2 G-quartet rG4 (ref. ^30^). Among them, it was recently reported that genes with longer loop bases (N_7+_) also form rG4 structures^31,32^. In this case, the loop region may form a higher-order structure and the G-tracts are thought to be in spatial proximity to each other. This led to the idea that, if a short nucleic acid that binds to the flanking sequences of two distant G-tracts is designed to draw the G-tracts close together, specific rG4 should be induced at the targeted locations in long RNA. Since the structures of rG4 are very stable and ribosomes are stalled or arrested at the rG4 site in the 5′ UTR, Staple oligomers could work as suppressors of the target gene (Fig. 1a).

This technology does not rely on the involvement of bioprocesses including enzymatic reactions, as RNAi or antisense technologies. Consequently, it allows the development of less toxic and fully non-natural nucleic acid-based Staple oligomers without compromising their efficacy. In addition, the present technology requires two keys for unlocking the mechanism. First, the simultaneous hybridization of the Staple oligomers to the two target sequences that are far apart from each other and second, the proximity of the split G-tracts to the target sequences, enabling the avoidance of off-target effects, a longstanding problem in the development of nucleic acid drugs. The heightened target specificity inherent in this approach may facilitate its application in targeting highly homologous genes, where computational chemistry alone may fail to circumvent off-target effects.

## Results

### Evaluation of RNAh technology

To evaluate RNAh technology in vitro, we prepared six model sequences with four G-tracts separated by a 63-, 100- and 140-nt intervening loop sequence into a 2 + 2 or 3 + 1 arrangement. Two control sequences with A-tracts instead of G-tracts were also prepared (Fig. 1b,c, Supplementary Fig. 1a and Supplementary Table 1). First, rG4 induction by Staple oligomers was verified by fluorimetry using the rG4 probe thioflavin T (ThT) or *N*-methyl mesoporphyrin IX (NMM), which show enhanced emission at approximately 490 or 610 nm when bound to rG4 (refs. ^33,34^). To confirm the induction of the rG4 structure by Staple oligomers, we designed two DNA Staple oligomers with different lengths (26 or 40 nt). When the designed Staple oligomers were introduced into the rG4 model sequences and the control sequences, remarkable fluorescent signals were successfully observed only in the presence of rG4 model sequences, suggesting that an rG4 structure was induced by the Staple oligomers in all rG4 model sequences (Fig. 1d and Supplementary Figs. 1b and 2). Furthermore, the induction of rG4 by Staple oligomers was validated through measurement of the thermal melting point by monitoring changes in fluorescence associated with the denaturation of target RNA and Staple oligomer complex containing rG4 (Fig. 1e). The topology of rG4 induced by the DNA Staple oligomer was also investigated by circular dichroism (CD) measurements. The CD experiment revealed that the model rG4-forming sequence enhanced the positive Cotton effect at 260 nm and the negative Cotton effect at 240 nm, known as parallel G4 behaviour^35^, in the presence of DNA Staple oligomers (Supplementary Fig. 3). These results indicate that rG4 induced by Staple oligomers could have a parallel topology.

rG4 inhibits not only translation but also the elongation reaction of reverse transcriptase. Hagihara et al. developed the technique for rG4 detection and location (the RTase stop assay) that exploits this mechanism and reported various rG4 identification experiments^36,37^. We further evaluated rG4 induction activity of the DNA Staple oligomers by the RTase stop assay. The results showed that the 40-nt Staple oligomers induced rG4 formation at the expected position in the sequences of all rG4 models in the presence of KCl, but not in the presence of LiCl (Fig. 1f and Supplementary Fig. 4). Notably, the 26-nt Staple oligomers failed to exhibit rG4 induction activity, even in the presence of KCl (Fig. 1f). This suggests that nucleotide length is important in the development of Staple oligomers conducive to rG4 induction on the target RNA (Fig. 1g and Supplementary Table 2).

We next evaluated the translation inhibition activity of Staple oligomers by in vitro translation. For the in vitro translation experiments, we prepared dual reporter genes in which the translation of Firefly luciferase (FL) is controlled by the model RNAs with 63-, 100- or 140-nt-long loops at the 5′ UTR, while Renilla luciferase (RL) translation mediated by an internal ribosomal entry site (IRES) is used as an internal standard (Fig. 1h). Comparison of the luminescence signals from FL and RL allowed us to quantify the effects of the rG4 structure in 5′ UTRs on translation. The 40-nt DNA Staple oligomer notably suppressed gene expression for all rG4 model sequences, while the oligonucleotide that simply hybridized to the target (hybridization oligomer) showed no detectable suppression of gene expression despite the length of it being the same as the Staple oligomers (Fig. 1i). These results suggest that Staple oligomers would be a promising nucleic acid medicine that suppresses target gene expression.

### Application of RNAh technology to TRPC6 gene

**Fig. 2:**
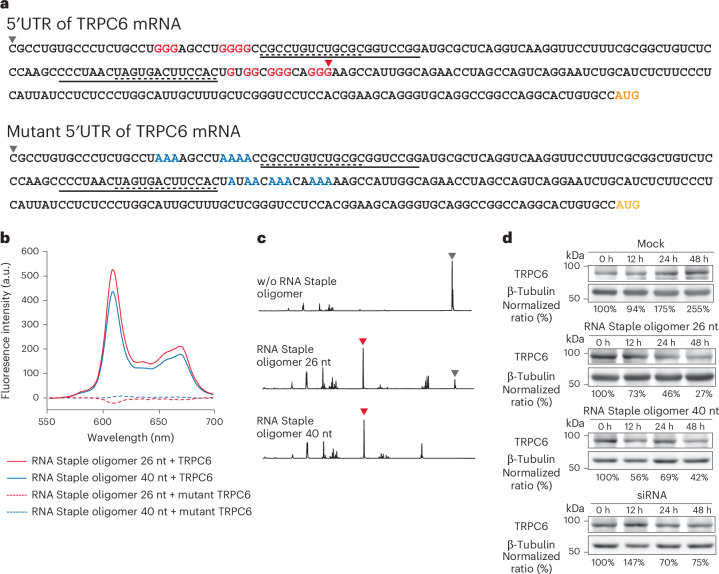
In vitro application of RNAh technology to the TRPC6 gene. **a**, Nucleotide sequences of the 5′ UTR of TRPC6 mRNA and its synthetic control (mutant). The G-tracts are shown in red. The A-tracts, which replace the G-tracts, are shown in blue. The start codon (AUG) is shown in orange. The red arrowheads indicate the site of arrest of RTase with rG4 induced by the Staple oligomer and black arrowheads indicate the end of RTase elongation without rG4 induction. The dashed and solid underlines represent the 26-nt or 40-nt Staple oligomer recognition sites, respectively. **b**, Evaluation of rG4 formation using NMM fluorescent probe. The red and blue curves show the fluorescence emission spectra of NMM in the presence of 26-nt or 40-nt RNA Staple oligomers, respectively. The solid and dashed curves represent the 5′ UTR of TRPC6 and its mutant, respectively. **c**, Identification of rG4 formation on the 5′ UTR of TRPC6 mRNA by the RTase stop assay. RTase-mediated cDNA synthesis was interrupted on the 5′ UTR in the presence of each RNA Staple oligomer. The red and black arrowheads indicate the sites of arrest of RTase with rG4 induced by Staple oligomer and RTase elongation ends without rG4 induction, respectively. **d**, Evaluation of the effects of RNA Staple oligomers and siRNA on TRPC6 expression in C2C12 cells by western blotting. Protein expression levels were quantified relative to the expression of β-tubulin using ImageJ software. Source data.

Upon evaluating the preliminary results, we applied the present technology to the myocardial hypertrophy-related transient receptor potential cation channel subfamily C member 6 (TRPC6) gene (Fig. 2a)^38,39^, which is highly homologous to another gene of the same subfamily, TRPC3, and is difficult to target with existing nucleic-acid-based technologies (Supplementary Fig. 5). For the initial trial, to find suitable target sequence of Staple oligomer for TRPC6 gene regulation, we prepared two 40-nt DNA Staple oligomers for TRPC6 mRNA and evaluated the in vitro activities (Supplementary Fig. 6a,b). From the results, we selected hybridization sites on 5′ UTR of TRPC6 mRNA for the Staple oligomer (Fig. 2a). For the next trial, to confirm the induction of rG4 by the RNA Staple oligomer, we conducted a fluorescent study using NMM. When the RNA Staple oligomer was added to the solution of 5′ UTR of mouse TRPC6 mRNA, a remarkable fluorescent signal was observed, showing the formation of rG4 (Fig. 2b). The Staple oligomer length dependence of the efficiency of reverse transcription inhibition for the TRPC6 gene was also determined by the RTase stop assay^36,37^. The results showed that the 26-nt-long RNA Staple oligomer is sufficient for inhibiting reverse transcription (Fig. 2c, Supplementary Fig. 7 and Supplementary Table 3).

The translation inhibition activity of the 26-nt and 40-nt RNA Staple oligomers was evaluated by in vitro translation. For the in vitro translation experiments, we prepared two FL–RL dual reporter genes in which the translation of FL is controlled by the 5′ UTR of TRPC6 mRNA (Supplementary Fig. 8a). Cell-free translation experiments of the reporter gene revealed that the Staple oligomers reduced the translational efficiency of the transcripts bearing the TRPC6 5′ UTR by approximately 80%, while the Staple oligomers exhibited no detectable effects on the translation from its control sequence with A-tracts that is unable to form rG4 (Supplementary Fig. 8b). These results are consistent with the results of the fluorescent study using NMM for model sequences, showing that the Staple oligomer-induced translational suppression is mediated by the induction of rG4.

To validate the versatility of the RNAh technology, the possibility of regulating the expression of cancer-related genes, TPM3 and MYD88, was examined using the dual reporter genes incorporating their 5′ UTRs^40,41^. The results revealed the successful confirmation of the suppressive effect of RNAh on the expression of the cancer-related genes, demonstrating that RNAh is a very versatile technology (Supplementary Fig. 8c–e). This technology functions even in a coding sequence. Indeed, Staple oligomers successfully suppressed translation from a Nano luciferase reporter gene that was forcibly mutated to possess four G-tracts (Supplementary Fig. 9). These findings highlight the potential of RNAh technology to target a broad range of mRNAs. Further potential versatility of RNAh technology was validated by the GGG bioinformatic study. We restricted our gene count to those with loops between consecutive G-tracts that are within 200 nucleotides in length. Bioinformatic studies revealed 39,202 genes potentially suitable for the 2 + 2-type motif and 34,212 genes for the 3 + 1-type motif, out of a total of 67,673 genes. In total, 44,237 genes were identified to possess either a 2 + 2-type or a 3 + 1-type motif, with 29,177 genes containing both motif types. Conversely, 14,282 genes have neither a 2 + 2-type nor a 3 + 1-type motif. These findings indicate that approximately 65% of known human mRNAs could serve as potential targets for RNAh technology. However, not all candidate target sites are accessible with Staple oligomers, which may constrain the number of feasible targets for practical application. Despite this limitation, RNAh technology appears to have broad potential for application to a variety of disease-related genes.

On the basis of these results, we applied the RNAh technology to endogenous TRPC6 mRNA in living C2C12 cells. The inhibition of TRPC6 expression in living cells by Staple oligomers was validated by western blot analysis. For this experiment, short RNA expression vectors encoding 26- or 40-nt RNA Staple oligomers were employed for expression within the cells (Supplementary Fig. 10a). Western blotting revealed that TRPC6 expression was reduced in a time-dependent manner in both cases where Staple oligomers were introduced (Fig. 2d). These results are consistent with those of the in vitro experiments. Under our conditions of the C2C12 cell-based experiments, in particular, the suppressive effect of the Staple oligomer on TRPC6 protein expression was comparable to that of previously reported siRNAs (siRNA-1, 5′-UUA ACA UUG AGG GAA UGA C-3′; siRNA-2, 5′-UAA UCU UCU GAG CUC CUU G-3′; siRNA-3, 5′-UUC UAA UGA GCU CUG CUA G-3′) whose mixture is known to knock down 60% of the TRPC6 gene expression (Fig. 2d)^42^. The versatility of the RNAh technology in living cells was also demonstrated in its application to the cancer-related gene TPM3 (Supplementary Fig. 10b).

### Application of RNAh technology in mice

**Fig. 3:**
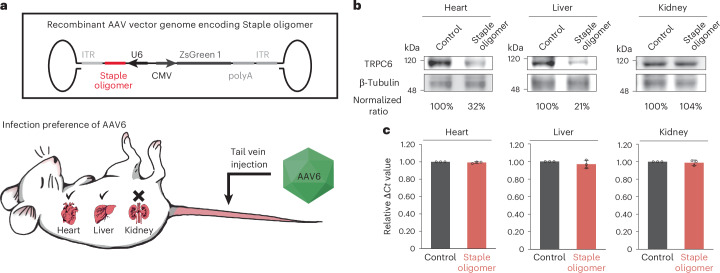
Effect of RNA Staple oligomer on TRPC6 gene expression in mice. **a**, A schematic of the recombinant AAV6 vector genome encoding the Staple oligomer. A short hairpin RNA expression vector was used to express the RNA Staple oligomer. The U6 promoter, Staple oligomer and inverted terminal repeat (ITR) are shown in black, red and grey, respectively. AAV6 infection was confirmed by ZsGreen expression with CMV promoter. The RNA Staple oligomer is expressed by the AAV6 expression system in target organs. AAV6 infects the heart and liver, but not the kidney. **b**, Evaluation of protein expression levels of TRPC6 in each organ by western blotting. TRPC6 expression was suppressed by 70–80% in the heart and liver by Staple oligomers, but not in the kidney, which is consistent with the infection directedness of AAV6. **c**, Evaluation of TRPC6 mRNA expression levels in each organ by qPCR. No appreciable change in the mRNA expression levels was observed in each organ by the introduction of RNA Staple oligomers, clearly supporting the mechanism of action of RNAh that can suppress TRPC6 expression without mRNA degradation. Data are mean ± s.d. of three independent experiments. Source data.

We next applied RNAh to the endogenous TRPC6 gene in mice. For this in vivo experiment, we constructed a system to express the 26-nt Staple oligomer in mice by introducing a short hairpin expression vector into adeno-associated virus 6 (AAV6) (Fig. 3a). AAV6 is known to specifically infect the heart and liver and deliver the expression vector, but this does not occur for the kidney^43,44^. AAV was administered intravenously to mice by injection into the tail. To evaluate the efficiency of expression suppression by RNAh in each organ, the expression level of TRPC6 was verified by western blot analysis. As expected, TRPC6 expression was notably suppressed in the heart and liver, but no detectable change in its expression was observed in the kidney (Fig. 3b). Notably, no appreciable difference in the amount of TRPC6 mRNA was observed by qPCR with or without the introduction of Staple oligomer by AAV6 (Fig. 3c). A theoretical explanation for this is that siRNA functions by decreasing target mRNA, whereas RNAh works by inducing a stable higher-order structure in the target mRNA without digesting the mRNA. The qPCR results provide strong evidence of the mechanism of action of the RNAh technology.

### Prevention of myocardial hypertrophy in TAC-treated mice

**Fig. 4:**
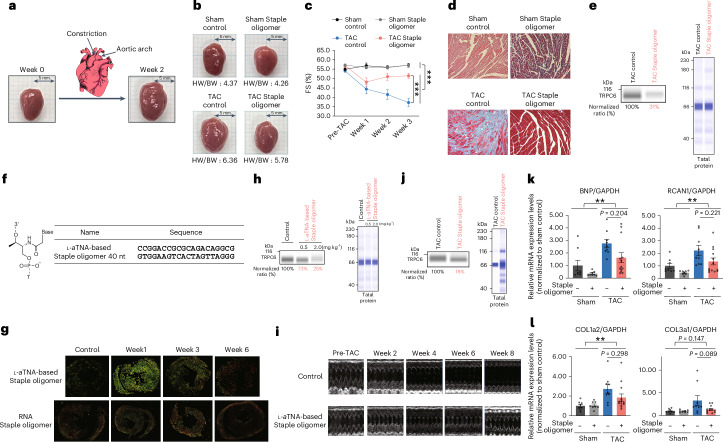
Effects of Staple oligomers on myocardial hypertrophy. **a**, Stenosis of the aortic arch of the heart causes myocardial hypertrophy. **b**, Representative heart images from sham- and TAC-treated mice infected with control-AAV6 or Staple oligomer-AAV6. HW, heart weight; BW, body weight. **c**, Echocardiography revealed that RNA Staple oligomers maintained cardiac function. Data are shown as mean ± s.e.m. (*n* = 9 mice/sham group, *n* = 12 mice/TAC control or *n* = 13 mice/TAC Staple). Statistical significance was determined by two-way ANOVA (two sided, TAC versus sham; ***P = 0.0000103, TAC Staple versus TAC control; ***P = 0.000499). **d**, Evaluation of myocardial fibrosis by Masson’s staining (blue area: fibrosis in the heart). **e**, Western blot analysis of TRPC6 expression in mouse heart. **f**, Chemical structure of acyclic l-*a*TNA and base sequence of l-*a*TNA-based Staple oligomer. **g**, Cardiac delivery of l-*a*TNA-based or RNA Staple oligomers. The top and bottom display the fluorescent signals of heart sections 1, 3 and 6 weeks after transfection with fluorescently labelled l-*a*TNA-based and RNA Staple oligomers, respectively. **h**, Evaluation of effects of l-*a*TNA-based Staple oligomers on TRPC6 expression in mouse hearts. **i**, Echocardiography showed no notable decrease in cardiac function after TAC treatment in l-*a*TNA-based Staple oligomer-treated mice (bottom), but a marked decrease in FS values in control mice (top) up to 8 weeks. **j**, Evaluation of TRPC6 expression in mouse heart 6 weeks after transfection with l-*a*TNA-based Staple oligomers. **k**, qPCR evaluation of mRNA expression levels of BNP and RCAN1, known as cardiac hypertrophy marker. TAC treatment increased mRNA expression (blue), but l-*a*TNA-based Staple oligomers suppressed the increase in mRNA expression (red). Data are shown as mean ± s.e.m. (*n* = 8 mice/sham group, *n* = 9 mice/TAC control or *n* = 13 mice/TAC staple). Statistical significance was determined by two-way ANOVA (two sided, BNP; **P = 0.00571, RCAN1; **P = 0.00357). **l**, qPCR evaluation of mRNA expression levels of COL1a2 and COL3a1, known as myocardial fibrosis markers. TAC treatment increased mRNA expression (blue), but l-*a*TNA-based Staple oligomers suppressed the increases (red). Data are shown as mean ± s.e.m. (*n* = 8 mice/sham group, *n* = 9 mice/TAC control or *n* = 13 mice/TAC Staple). Statistical significance was determined by two-way ANOVA (two sided, **P = 0.00185). TRPC6 signals in all western blot analyses were normalized to total protein. Source data.

The TRPC6 gene is a potential therapeutic target for heart failure associated with myocardial hypertrophy^45–47^. To examine the therapeutic effects of Staple oligomers on cardiac hypertrophy, transverse aortic constriction (TAC)-treated mice, which overexpress TRPC6 and develop myocardial hypertrophy, were used as models (Fig. 4a)^38,46^. The 26-nt Staple oligomers for RNAh were delivered to mouse hearts by AAV6 infection. As expected, the hearts from TAC-treated mice had significantly increased myocardial size and weight compared with those of mice without TAC treatment (sham). Remarkably, the hearts of TAC-treated mice treated with Staple oligomers, however, did not show as great an increase in myocardial weight as those of mice in the group without Staple oligomer treatment (Fig. 4b and Supplementary Fig. 11a,b). Echocardiographic evaluation of cardiac function showed no significant decrease in cardiac function after TAC treatment in the Staple oligomer-treated mouse group, but a significant decrease in fractional shortening (FS) values, which measure the percentage change in left ventricular diameter during heart systole, in the non-Staple oligomer-treated mice (Fig. 4c and Supplementary Fig. 11c). The results confirmed that Staple oligomers suppressed the decline in values reflecting cardiac function. We next evaluated the inhibitory effect of Staple oligomers on myocardial fibrosis by Masson’s trichrome staining. Staple oligomers had no effect on myocardial fibrosis in the sham group, whereas they notably reduced the myocardial fibrosis in the TAC-treated mice (Fig. 4d and Supplementary Fig. 12). Simultaneously, the expression level of TRPC6 was verified by western blot analysis. TRPC6 expression was notably suppressed by Staple oligomers in the hearts of TAC-treated mice (Fig. 4e). We also evaluated the mRNA expression levels of cardiac hypertrophy marker genes ANP, BNP and RCAN1 by qPCR in the TAC-treated hearts (Supplementary Fig. 13). Expression of the selected markers was increased by TAC treatment, but Staple oligomers markedly suppressed these increases. These observations strongly suggest that Staple oligomers contribute to maintain cardiac function in TAC-treated mice by suppressing TRPC6 expression. To evaluate the effects of Staple oligomers on gene expression, mRNA-seq analysis was performed in TAC-treated mice. The expression levels of cardiac fibrosis-related genes were significantly reduced with Staple oligomer treatment (Supplementary Fig. 14). This result strongly supports the suppressive effect on myocardial fibrosis indicated by Masson’s staining.

### Improvement of in vivo stability of Staple oligomer using artificial nucleic acids

Given the potential of RNAh technology, future work should be aimed at overcoming the issue of low in vivo stability inherent in nucleic acid medicine. RNAh technology does not require cooperation with bioprocesses, such as enzymatic reactions, permitting simple replacement of nucleic acids in Staple oligomers with artificial ones to improve their in vivo stability without compromising efficiency. To demonstrate such chemical optimizability of the Staple oligomer, we conducted a series of in vitro and in vivo experiments using an acyclic l-threoninol nucleic acid (l-*a*TNA)-based Staple oligomer designed to target the TRPC6 gene (Fig. 4f)^48,49^. As anticipated, rG4 was induced at the TRPC6 target sequence by a 40-nt l-*a*TNA-based Staple oligomer (Supplementary Figs. 6c,d and 15a,b). This outcome strongly implies that only the sequence-selective hybridization properties of Staple oligomers are crucial for Staple oligomer activity, but not the chemical properties of nucleotide units. Furthermore, the translation inhibition activity of the l-*a*TNA-based Staple oligomer was also evaluated by in vitro translation (Supplementary Fig. 15c,d) and western blot analysis for endogenous TRPC6 expression in C2C12 cells (Supplementary Fig. 15e). As expected, cell-free translation was remarkably inhibited (Supplementary Fig. 15d) and endogenous TRPC6 expression in C2C12 cells was suppressed in a concentration-dependent manner (Supplementary Fig. 15e). These findings provide compelling evidence for the exceptional properties of Staple oligomers, paving the way for the development of entirely non-natural nucleic acid therapeutics.

In a subsequent step, we applied the 40-nt l-*a*TNA-based Staple oligomers to the endogenous TRPC6 gene in mice. The l-*a*TNA-based Staple oligomers were administered directly into mouse hearts via intravenous injection, using an in vivo transfection reagent. Although not selectively delivery to the heart in principle, the l-*a*TNA-based Staple oligomers were successfully delivered to cardiac cells (Fig. 4g and Supplementary Fig. 16). Notably, the Cy5 and fluorescein fluorescence signals of the injected double-fluorescently labelled l-*a*TNA-based Staple oligomers remained highly co-localized in heart sections for up to 6 weeks, indicating that these oligomers were stable in hearts for 1 week and then gradually eliminated without detectable degradation for up to 6 weeks. Conversely, fluorescence co-localization derived from RNA Staple oligomers was disrupted within 1 week, indicating degradation within the mouse heart (Fig. 4g and Supplementary Fig. 16). Indeed, a single injection of l-*a*TNA-based Staple oligomers effectively suppressed TRPC6 expression in mouse hearts for up to 5 weeks in a dose-dependent manner (Fig. 4h and Supplementary Fig. 17). In TAC-treated mouse hearts, the l-*a*TNA-based Staple oligomers maintained cardiac function by suppressing TRPC6 expression (Fig. 4i,j and Supplementary Fig. 18), prevented an increase in myocardial weight (Supplementary Fig. 19a), reduced myocardial fibrosis (Supplementary Fig. 19b) and markedly suppressed mRNA expression of cardiac hypertrophy marker genes and myocardial fibrosis marker genes (Fig. 4k,l and Supplementary Fig. 20), as did RNA Staple oligomers introduced with AAV6. These outcomes strongly indicate that our method possesses remarkable potential to enable the development of a fully non-natural nucleic acid medicine in a quite simple manner. The unique attributes offered by this approach cannot be achieved through any other existing method, including RNAi technology.

## Discussion

Here, we report RNAh technology as a promising nucleic acid-based therapeutic approach, following in the footsteps of RNAi and antisense technologies. The RNAh technology can be leveraged when a Staple oligomer accurately recognizes two sequences that are apart from each other and subsequently induces an rG4 structure on a target mRNA. rG4 induction, however, requires a certain length of the Staple oligomer, implying that rG4 formation should not occur even if a Staple oligomer binds to homologous off targets with G-tracts. In length optimization of the Staple oligomer, its short hybridization substantially reduced the induction of rG4 formation (Fig. 1g and Supplementary Fig. 7). Thus, this cooperative gene regulation technology, combining target-specific hybridization and high-order structural induction, enables precise, personalized and safe gene therapy.

In our investigation, Staple oligomers demonstrated robust gene suppression activity against their specific targets both in vitro and in mice. In particular, the efficacy of downregulation of RNAh targeting TRPC6 was higher than that by siRNAs in mouse C2C12 and NIH3T3 cells (Fig. 2d and Supplementary Fig. 21). Additionally, we conducted a comparative assessment of the in vivo potency of Staple oligomers and siRNAs using an AAV approach to evaluate their effects on TRPC6 expression in the mouse heart (Fig. 3b and Supplementary Fig. 22). The results revealed that Staple oligomers exhibited more effective suppression of TRPC6 gene expression than siRNAs, consistent with the outcomes in our cell experiments. However, these findings do not conclusively establish RNAh technology as inherently superior to siRNA technology for gene silencing. It is plausible that our selection of siRNA for the TRPC6 gene in our experiments was suboptimal.

One plausible, advantage of RNAh technology over RNAi or RNaseH mechanisms is the chemical optimizability of Staple oligomers. RNAh technology was validated through in-cell assays to function independently of biological processes such as Argonaute or RNaseH reactions (Supplementary Fig. 23). This independence permits the simple replacement of nucleic acids in Staple oligomers with artificial ones to improve their stability without compromising efficiency (Supplementary Fig. 24). Notably, l-*a*TNA-based or 2′MOE-modified Staple oligomers demonstrated functionality as rG4 inducers similar to DNA or RNA Staple oligomers (Supplementary Figs. 15, 25 and 26) and as translation inhibitors in cells akin to RNA Staple oligomers, in a dose-dependent manner (Supplementary Figs. 15e and 25d). DNA Staple oligomers were not used in cellular or in vivo experiments owing to their instability within cells. However, in vitro observations suggest that DNA Staple oligomers may indeed cooperate with RNaseH in cells (Supplementary Fig. 27). The advantage of our technology over steric inhibition mechanisms lies in its high target specificity and potent translation inhibition efficacy. Steric blockers relying solely on hybridization may lead to off-target effects due to mishybridization. Moreover, G-quadruplex formation induced by Staple oligomers may be more efficient than simple hybridization of steric inhibitors in translation inhibition. Indeed, l-*a*TNA-based Staple oligomers have exhibited notable inhibition of protein translation only in RNA targets with G-quadruplex-forming sequences (Supplementary Fig. 15d). This observation indicates that the steric blocking effect of l-*a*TNA-based staple oligomers on translational reactions is much less than the effect of G-quadruplex formation. These results suggest that simple hybridization of steric blockers might not be as effective as G-quadruplex induction of RNAh technology in inhibiting ribosomal reactions.

The development of modified oligonucleotide drugs that are non-toxic and exhibit efficient and specific activity in vivo has also remained a longstanding challenge in this field. We observed that rG4 was induced on targets by DNA-based, RNA-based, 2′MOE-based (commonly used in clinical nucleic acid drug modification) and l-*a*TNA-based Staple oligomers (Supplementary Fig. 26). This means that it is possible to select any artificial nucleotide to develop non-toxic nucleic acid medicines. The present study, however, leaves room for further discussion. The optimal length of Staple oligomers for DNA was different from that for RNA (Supplementary Fig. 7), possibly due to the differences in the intramolecular structure or hybridization stability of each Staple oligomer. For drug discovery, the optimization of base length would be a necessary process when introducing artificial nucleotides in Staple oligomers.

A common and critical challenge faced by those developing nucleic acid drugs, including RNAh technology, is how to effectively deliver them to target organs or cells. In the in vivo experiments described here, RNA Staple oligomers were highly expressed using AAV6-mediated infection. The delivery of RNA Staple oligomers to target organs and cells relied on the infection preference of AAV6. Meanwhile, l-*a*TNA-based Staple oligomers were introduced simply by using an in vivo transfection reagent, without addressing the delivery challenge in RNAh technology at this stage. One possible solution to this issue would be to combine the present technology with appropriate drug delivery systems^50–53^. If two distinct Staple oligomers targeting different mRNAs can be introduced, it may be possible to simultaneously inhibit the translation of both transcripts. The unique advantages of Staple oligomers, including high target selectivity and low toxicity, could provide promising opportunities for the development of therapeutic strategies.

In this study, RNAh technology was introduced as a method for translation inhibition. Staple oligomers function by specifically recognizing the target RNA and inducing conformational changes, particularly promoting the formation of rG4 structures. When applied to noncoding RNA targets, Staple oligomers can alter the conformation of the target RNA, which might subsequently inhibit its maturation and, thereby, suppress the functional activity of the noncoding RNA. One limitation of applying this technology to noncoding RNA targets is the difficulty in predicting the relationship between RNA structure and maturation. However, if this limitation can be addressed, RNAh technology could offer a promising strategy for the development of nucleic acid-based therapeutics to noncoding RNA targets.

## Methods

### Oligonucleotides

All oligonucleotides were purchased from Thermo Fisher Scientific or Eurofin Genomics. DNA oligonucleotides were used as Staple oligomers, primers and templates for PCR and primers for qPCR. RNA oligonucleotides were used as Staple oligomers. Individual DNA and RNA oligonucleotides were stored at a concentration of 100 µM in TE buffer. The l-*a*TNA-based and 2′MOE-modified Staple oligomers were purchased from Hokkaido System Science.

### ThT fluorescence measurements

A mixture of template RNA (4 µM for the model sequence, 0.4 µM for the 5′ UTR of the TRPC6 gene) and Staple oligomer (5 µM for the model sequence, 0.5 µM for the 5′ UTR of the TRPC6 gene) in a buffer (20 mM Tris–HCl pH 7.8 and 100 mM KCl) was heated to 90 °C for 2 min and then gradually cooled to 20 °C at a rate of 1.0 °C min^−1^. For measurement, ThT (Sigma) was added to the mixture at 1 µM for the model sequence and 50 µM for the 5′ UTR of the TRPC6 gene and incubated for 30 min in the dark at room temperature. Fluorescence intensity was measured with a Cary Eclipse fluorescence spectrophotometer (Agilent Technologies) or FP-8500 fluorescence spectrophotometer (JASCO). The excitation wavelength was 440 nm. The fluorescence spectra were obtained by taking the moving average of five points made at 1.0 nm intervals from 450 to 600 nm. All of the fluorescence curves were normalized by subtracting data from a control experiment without adding Staple oligomer.

### NMM fluorescence measurements

A mixture of template RNA (3 µM for the model sequence and Nano luciferase gene, 30 µM for the 5′ UTR of the TRPC6 gene) and Staple oligomer (3.75 µM for the model sequence and Nano luciferase gene, 37.5 µM for the 5′ UTR of the TRPC6 gene) in a buffer (20 mM Tris–HCl pH 7.8 and 100 mM KCl) was heated to 90 °C for 2 min and then gradually cooled to 20 °C at a rate of 1.0 °C min^−1^. For measurement, NMM (GlpBio) was added to the mixture at 1 µM and incubated for 30 min in the dark at room temperature. Fluorescence intensity was measured with a Cary Eclipse fluorescence spectrophotometer (Agilent Technologies). The excitation wavelength was at 399 nm. The fluorescence spectra were obtained by taking the moving average of five points made at 1.0 nm intervals from 550 to 700 nm. All of the fluorescence curves were normalized by subtracting data from a control experiment without adding a Staple oligomer.

### Thermal denaturation profiles

Melting experiments were conducted with target RNA and 5′-FAM-labelled Staple (5′-FAM-TTG CAT TAA TCT AGG TCG ACA AGC TTC AAT TGG GTG CTG G-3′) hybrids (template: 0.5 μM, Staple: 0.5 μM) in 50 mM Tris–HCl buffer (pH 8.0) containing 100 mM KCl. The fluorescence intensity of the samples was monitored from 20 °C to 100 °C with a heating rate of 1.0 °C min^−1^ with the CFX Connect Real-Time System (Bio-Rad). The RNA Staple hybrids were heated to 80 °C and subsequently cooled to ambient temperature before measurements. The melting curves were converted to distinct melting peaks by plotting the first negative derivative of the fluorescence as a function of temperature (−*dF*/*dT*).

### RTase stop assay for rG4 model sequence

A reaction mixture of template RNA (0.1 µM) and a 5′-FAM-labelled primer (5′-FAM-CGC CAG GGT TTT CCC AGT CAC GAC-3′) (0.03 µM) was heated to 80 °C for 3 min in folding buffer (50 mM Tris–HCl pH 8.0, containing 100 mM KCl) and then cooled to 30 °C for 30 min, followed by the addition of Staple oligomer (1.0 µM) for 30 min at room temperature. ReverTra Ace reverse transcriptase (Toyobo), MgCl_2_ (5 mM) and dNTPs (1.0 mM) were then added to the reaction mixture and the reaction was carried out at 42 °C for 30 min, after which the reaction was stopped by heating it at 99 °C for 5 min. The produced cDNAs were analysed on a 3500 Genetic Analyzer (Applied Biosystems).

### RTase stop assay for TRPC6 and Nano luciferase gene

A reaction mixture of template RNA (0.3 µM), Staple oligomer (1 µM) and a 5′-FAM-labelled primer (5′-FAM-CGC CAG GGT TTT CCC AGT CAC GAC-3′) (0.1 µM) was heated to 90 °C for 2 min in folding buffer (50 mM Tris–HCl pH 7.8, containing 150 mM KCl for the Nano luciferase gene or containing 150 mM KCl and polyethylene glycol of 6.7 wt% for the TRPC6 gene) and then cooled to 20 °C at a rate of 1.0 °C min^−1^. ReverTra Ace reverse transcriptase (Toyobo), MgCl_2_ (5 mM) and dNTPs (1.0 mM) were then added to the reaction mixture and the reaction was carried out at 42 °C for 30 min, after which the reaction was stopped by heating it at 99 °C for 5 min. The produced cDNAs were analysed on a SeqStudio Genetic Analyzer (Applied Biosystems). GeneMapper Software 6 was used to analyse RTase Stop assay fragments.

### In vitro translation assay

A reaction mixture of template RNA (3.72 pmol, or 14.00 pmol for the Nano luciferase gene), with or without Staple oligomer (4.65 pmol, or 42.00 pmol for the Nano luciferase sequence) and a buffer (50 mM Tris–HCl pH 7.8, 100 mM KCl) were heated to 90 °C for 2 min, gradually cooled to 20 °C at a rate of 1.0 °C min^−1^. The samples were used as mRNA templates in 25 μl of cell-free protein expression mixture (RTS 100 Wheat Germ CECF kit, 5 Prime), with or without Staple oligomer for 150 min at 24 °C. Luciferase activity was evaluated using the Luciferase assay kit (Promega) and a POWERSCAN H1 microplate reader (BioTek).

### Degradation assay of target RNA/DNA Staple oligomer with RNase H

A mixture of template RNA (0.3 µM) and Staple oligomer (1 µM) in a buffer (50 mM Tris–HCl pH 7.8 and 150 mM KCl) was heated to 90 °C for 2 min and then gradually cooled to 20 °C at a rate of 1.0 °C min^−1^. MgCl_2_ (5 mM) and RNaseH (10 U, TaKaRa Bio) were then added to the reaction mixture and the reaction was carried out at 30 °C for 30 min, after which the reaction was stopped by heating it at 80 °C for 2 min. The RNA mixture treated with RNase H was analysed by 8% denaturing PAGE.

### Stability test of Staple oligomer with 10% FBS

For this test, 5 µM DNA, RNA and l-*a*TNA-based or 2′MOE-modified Staple oligomers were mixed with 10% (v/v) fetal bovine serum (FBS). After reacting at 37 °C in a humidified 5% CO_2_ incubator for a given period of time, the reaction mixtures were analysed by 10% denaturing PAGE.

### CD spectroscopy

CD experiments were performed using a J-1100 CD spectrometer (JASCO) using a cell with a 0.1 cm path length. Samples were prepared by heating the oligonucleotides at 80 °C for 3 min and gradually cooling them to room temperature. CD spectral changes of RNA oligonucleotides duplex or RNA–DNA hybrids (2 µM) were measured in a buffer containing 50 mM Tris–HCl buffer (pH 8.0) containing 100 mM KCl. Spectra were collected in the range between 190 and 350 nm, as a sum of ten repetitions and the buffer baseline was subtracted from each spectrum.

### In-cell translation assay

HEK293T cells were maintained in medium A (Dulbecco’s modified Eagle’s medium, supplemented with 100 units ml^−1^ penicillin, 100 mg ml^−1^ streptomycin sulfate and 10% (v/v) FBS). All cell lines were cultured at 37 °C in a humidified 5% CO_2_ incubator. Approximately 2 × 10^4^ HEK293T cells were seeded in flat-bottomed 96-well plates and incubated overnight. The following day, the cells were co-transfected with the Staple oligomer 40 nt-expressing plasmid and the pIRES-rG4-model-2 + 2-100nt-NL-FL-expressing plasmid or pIRES-Control-model-2 + 2-100nt-NL-FL-expressing plasmid using FuGENE HD (Promega) as a transfection reagent in accordance with the manufacturer’s instructions. Twenty-four hours later, the cells were lysed with 20 μl of Passive Lysis Buffer (Promega). Then, 8 μl of lysate from each well was transferred to a 96-well flat-bottomed white assay plate (Thermo Fisher). Luciferase activity was evaluated using the Luciferase assay kit (Promega) and BioTek Synergy H1 Microplate Reader (Agilent Technologies).

### Cell cultures and transfection

C2C12, NIH3T3 and MCF-7 cells were maintained in medium A (Dulbecco’s modified Eagle’s medium, supplemented with 100 units ml^−1^ penicillin, 100 mg ml^−1^ streptomycin sulfate and 10% (v/v) FBS) and medium B (Eagle’s minimum essential medium, supplemented with 100 units ml^−1^ penicillin, 100 mg ml^−1^ streptomycin sulfate and 10% (v/v) FBS), respectively. All cell lines were cultured at 37 °C in a humidified 5% CO_2_ incubator. Each Staple oligomer-expression vector, siRNA-expression vector, l-*a*TNA-based Staple oligomer and 2′MOE-modified Staple oligomer was transfected into the cells using FuGENE HD (Promega) according to the manufacturer’s instructions.

### Animal studies

Male BALB/c and C57BL/6J mice were purchased from Oriental Yeast Co. Ltd. and Clea Japan Inc., respectively. All mice were maintained under a 12:12 h light–dark cycle, temperature at 24 °C, ~40–45 % humidity with ad libitum access to a regular chow diet and water. BLAB/c (6-week-old) or C57BL/6 J (7-week-old) mice were used for in vivo translation assay or for phenotype evaluation, respectively. Pressure overload was produced by TAC. Sham-operated mice underwent the same operation, but without aortic constriction. All animal procedures were performed in accordance with Kumamoto University animal care guidelines and the Guide for the Care and Use of Laboratory Animals published by the by the US National Institutes of Health (publication no. 85-23, revised 1996) and permitted by the Animal Care and Use Committee of Kumamoto University.

### AAV generation and mouse transduction

pAAV-U6-ZsGreen1 vector (5 μg), Staple oligomer-expression construct (5 μg) or siRNA-expression construct (5 μg), pRC6 vector (5 μg) and pHelper vector (5 μg) were mixed for co-transfection of HEK293T cells in Optimem medium (Thermo Fisher Scientific) using TransIT-VirusGEN Transfection Reagent (Mirus). At 3 days after transfection, Staple oligomer-expression or control AAVs were extracted from the cells and purified using AAVpro purification kit Maxi (all serotype) (Takara Bio) according to the manufacturer’s protocol. The purified AAV titres were determined using the AAVpro Titration kit Ver.2 (Takara Bio) for real time PCR according to the manufacturer’s protocol. For tail vein injections of AAVs, the mice were anesthetized with isoflurane (Pfizer). For the in vivo translation assay. BALB/c mice were injected in the tail vain with 1 × 10^9^ vg of AAVs in 450 μl of PBS. For phenotypic and siRNA evaluation, C57BL/6J mice were injected in the tail vain, with 1.5 × 10^10^ vg of AAVs in 450 μl of PBS.

### Administration of l-*a*TNA-based Staple oligomer

l-*a*TNA-based Staple oligomer was in vivo transfected into mice using an AteloGene Systemic Use kit (Koken) with atelocollagen. Before tail vein injections of l-*a*TNA-based Staple oligomer, the mice were anesthetized with isoflurane (Pfizer). The C57BL/6J mice were injected with l-*a*TNA-based Staple oligomer (0.5 or 2.0 mg kg^−1^ in PBS) or PBS (control).

### Fluorescent evaluation of cardiac delivery of l-*a*TNA-based or RNA Staple oligomer

For preparation of cardiac section after in vivo transfection of l-*a*TNA-based or RNA Staple oligomer, a mid-transverse cross-section of the heart encompassing both the left and right ventricle, was dissected, fixed overnight in 4% paraformaldehyde, embedded in paraffin, cut into 3-μm-thick sections and mounted by ProLong Gold Antifade Mountant (Thermo Fisher Scientific). Fluorescent signals of the heart sections were observed with a widefield and confocal imaging system (Mica, Leica). The excitation laser wavelength and emission filter were as follows: excitation 495 nm and emission 519 nm for detection of FITC; excitation 649 nm and emission 666 nm for detection of Cy5.

### Echocardiography

In vivo cardiac function was assessed by serial echocardiography in M-mode on an Aplio300 (Canon Medical Systems). Left ventricular internal dimension in diastole, left ventricular internal dimension in systole and left ventricle fractional shortening were determined and then calculated by the average of two points from independently obtained M-mode images. All parameters were determined by an observer blinded to condition.

### Tissue collection and histology

The heart and body weights of the killed mice were recorded at the terminal point of the experiment. For pathological analyses, a mid-transverse cross-section of the heart encompassing both the left and right ventricle was dissected, fixed overnight in 4% paraformaldehyde, embedded in paraffin, cut into 3-μm-thick sections and stained with Masson’s Trichrome. The sections were analysed using an Axio Scope A1 microscope with an Axiocam ERc 5 s camera (Zeiss). Portions of the myocardium were frozen in liquid nitrogen and stored at −80 °C until used for western blotting, RT–qPCR, proteomic and microarray analysis.

### Western blot analysis

In cell translation assay, the cells were washed three times with cold PBS and lysed with LIPA buffer (Nacalai Tesque) containing protease inhibitor cocktail (Nacalai Tesque). The cell lysates were passed ten times through a 25G needle and centrifuged at 4 °C for 10 min. For the in vivo translation assay, the mouse heart, liver and kidney were homogenized with a lysis buffer (Cell Signaling Technology) containing protease inhibitor cocktail (Nacalai Tesque) by using μT-12 bead beater homogenizer (Titec) and SLPe40 ultrasonic homogenizer (BRANSON). The lysates were centrifuged at 4 °C for 30 min to remove debris. The supernatants were transferred to new tubes and mixed with 6× SDS sample buffer (Nacalai Tesque) and then the mixture was heated at 95 °C for 5 min. The samples were separated on a 15% SDS–PAGE and blotted. The blotted protein bands were specifically visualized by antibodies against TRPC6 (16716; Cell Signaling Technology 1:1,000; 18236-1-AP; Proteintech 1:1,000), TPM3 (ab180813; Abcam 1:10.000) or β-tubulin (66240-1-Ig; Proteintech 1:10,000), Signal Enhancer HIKARI for western blotting and ELISA (Nacalai Tesque) and Chemi-Lumi One Super (Nacalai Tesque) on an ImageQuant LAS 500 (GE Healthcare).

### Western blot analysis using Abby protein simple system

A simple western assay was performed in the Abby instrument (Protein Simple) using 12–230 kDa Separation 8 × 25 capillary cartridges (Protein Simple, SM-W004) according to the manufacturer’s protocol. TRPC6 peak was detected by anti-TRPC6 antibody (ACC-017; Alomone Labs 1:50) and Anti-Rabbit Detection Module (DM-001; Protein Simple). A total protein assay using Total Protein Detection Module (DM-TP01; Protein Simple) and Replex Module (RP-001; Protein Simple) was also performed in the same run. TRPC6 peak area was determined using Compass software (Protein Simple) and normalized to that of total protein.

### RT–qPCR

Total RNA was isolated from cells or mouse heart with Isogen (Nippon Gene) according to the manufacturer’s protocol. First-strand cDNAs were prepared by reverse transcription with an oligo (dT) primer (Thermo Fisher Scientific) and ReverTra Ace reverse transcriptase (Toyobo) according to the manufacturer′s protocol. The cDNAs were subjected to qPCR, using a pair of GAPDH primers to quantify mouse GAPDH mRNA (5′-AAC AGC AAC TCC CAC TCT TCC-3′ and 5′-GTG GTC CAG GGT TTC TTA CTC-3′), a pair of TRPC6 primers to quantify mouse TRPC6 mRNA (5′-AAC TCG GGG AGA GAC TG-3′ and 5′-ATA TGG CTT CAA GTG GAG-3′), a pair of ANP primers to quantify mouse ANP mRNA (5′-GGT CTA GTG GGG TCT TGC-3′ and 5′-CGT CTG TCC TTG GTG CTG-3′), a pair of BNP primers to quantify mouse BNP mRNA (5′-CTG GGA CCA CCT TTG AAG TG-3′ and 5′-GTG GCA AGT TTG TGC TCC-3′), a pair of RCAN1 primers to quantify mouse RCAN1 mRNA (5′-TCC AAA CCC TGT TTC CAG-3′ and 5′-CTC TCC GGT TCA AAG TGC-3′), a pair of COL1a2 primers to quantify mouse COL1a2 mRNA (5′-CCC TGA CAC CTG TTG TGG AC-3′ and 5′-GAA GCC ACA AGT GGT GCG-3′), a pair of COL3a1 primers to quantify mouse COL3a1 mRNA (5′-GCC CAT CTG CAG AGC AAC-3′ and 5′-CAT AGG GAG GAG GAC ATG TCT AA-3′), a pair of FN1 primers to quantify mouse FN1 mRNA (5′-ATC TGA CCT GCA GCA CTG TC-3′ and 5′-GGA CAA AGT GAG TCC TGT GGG-3′) and a pair of TIMP2 primers to quantify mouse TIMP2 mRNA (5′-TAA GAC ATA TCC GTG GGC TTG-3′ and 5′-GTT GGG GGG TAC ATC TCA GAC-3′). The qPCR analysis was performed on a MiniOpticon Real-Time PCR System (Bio-Rad Laboratories) with SYBR Green (Toyobo).

### Bioinformatic study on potential G-Quadruplex-forming sequences for RNAh technology in mRNAs

The number of genes potentially applicable for RNAh technology was estimated by means of counting the genes containing G-quadruplex-forming sequences. Huppert et al. reported that an rG4 is formed in the genes whose sequences conform to the (G_3_–N_1–__7_–G_3_–N_1–__7_–G_3_–N_1–__7_–G_3_) rule^29^. Based on the consensus sequence, we used specific regular expression: ‘G_3+_N_1–__7_G_3+_’ (hereafter referred to as motif-2) for the ‘2 + 2’ type, and ‘G_3+_–N_1–__7_–G_3+_–N_1–__7_–G_3+_’ (motif-3) and ‘G_3+_’ (motif-1) for the ‘3 + 1’ type, respectively. To estimate the number of applicable genes for RNAh technology, we counted the genes that have at least two motif-2 sequences or the genes that have both motif-1 and motif-3 sequences using our counting program^54^. A Fasta dataset comprising human mRNA sequences sourced from the National Center for Biotechnology (NCBI)^55^ (mirror server)) served as the reference. Notably, within this dataset, only mRNA sequences whose accession ID commencing with ‘NM_’ (the prefix of mRNA entries) were subjected to analysis.

The gene count was restricted to targets with loops between consecutive G-tracts that are within 200 nucleotides in length. Our analysis revealed 39,202 genes potentially suitable for the ‘2 + 2’ type and 34,212 genes for the ‘3 + 1’ type, out of a total of 67,673 genes. In total, 44,237 genes were identified to possess either ‘2 + 2’ or ‘3 + 1’ type motifs, with 29,177 genes containing both motif types. Conversely, 14,282 genes have neither ‘2 + 2’ nor ‘3 + 1’ type motifs. These findings indicate that approximately 65% of known human mRNAs are potential targets for RNAh technology.

### mRNA-seq analysis

mRNA sequencing was performed by TaKaRa Bio, Inc. Purified RNA was resuspended in Clontech buffers for mRNA amplification by 5′ template switching PCR with a SMART-Seq v4 Ultra Low Input RNA kit (Clontech), in accordance with the manufacturer’s instructions. The amplified cDNA was fragmented and linked with dual-indexed barcodes using Nextera XT DNA Library Prep kits (Illumina). The libraries were validated using a DNA/RNA UD Index, Tagmentation (Illumina), pooled and sequenced on the NovaSeq X Plus platform (Illumina). The transcripts per million values were selected as data representative of gene expression and were calculated using the DRAGEN Bio-IT Platform (Illumina). For normalization, these values were converted to log_2_(transcripts per million).

### Statistics and reproducibility

Results of representative experiments (such as micrographs and western blot) were repeated at least two times independently with similar results. Exact statistical test, *P* values and sample sizes are provided in figure legends. The level of statistical significance is indicated by asterisks (*P < 0.05, **P < 0.01 and ***P < 0.001). All attempts at replication were successful, and each main result was at least a duplicate of experiments.

### Reporting summary

Further information on research design is available in the Nature Portfolio Reporting Summary linked to this article.

## Supplementary information

Supplementary Information Supplementary Figs. 1–27 and Tables 1–3.

Reporting Summary

## Source data

Source Data Figs. 2–4 and Supplementary Figs. 10, 15, 17, 18, 21, 22, 24, 25 and 27 Source data for Figs. 2–4 and Supplementary Figs. 10, 15, 17, 18, 21, 22, 24, 25 and 27.

## Data Availability

The main data supporting the results in this study are available within the Article and its Supplementary information. The TRPC6, TPM3 and MYD88 mRNA sequences are available from the NCBI Nucleotide database, via accession numbers NM_001282086, NM_001278190 and NM_001172566, respectively. Source data are provided with this paper.

## Code availability

The source code used for the bioinformatic analyses is available in the KatsudaGroup/search_g4sites repository^54^, with an archived snapshot available via Zenodo^56^. Instructions for use are provided in the repository’s README.md file. The RefSeq dataset of human mRNA sequences used in this study was downloaded from the NCBI RefSeq database (via the DDBJ mirror) on 3 August 2024 at https://ddbj.nig.ac.jp/public/mirror_database/refseq/H_sapiens/mRNA_Prot/ (ref. ^57^). As the database is subject to ongoing updates, exact results may vary with later versions.
