# Supplementary material for: Staple oligomers induce a stable RNA G-quadruplex structure for protein translation inhibition in therapeutics
Source: Nat Biomed Eng. 2025 Oct 15;10(6):1124–35. doi: 10.1038/s41551-025-01515-4 (PMC13279272; doi:10.1038/s41551-025-01515-4)
Supplement: Supplementary file 1 — Supplementary Figs. 1–27 and Tables 1–3. [file 41551_2025_1515_MOESM1_ESM.pdf]

nature biomedical engineering

Supplementary information

https://doi.org/10.1038/s41551-025-01515-4

Staple oligomers induce a stable RNA G-quadruplex structure for protein translation inhibition in therapeutics

In the format provided by the authors and unedited

Contents

Supplementary Figure 1 | Evaluation of rG4 formation using NMM fluorescent probe. 2
Supplementary Figure 2 | Evaluation of rG4 formation using ThT fluorescent probe. 3
Supplementary Figure 3 | Evaluation of rG4 topology induced by Staple oligomers by Circular dichroism (CD) measurements. 4
Supplementary Figure 4 | Identification of rG4 formation on the rG4 model sequences by RTase stop assay. 5
Supplementary Figure 5 | Nucleotide sequences of 5UTR of TRPC6 and TRPC3 mRNA. 6
Supplementary Figure 6 | Determination of Staple oligomer target sequence for TRPC6 gene. 7
Supplementary Figure 7 | rG4 induction with RNAh technology in various Staple oligomers. 8
Supplementary Figure 8 | Validation of the versatility of RNAh technology with Staple oligomers using in vitro translation assay. 9
Supplementary Figure 9 | Characterization of RNAh technology to Nano luciferase CDS region. 10
Supplementary Figure 10 | Effect of RNAh technology on target protein expression in mammalian living cells. 11
Supplementary Figure 11 | Effects of Staple oligomers on myocardial hypertrophy in the TAC-treated mouse hearts. 12
Supplementary Figure 12 | Effects of Staple oligomers on myocardial hypertrophy by transverse aortic constriction (TAC). 13
Supplementary Figure 13 | Effects of Staple oligomers on gene expression in the TAC-treated mouse hearts. 14
Supplementary Figure 14 | mRNA-seq and proteomic analysis on myocardial hypertrophy. 15
Supplementary Figure 15 | Characterization of RNAh technology with L-aTNA-based Staple oligomer. ...16
Supplementary Figure 16 | Cardiac delivery of L-aTNA-based or RNA Staple oligomer. 17
Supplementary Figure 17 | Effects of L-aTNA-based Staple oligomer on TRPC6 expression in mouse hearts. 18
Supplementary Figure 18 | Effects of L-aTNA-based Staple oligomer on cardiac function in TAC-treated mouse hearts. 19
Supplementary Figure 19 | Effects of L-aTNA-based Staple oligomers on myocardial hypertrophy in TAC-treated mouse hearts. 20
Supplementary Figure 20 | Effects of L-*a*TNA-based Staple oligomers on gene expression in the TAC-treated mouse hearts. 21
Supplementary Figure 21 | Effect of siRNA on TRPC6 protein expression in NIH3T3 cells. 22
Supplementary Figure 22 | Effects of siRNA on TRPC6 expression in mouse hearts. 23
Supplementary Figure 23 | Validation of the cooperation of RNAh technology with RNAi. 24
Supplementary Figure 24 | Evaluation of biological stability of various Staple oligomers under 10% FBS conditions by denaturing PAGE. 25
Supplementary Figure 25 | Characterization of RNAh technology with 2MOE-modified Staple oligomer. 26
Supplementary Figure 26 | Evaluation of concentration dependence with Staple oligomers. 27
Supplementary Figure 27 | Validation of the cooperation of RNAh technology with RNaseH. 28
Supplementary Table 1 | Nucleotide sequences of the target RNA. The solid underlines represent the 40-nt Staple oligomer recognition sites. 29
Supplementary Table 2 | Nucleotide sequences of Staple oligomers for rG4 model sequence, TPM gene, MYD88 gene, and mutated Nano luciferase gene. 30
Supplementary Table 3 | Nucleotide sequences of Staple oligomer or siRNA for TRPC6 gene. 31
Supplementary Method 32-37

a

Names	Control model sequences
2+2-63 nt	—AAAUAAAUGUCGACCUAGAUUAAUGCAAUU— 63nt —UUCCAGCACCCAAUUGAAGCUUUAAAUAAA—
2+2-100 nt	—AAAUAAAUGUCGACCUAGAUUAAUGCAAUU— 100nt—UUCCAGCACCCAAUUGAAGCUUUAAAUAAA—
2+2-140 nt	—AAAUAAAUGUCGACCUAGAUUAAUGCAAUU— 140nt—UUCCAGCACCCAAUUGAAGCUUUAAAUAAA—
3+1-63 nt	—AAAUAAAUAAAUGUCGACCUAGAUUAAUGCAAUU— 63nt —UUCCAGCACCCAAUUGAAGCUUUAAA—
3+1-100 nt	—AAAUAAAUAAAUGUCGACCUAGAUUAAUGCAAUU—100nt—UUCCAGCACCCAAUUGAAGCUUUAAA—
3+1-140 nt	—AAAUAAAUAAAAUGUCGACCUAGAUUAAUGCAAUU—140nt—UUCCAGCACCCAAUUGAAGCUUUAAA—

b

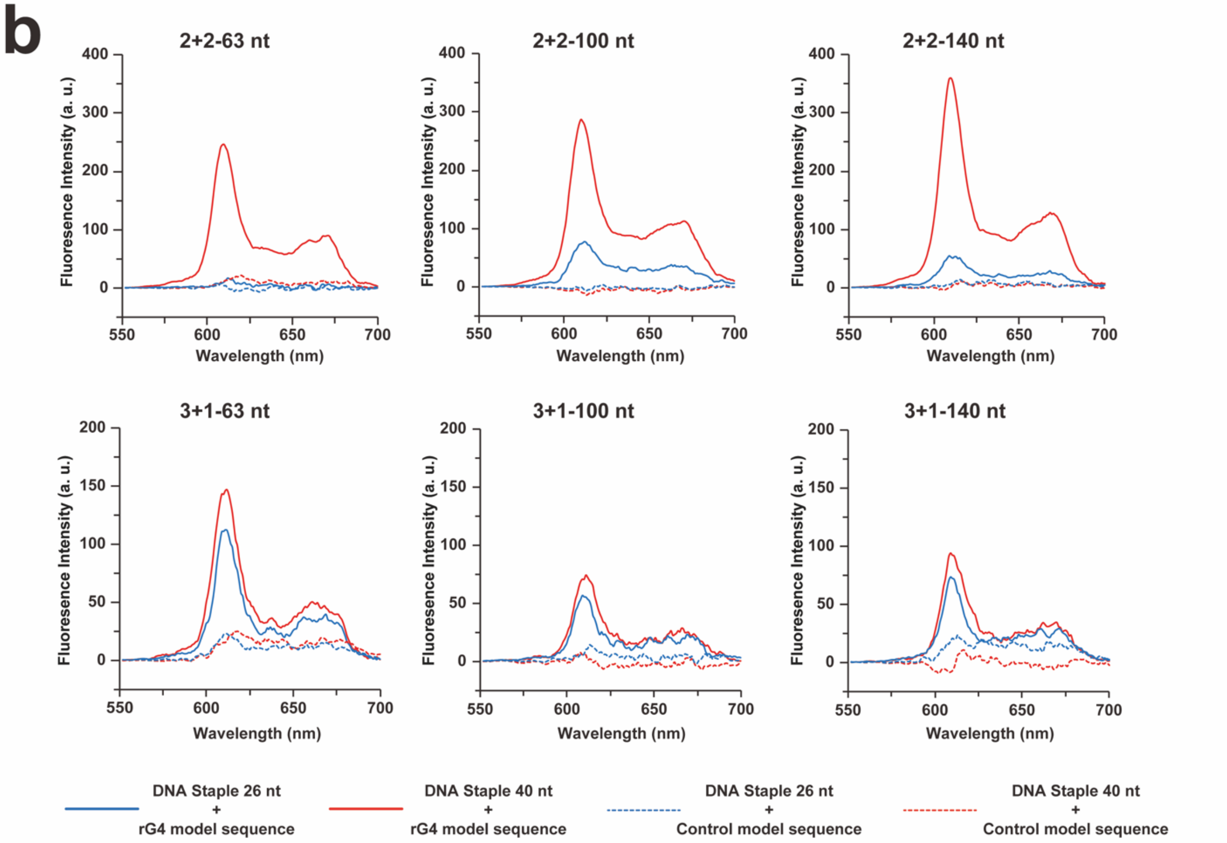

Supplementary Figure 1 | Evaluation of rG4 formation using NMM fluorescent probe. a, Nucleotide sequences of control models. The A-tracts are shown in blue. The dashed and solid underlines represent the 26-nt or 40-nt Staple oligomer recognition sites. **b,** The blue and red curves show the fluorescence emission spectra of NMM in the presence of 26-nt or 40-nt DNA Staple oligomers, respectively. The solid and dashed curves represent the model sequence with G-tracts and the control with A-tracts.

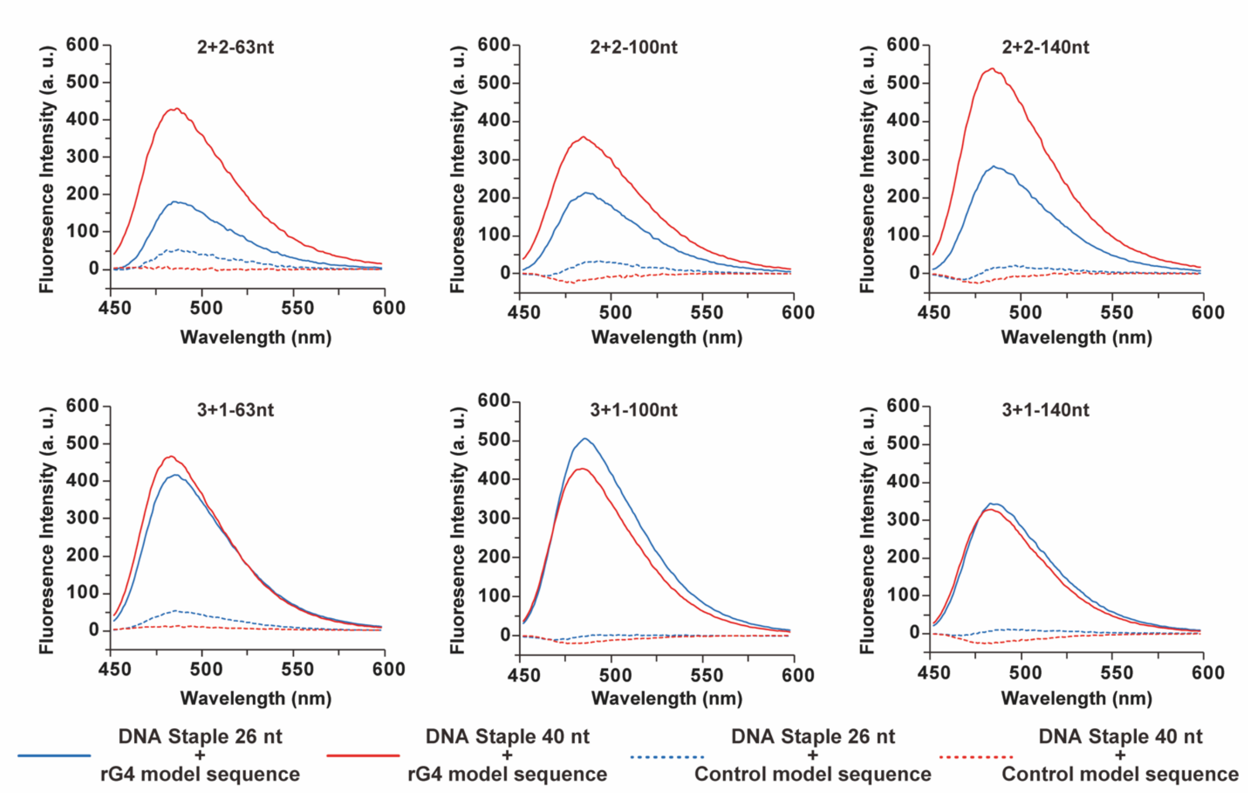

Supplementary Figure 2 | Evaluation of rG4 formation using ThT fluorescent probe. The blue and red curves show the fluorescence emission spectra of ThT in the presence of 26-nt or 40-nt DNA Staple oligomers, respectively. The solid and dashed curves represent the model sequence with G-tracts and the control with A-tracts.

a

Names	Sequences
rG4 template 1 (2+2 type)	GAAUUGCAUACGCUUGGGUGGG
rG4 template 2 (2+2 type)	GGGUGGGUAGCGUAUGCAAUUC
rG4 template 3 (3+1 type)	GAAUUGCAUACGCUUGGG
rG4 template 4 (3+1 type)	GGGUGGGUGGGUAGCGUAUGCAAUUC
rG4 template 5	GGGUGGGUGUCGACCUAGAUUC
rG4 template 6	GCCAAUUGAAGCUUUGGGUGGG
Control template 1	GAAUUGCAUACGCU
Control template 2	AGCGUAUGCAAUUC
Control template 3	GUCGACCUAGAUUC
Control template 4	GCCAAUUGAAGCUU
DNA Staple 1	GAATCTAGGTCGACAAGCTTCAATTGGC
DNA Staple 2	GAATCTAGGTCGACTTAAGCTTCAATTGGC
DNA Staple 3	GAATCTAGGTCGACTTTTAAGCTTCAATTGGC

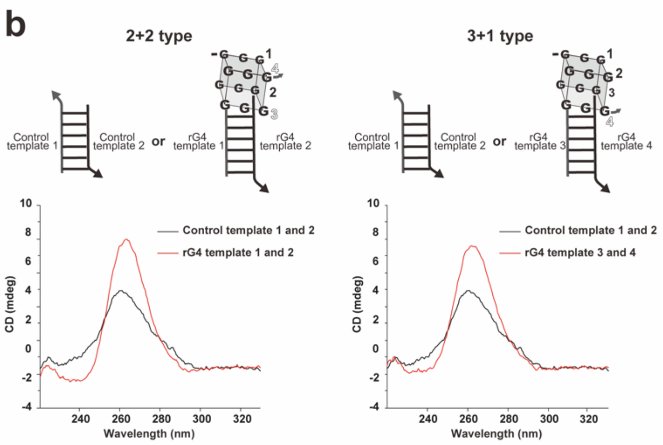

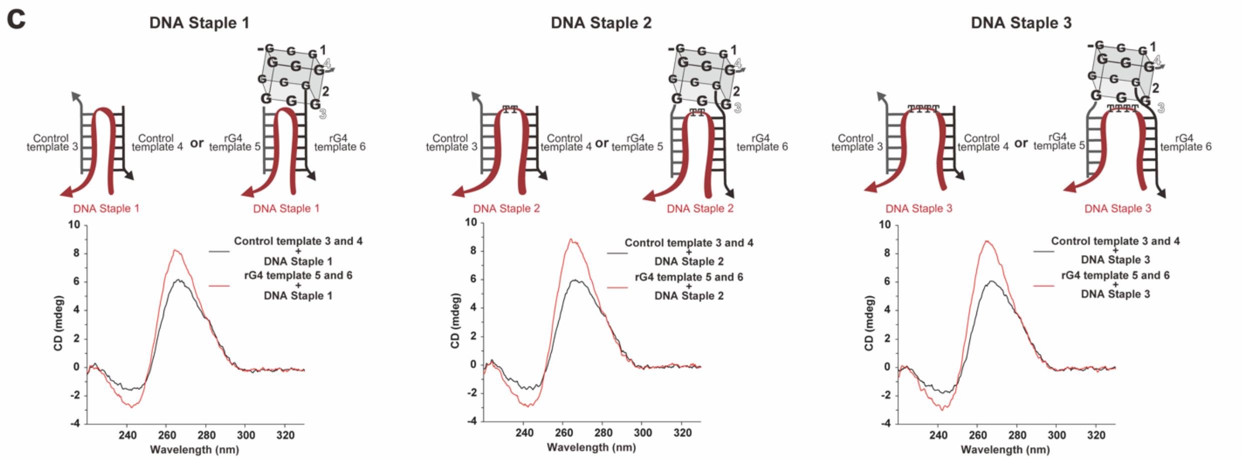

Supplementary Figure 3 | Evaluation of rG4 topology induced by Staple oligomers by Circular dichroism (CD) measurements. a, Nucleotide sequences of DNAs and RNAs. **b,** CD spectrum upon hybridization of two RNA strands. A positive Cotton effect around 260 nm and a negative Cotton effect around 240 nm were observed upon simple hybridization of the rG4 template 1 and 2. **c,** CD spectrum upon hybridization of two RNA strands with a DNA staple. The DNA Staple oligomer enhanced a positive Cotton effect around 260 nm and a negative Cotton effect around 240 nm.

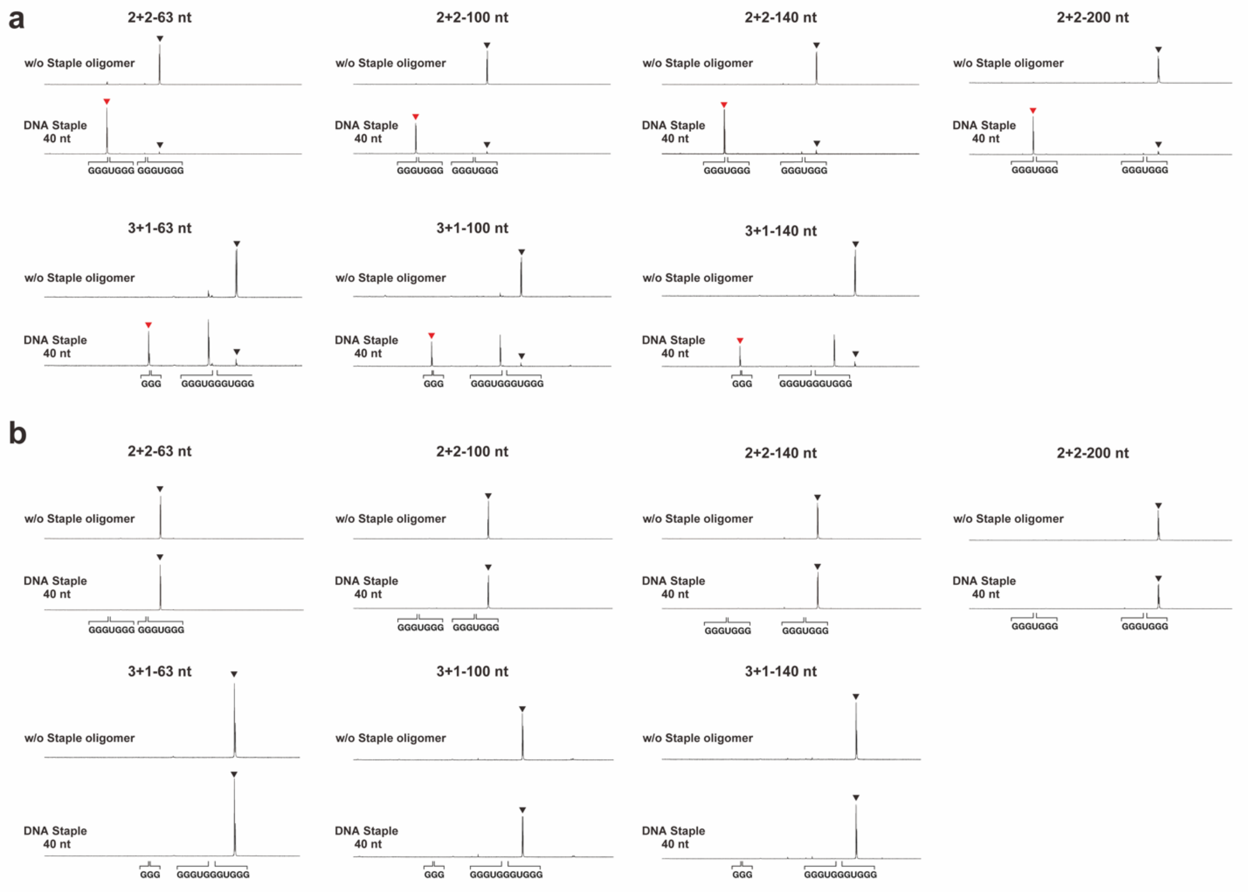

Supplementary Figure 4 | Identification of rG4 formation on the rG4 model sequences by RTase stop assay. a, Identification of sequencing spectra on rG4 model sequences in the presence of KCl. RTase-mediated cDNA synthesis was interrupted on all rG4 model sequences in the presence of 40-nt DNA Staple oligomer. Red and black arrowheads indicate the sites of arrest of RTase with rG4 induced by Staple oligomer and RTase elongation ends without rG4 induction, respectively. **b,** Identification of sequencing spectra on rG4 model sequences in the presence of LiCl. RTase-mediated cDNA synthesis in the presence of LiCl resulted in the elongation of cDNA sequences to their end. Black arrowheads indicate RTase elongation end without rG4 induction.

5' UTR of TRPC6 mRNA

CGCCUGUGCCCUCUGCCUGGGAGCCUGGGGCCGCCUGUCUGCGCGGUCCGGAUGCGCUCAGGUCAAGGUUCCUUUCGCGGCUGUCUC
CCAAGCCCCUAACUAGUGACUUCCACUGUGGCGGGCAGGGAAGCCAUUGGCAGAACCUAGCCAGUCAGGAAUCUGCAUCUCUUCCCU
CAUUAUCCUCUCCCUGGCAUUGCUUUGCUCGGGUCCUCCACGGAAGCAGGGUGCAGGCCGGCCAGGCACUGUGCCAUG

5' UTR of TRPC3 mRNA

GGGAAACCGCGCCGUCUCGCCGCGAUGCUGUCGGGCCGGCAGACCGCGCACAGCCGGCAGCGCGGCCGCGGACCCCAAGCUCCGCUG
CAGGUCGCGCGCUCCGCCCGGGCGUCCCCAGCUCCGCGGGCCCGGUGCUUGACCCGGGGCAGCUGGGCUGCUGACUGCGGGCGGCAG
GGAGCUUGGCCGCUAUG

Supplementary Figure 5 | Nucleotide sequences of 5′UTR of TRPC6 and TRPC3 mRNA. Guanine repeat sequences and start codon (AUG) are shown in red and orange, respectively. The dashed and solid underlines represent the 26-nt or 40-nt Staple oligomer recognition sites.

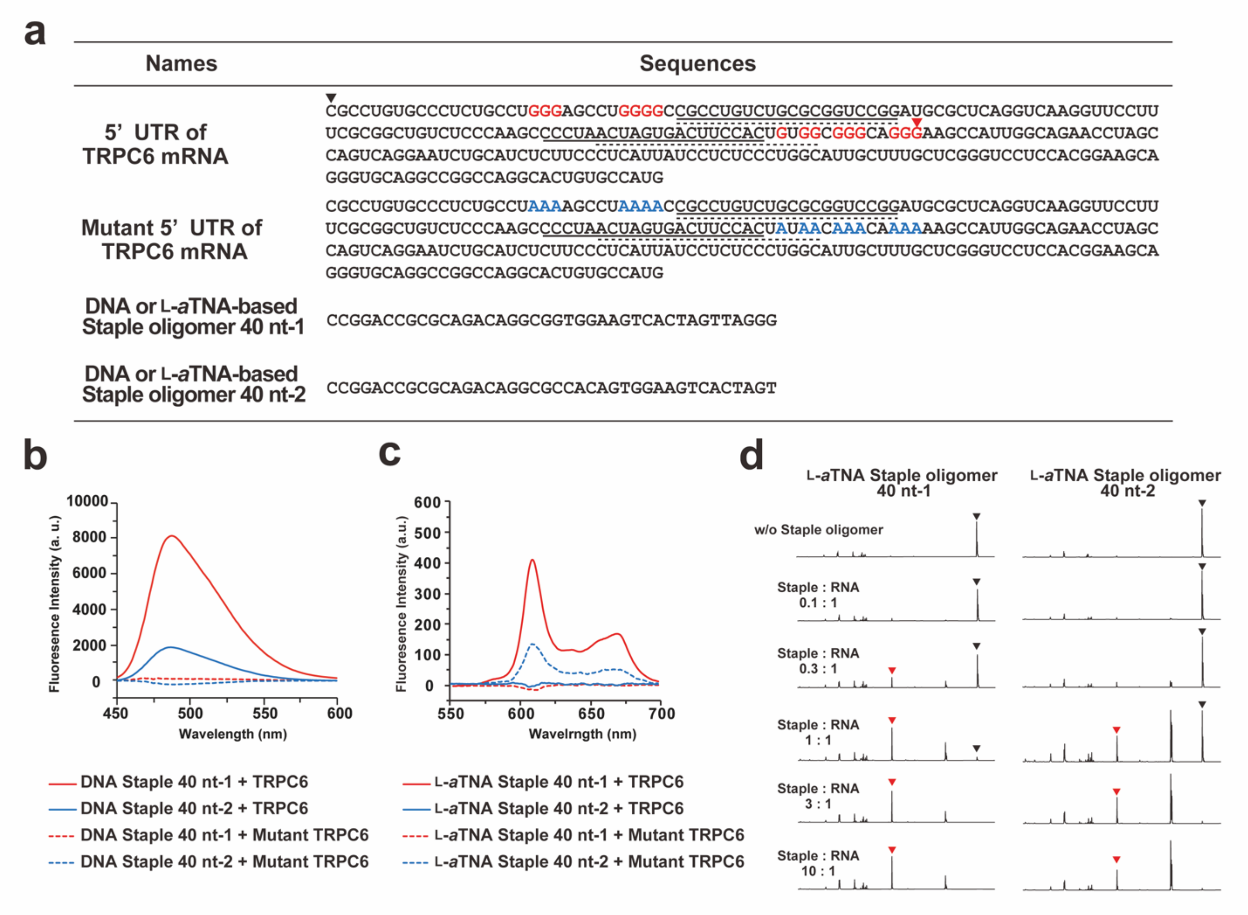

Supplementary Figure 6 | Determination of Staple oligomer target sequence for TRPC6 gene. a, Nucleotide sequences of the 5′UTR of TRPC6 mRNA, its synthetic control (mutant) and Staple oligomer. The target G-tracts are shown in red. The A-tracts, which replace the G-tracts, are shown in blue. The red and black arrowheads indicate the arrest site of RTase with rG4 induced by the Staple oligomer and the end of RTase elongation without rG4 induction, respectively. The dashed or solid underlines represent the recognition sites of L-*a*TNA-based Staple oligomer 40nt-1 or 40nt-2, respectively. **b,** Evaluation of rG4 formation using ThT fluorescent probe. The red and blue curves show the fluorescence emission spectra of ThT in the presence of DNA Staple-1 or DNA Staple-2, respectively. The solid and dashed curves represent the model sequence with G-tracts and the control with A-tracts. **c,** Evaluation of rG4 formation using NMM fluorescent probe. The curves show the fluorescence emission spectra of NMM in the presence of the L-*a*TNA-based Staple oligomer 40nt-1 (left panel) and 40nt-2 (right panel). The solid and dashed curves represent the fluorescent spectra for 5′UTR of TRPC6 mRNA and its mutant with the Staple oligomer. **d,** Identification of rG4 formation with the L-*a*TNA-based Staple oligomer 40nt-1 or 40nt-2 on the TRPC6 sequence by RTase stop assay. Red and black arrowheads indicate the arrest site of RTase with rG4 induced by the L-*a*TNA-based Staple oligomers and the RTase elongation end without rG4 induction, respectively.

.

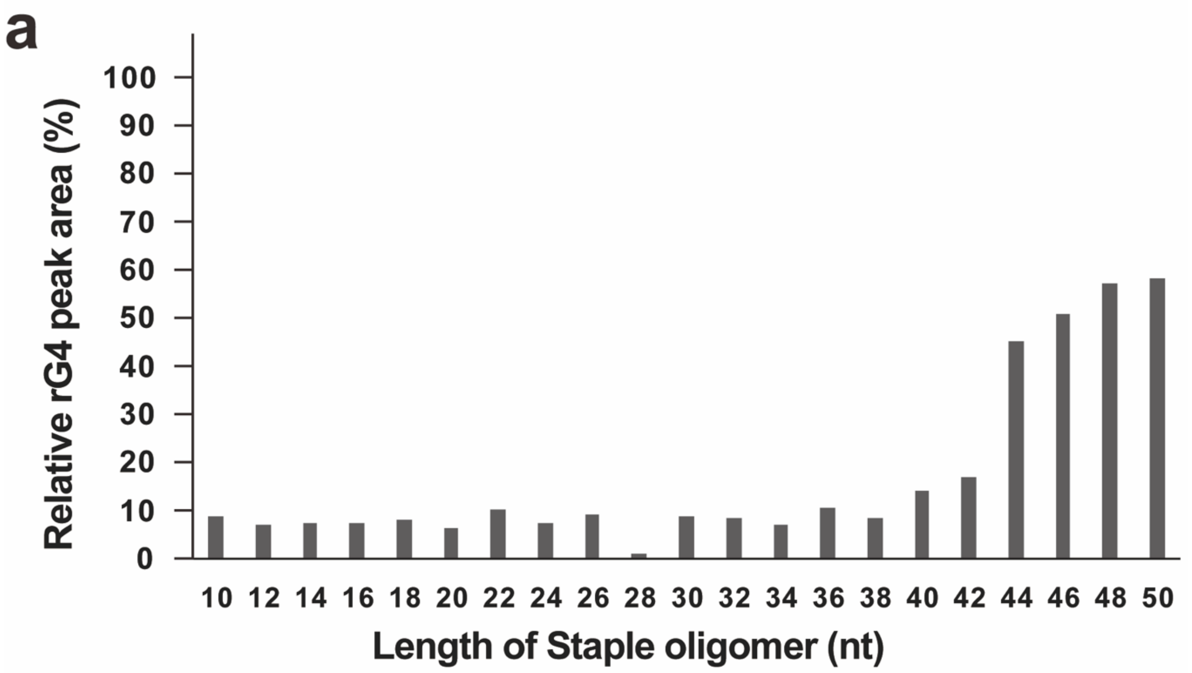

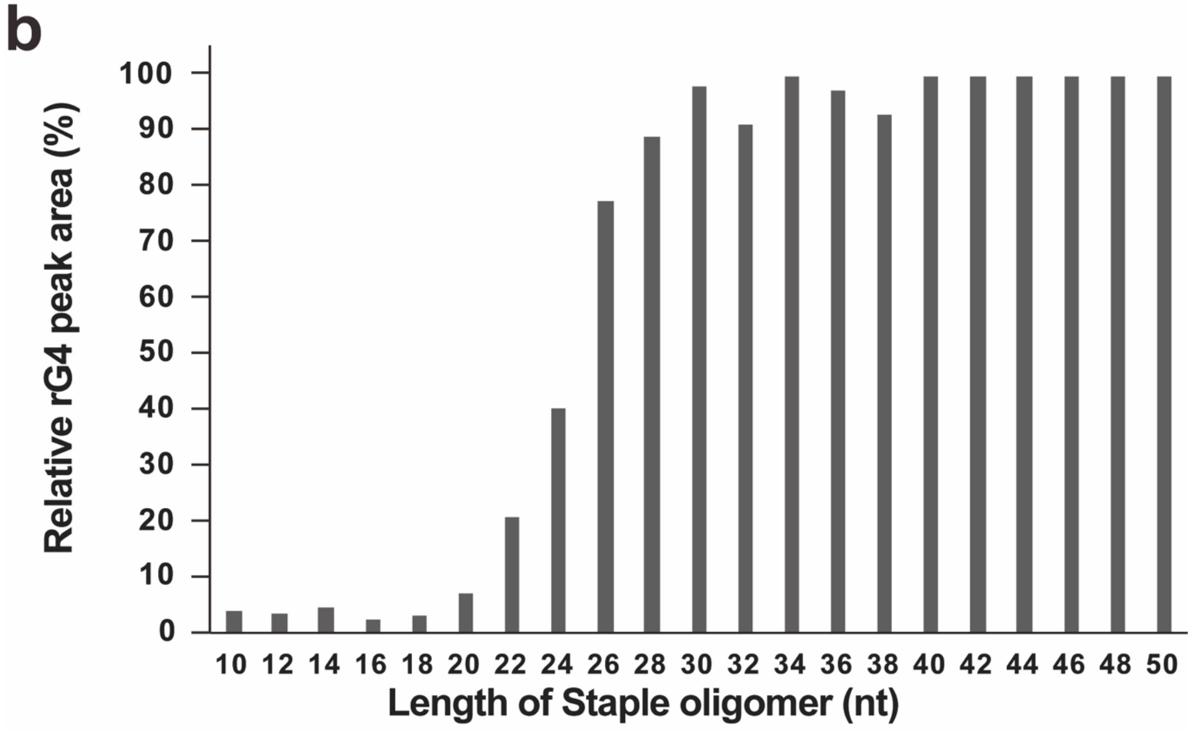

Supplementary Figure 7 | rG4 induction with RNAh technology in various Staple oligomers. a, Length optimization of DNA Staple. The 44-nt DNA Staple induced approximately 50% rG4 structure formation in the target sequence of TRPC6 mRNA. **b,** Length optimization of RNA Staple. The 26-nt RNA Staple induced approximately 80% rG4 formation in the target sequence of TRPC6 mRNA. Here, 44 nt for DNA and 26 nt for RNA were the minimum lengths to maintain effective Staple activity in the TPCR6 mRNA.

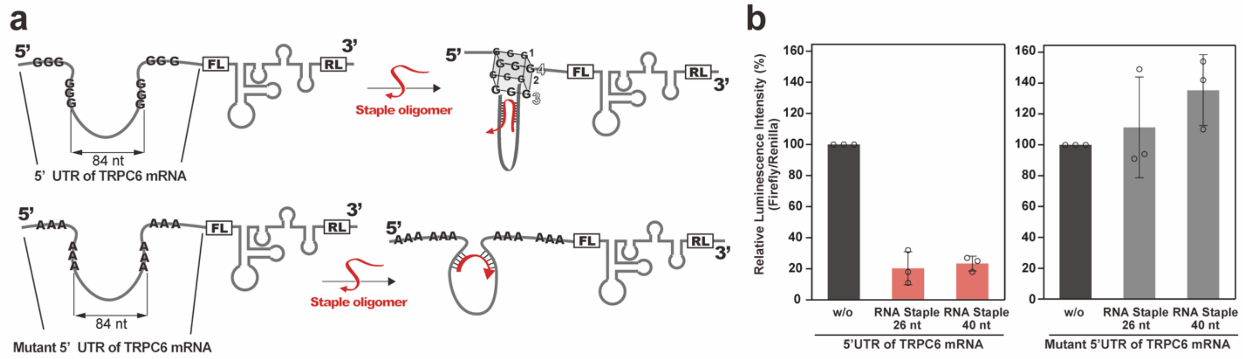

c

Names	Sequences
5' UTR of TPM3 mRNA	GGUCACAUCCGGGCGGGUUGGUGAGUUCCGGUAUUUCAGGGCGUAGCAGGCGGAAGUAAGGGUGAGAGGAGGCUGCAACGCCGAGCGGAGGAGGCAGGAACCGGAGCGCGAGCAGUAGCUGGGUGGGCACCCAGGUAUACAUAGUUGGAAAUCUCUGGAAGAUCCGCGCGUACCGAGUUCUAAUUCACUGGCCGUCGUUUUA
5' UTR of MYD88 mRNA	GAGAUUCCUACUUCUUACGCCCCCCACAUCACCCGCCUCGAGACCUCAAGGGUAGAGGUGGGCACCCCCGCCUCCGCACUUUUGCUCGGGGCUCCAGAUUGUAGGGCAGGGCGGCGCUUCUCGGAAAGCGAAAGCCGGCGGGGCGGGGCGGGUGCCGCAGGAGAAAGAGGAAGCGCUGGCAGACACAGGUAUACAUAGUUGG
DNA Staple oligomer (TPM3)	TGAAATACCGGAACTCACCAGCTACTGCTCGCGCTCCGGT
DNA Staple oligomer (MYD88)	CTTCCTCTTTCTCCTGCGGCACAATCTGGAGCCCCGAGCA

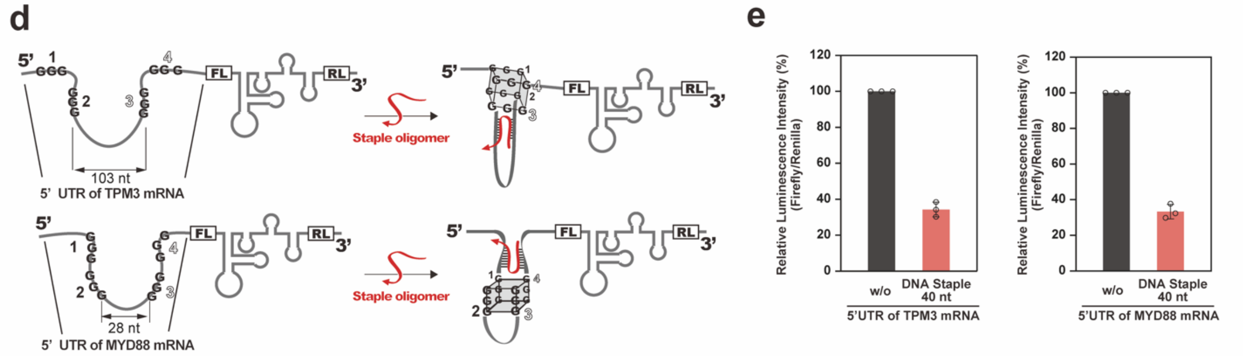

Supplementary Figure 8 | Validation of the versatility of RNAh technology with Staple oligomers using in vitro translation assay. a, The mRNAs encoding firefly luciferase (FL) with 5′UTR of TRPC6 (upper scheme) and its mutant (lower scheme) are illustrated. Renilla luciferase (RL) was placed downstream of IRES as an internal expression control. **b,** Translation efficiency was demonstrated as the relative luciferase activity (Firefly/Renilla) for each mRNA with RNA Staple oligomers. Data were normalized by the relative luciferase activity without Staple oligomer. FL expression was strongly suppressed by Staple oligomers only in the 5′UTR of TRPC6, but not in its mutant. Data are mean ± S.D. of three independent experiments. **c,** Nucleotide sequences of the 5′UTR of TPM3 and MYD88 mRNAs and DNA Staple oligomers. The G-tracts are shown in red. Underline shows a hybridization site for the DNA Staple oligomer. **d,** Illustration of mRNAs encoding FL with 5′UTRs of human cancer-related genes (TPM3, upper scheme; MYD88, lower scheme). **e,** Translation efficiency in each mRNA with the 5′UTR of TPM3 or MYD88 was demonstrated as the relative luciferase activity (Firefly/Renilla) for each mRNA with or without the 40-nt DNA Staple oligomers. Data were normalized by relative luciferase activity without Staple oligomer. The RNA Staple oligomers effectively suppressed in vitro translation of their target mRNAs. Data are mean ± S.D. of three independent experiments.

a

Names	Sequences
CDS of Nano luciferase mRNA	AUGGUCUUCACACUCGAAGAUUUCGUUGGGGACUGGCGACAGACAGCCGGCUACAACCUGGACCAAGUCCUUGAACAGGGAGGGGUGUCCA GUUUGUUUCAGAAUCUCGGGGUGUCCGUAACUCCGAUCCAAAGGAUUGUCCUGAGCGGGGAAAAUGGGCUGAAGAUCGACAUCCAUGUCAU CAUCCCGUAUGAAGGUCUGAGCGGGGACCAAAUGGGCCAGAUCGAAAAAAUUUUUAAGGUGGUGUACCCUGUGGAUGAUCAUCACUUUAAG GUGAUCCUGCACUAUGGCACACUGGUAAUCGACGGGGUUACGCCGAACAUGAUCGACUAUUUCGGACGGCCGUAUGAAGGCAUCGCCGUGU UCGACGGCAAAAAGAUCACUGUAACAGGGACCCUGUGGAACGGCAACAAAAUUAUCGACGAGCGCCUGAUCAACCCCGACGGCUCCCUGCU GUUCCGAGUAACCAUCAACGGAGUGACCGGCUGGCGGCUGUGCGAACGCAUUCUGGCGUAA
DNA Staple oligomer	GACATGGATGTCGATCTTCAGTTCAAGGACTTGGTCCAGG
Non-targeting DNA oligomer	CCGGACCGCGCAGACAGGCGGTGGAAGTCACTAGTTAGGG

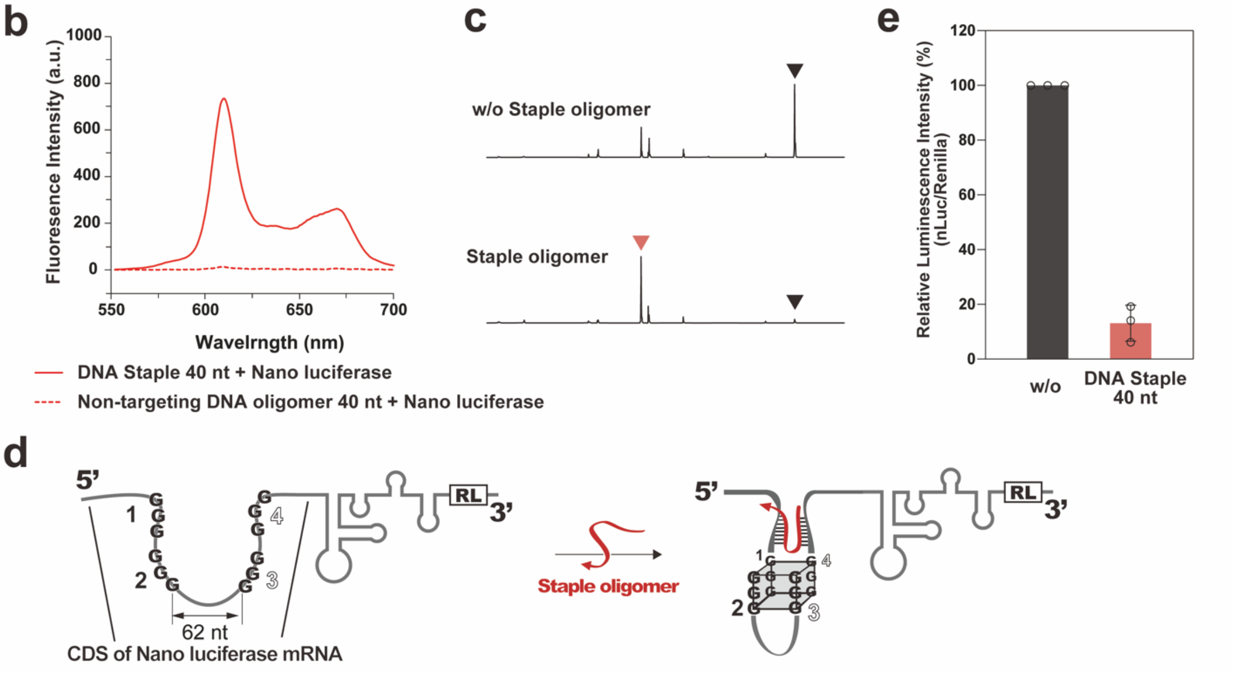

Supplementary Figure 9 | Characterization of RNAh technology to Nano luciferase CDS region. a, Nucleotide sequences of the cording sequence (CDS) of Nano luciferase (nLuc) mRNA that was forcibly mutated to possess four G-tracts, a DNA Staple oligomer, and a non-targeting control DNA oligomer. The G-tracts are shown in red. Red and black arrowheads indicate the arrest site of RTase with rG4 induced by the Staple oligomer and the end of RTase elongation without rG4 induction, respectively. The solid underlines represent the recognition sites by Staple oligomers. **b,** Evaluation of rG4 formation using NMM fluorescent probe. The solid and dashed curves represent the fluorescence emission spectra of NMM with nLuc mRNA in the presence of the Staple oligomer and non-targeting control DNA oligomer, respectively. **c,** Identification of rG4 formation with the Staple oligomers on the nLuc sequence by RTase stop assay. Red arrowhead and black arrowhead indicate the arrest site of RTase with rG4 induced by the Staple oligomers and the RTase elongation end without rG4 induction, respectively. **d,** Dual reporter genes encoding nLuc and Renilla luciferase (RL) in tandem were used to validate the mechanism of action of RNAh. RL was placed downstream of an IRES as an internal standard. rG4 formation is induced only in the presence of Staple oligomers. **e,** Translation efficiency was demonstrated as the relative luciferase activity (nLuc/Renilla luciferase) for nLuc mRNA with or without the Staple oligomers. The nLuc expression was strongly suppressed by Staple oligomers. Data are mean ± S.D. of three independent experiments.

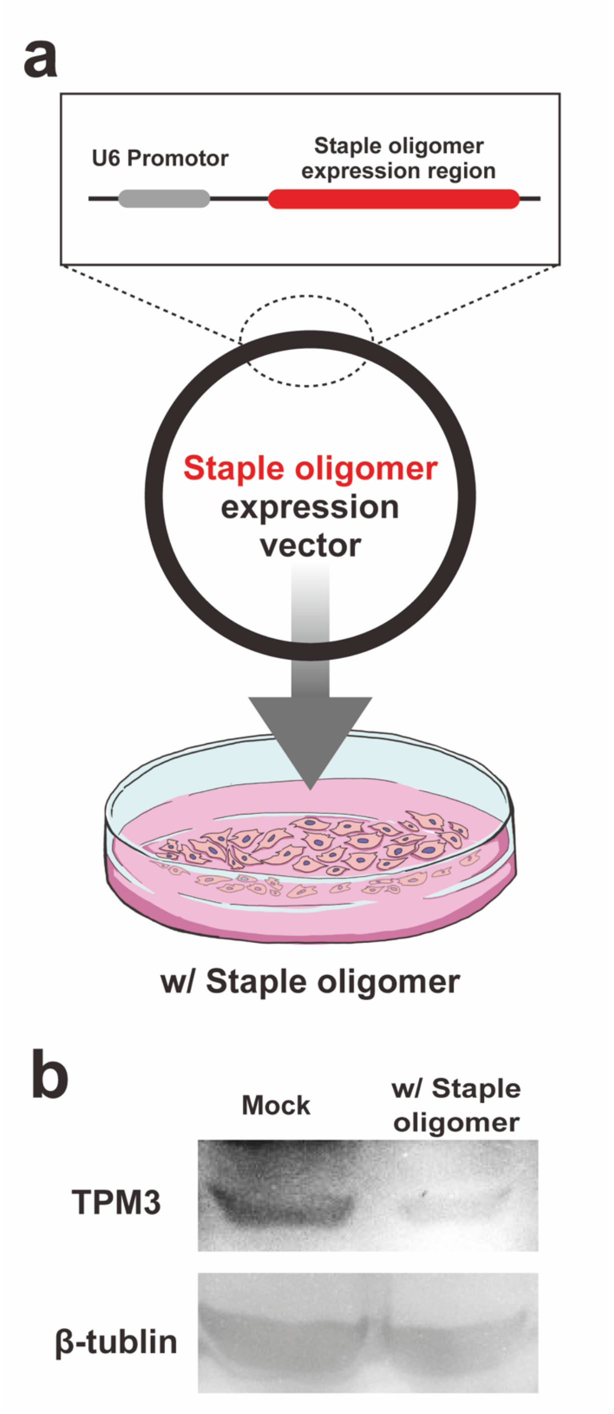

Supplementary Figure 10 | Effect of RNAh technology on target protein expression in mammalian living cells. a, Design and construction of an expression vector encoding Staple oligomer. The short hairpin RNA expression vector was used to express an RNA Staple oligomer. U6 promoter and Staple oligomer are shown in gray and red, respectively. **b,** Evaluation of the effect of 26-nt RNA Staple oligomers on TPM3 expression in MCF7 cells by western blotting. RNA Staple oligomers effectively suppressed TPM3 gene expression.

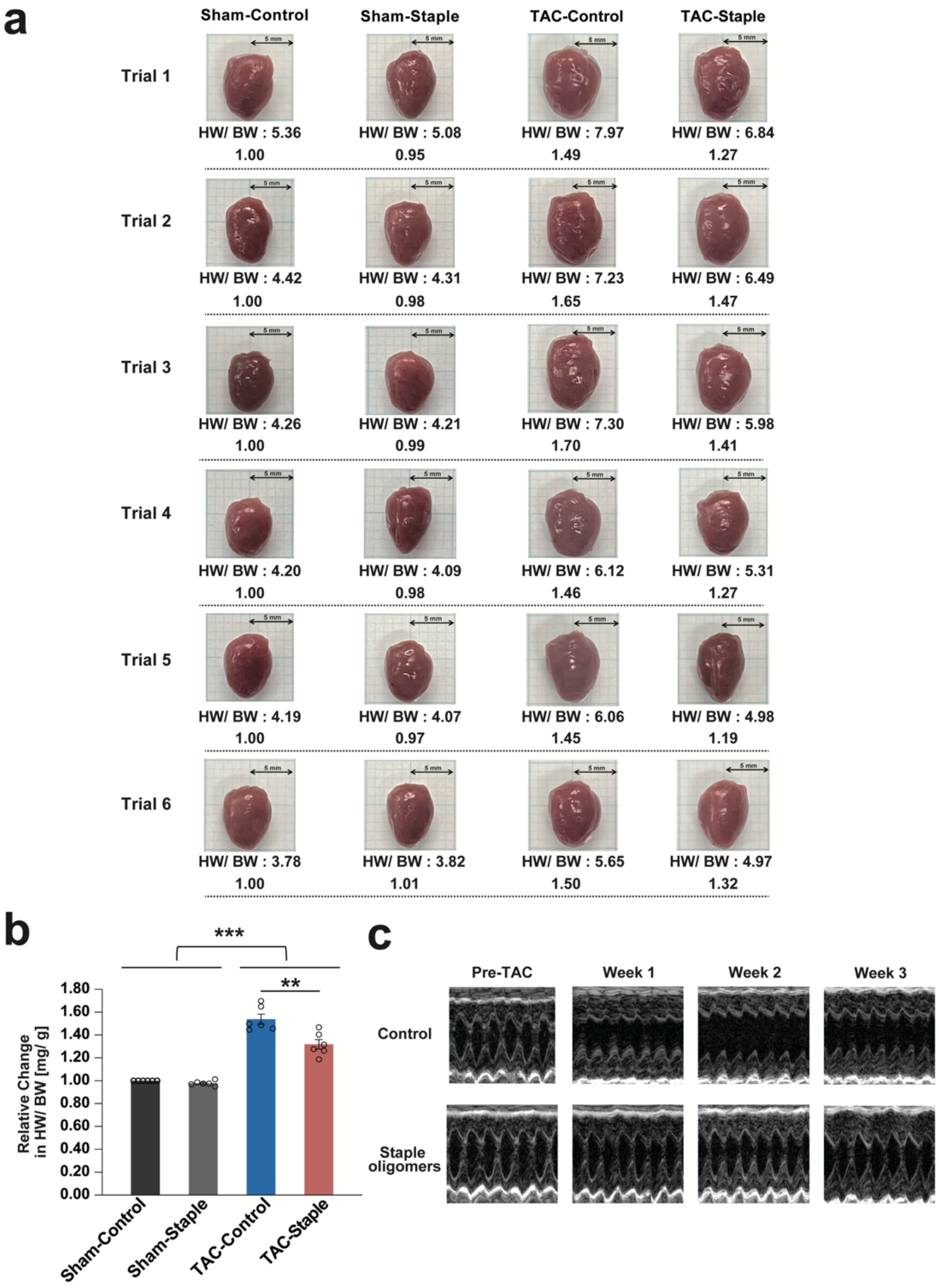

Supplementary Figure 11 | Effects of Staple oligomers on myocardial hypertrophy in the TAC-treated mouse hearts. a, Representative images of hearts from Sham and TAC-treated mice infected with control-AAV6 or Staple-oligomer-AAV6. Myocardial enlargement of TAC-treated mice was significantly suppressed by the RNA Staple oligomer. **b,** The hypertrophic effect of myocardium on heart weight in the TAC-treated and -untreated groups and in the Staple-oligomer-treated and -untreated groups. HW/BW represents heart weight/body weight. Whereas the heart weight was increased by TAC treatment (TAC-Control, blue bar), this was significantly suppressed by the expression of Staple oligomers (TAC-Staple, red bar). Data are shown as mean ± S.E. (n=6 mice/group, biologically independent samples). Statistical significance was determined two-way ANOVA (two-sided, ***P<0.001, ** P=0.0040). **c,** Echocardiography showed no significant decrease in cardiac function after TAC treatment in the Staple-oligomer-treated mice (lower panel), but a marked decrease in FS values in the Staple-oligomer-untreated mice (upper panel).

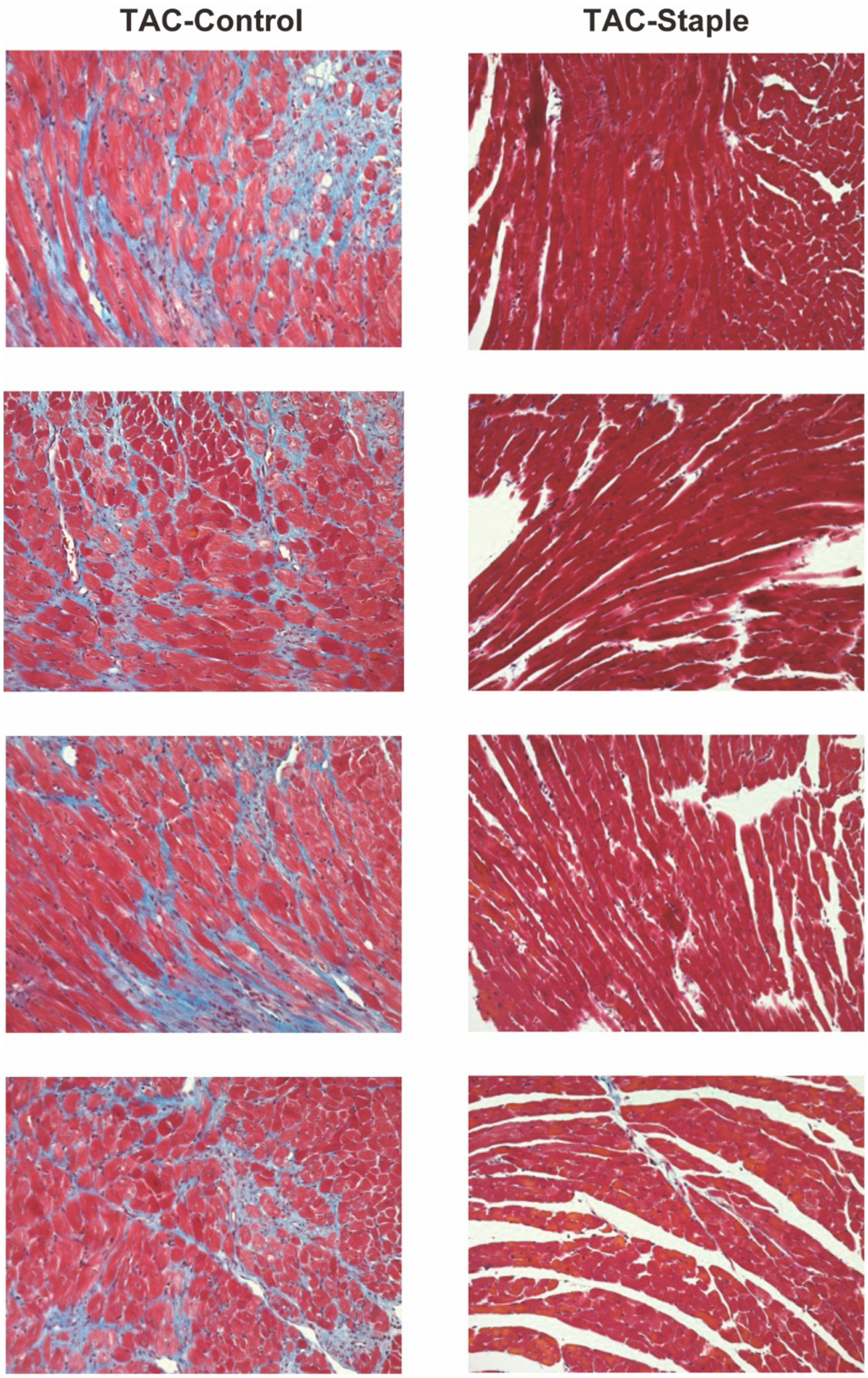

Supplementary Figure 12 | Effects of Staple oligomers on myocardial hypertrophy by transverse aortic constriction (TAC). Evaluation of myocardial fibrosis by Masson's staining (blue area: area of fibrosis in the heart) showed that the Staple-oligomer-treated myocardium was significantly less fibrotic.

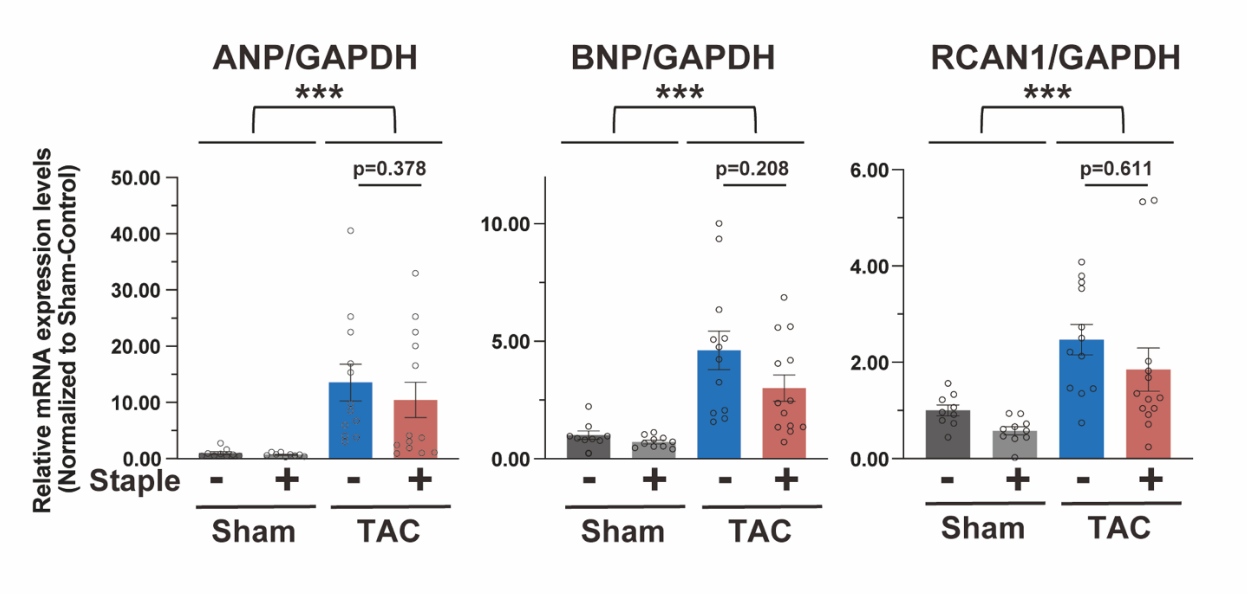

Supplementary Figure 13 | Effects of Staple oligomers on gene expression in the TAC-treated mouse hearts. Evaluation of mRNA expression levels of ANP, BNP, and RCAN1, known markers of cardiac hypertrophy, by qPCR. The mRNA expression levels were increased by TAC treatment (blue bars), but the presence of Staple oligomers suppressed the increase in their mRNA expression levels (red bars). Data are shown as mean ± S.E. (n=9 mice/Sham group, n=12 mice/TAC Control, or n=13 mice/TAC Staple, biologically independent samples). Statistical significance was determined by two-way ANOVA (two-sided, ***P<0.001).

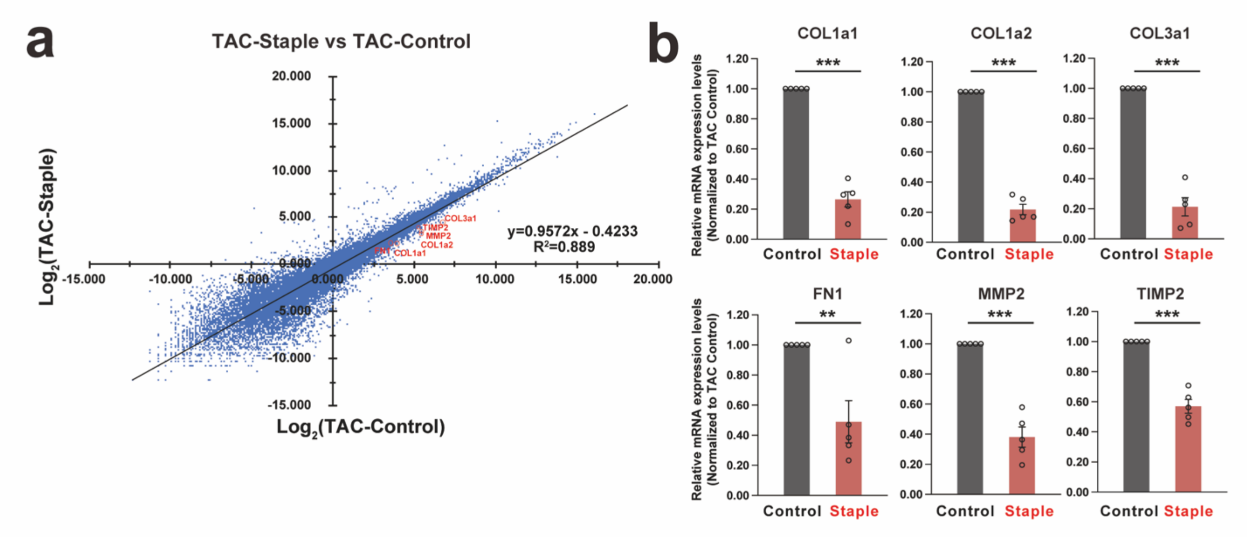

Supplementary Figure 14 | mRNA-seq and proteomic analysis on myocardial hypertrophy. a, Scatter plot analysis of mRNA-seq from TAC-Control and TAC-Staple. **b,** The expression levels of cardiac fibrosis-related genes were significantly reduced with Staple oligomer treatments in TAC-treated mice. Data are shown as mean ± S.E. (n=5 mice/group, biologically independent samples). Statistical significance was determined by Student's t-tests (***P<0.001, **P= 0.00659).

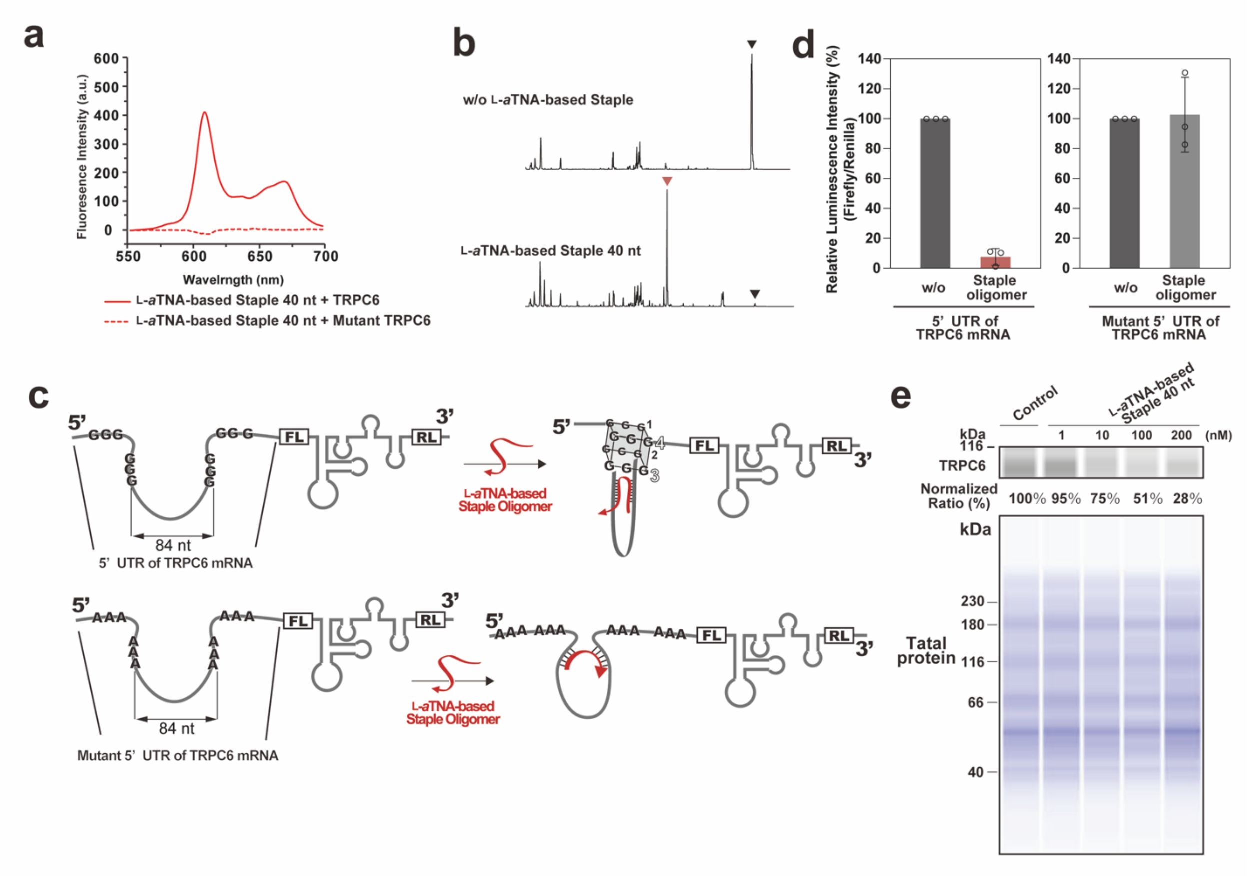

Supplementary Figure 15 | Characterization of RNAh technology with L-*a*TNA-based Staple oligomer. a, Evaluation of rG4 formation using NMM fluorescent probe. The curves show the fluorescence emission spectra of NMM in the presence of a 40-nt L-*a*TNA-based Staple oligomer. The solid and dashed curves represent the 5′UTR of TRPC6 and its mutant, respectively. **b,** Identification of rG4 formation with the 40-nt L-*a*TNA-based Staple oligomers on the TRPC6 sequence by RTase stop assay. Red arrowhead indicates the site of arrest of RTase with rG4 induced by the L-*a*TNA-based Staple oligomers, and black arrowheads indicate the RTase elongation end without rG4 induction. **c,** The mRNAs encoding firefly luciferase (FL) with the 5′ UTR of TRPC6 (upper scheme) and its mutant (lower scheme) are illustrated. Renilla luciferase (RL) was placed downstream of IRES as an internal expression control. The L-*a*TNA-based Staple oligomer hybridizes to the 5′ UTRs and induces rG4 formation only in native ones. **d,** Translation efficiency in each mRNA with the 5′UTR of TRPC6 or Mutant 5′UTR of TRPC6 was demonstrated as the relative luciferase activity (Firefly/Renilla) for each mRNA with or without the L-*a*TNA-based Staple oligomers. Data were normalized by relative luciferase activity without L-*a*TNA-based Staple oligomer. FL expression was strongly suppressed by L-*a*TNA-based Staple oligomers only in the 5′ UTR of TRPC6 (left panel), but not in its mutant (right panel). Data are mean ± S.D. of three independent experiments. **e,** Evaluation of the effects of the L-*a*TNA-based Staple oligomer on TRPC6 expression in C2C12 cells by western blotting. TRPC6 expression was suppressed by the L-*a*TNA-based Staple oligomers in a dose-dependent manner (upper panel). TRPC6 signals were normalized to total protein (lower panel).

a

Names	Sequences
L-*a*TNA-based Staple oligomer 40 nt	Cy5-CCGGACCGCGCAGACAGGCGGTGGAAGTCACTAGTTAGGG-FITC
RNA Staple oligomer 40 nt	Cy5-CCGGACCGCGCAGACAGGCGGUGGAAGUCACUAGUUAGGG-FITC

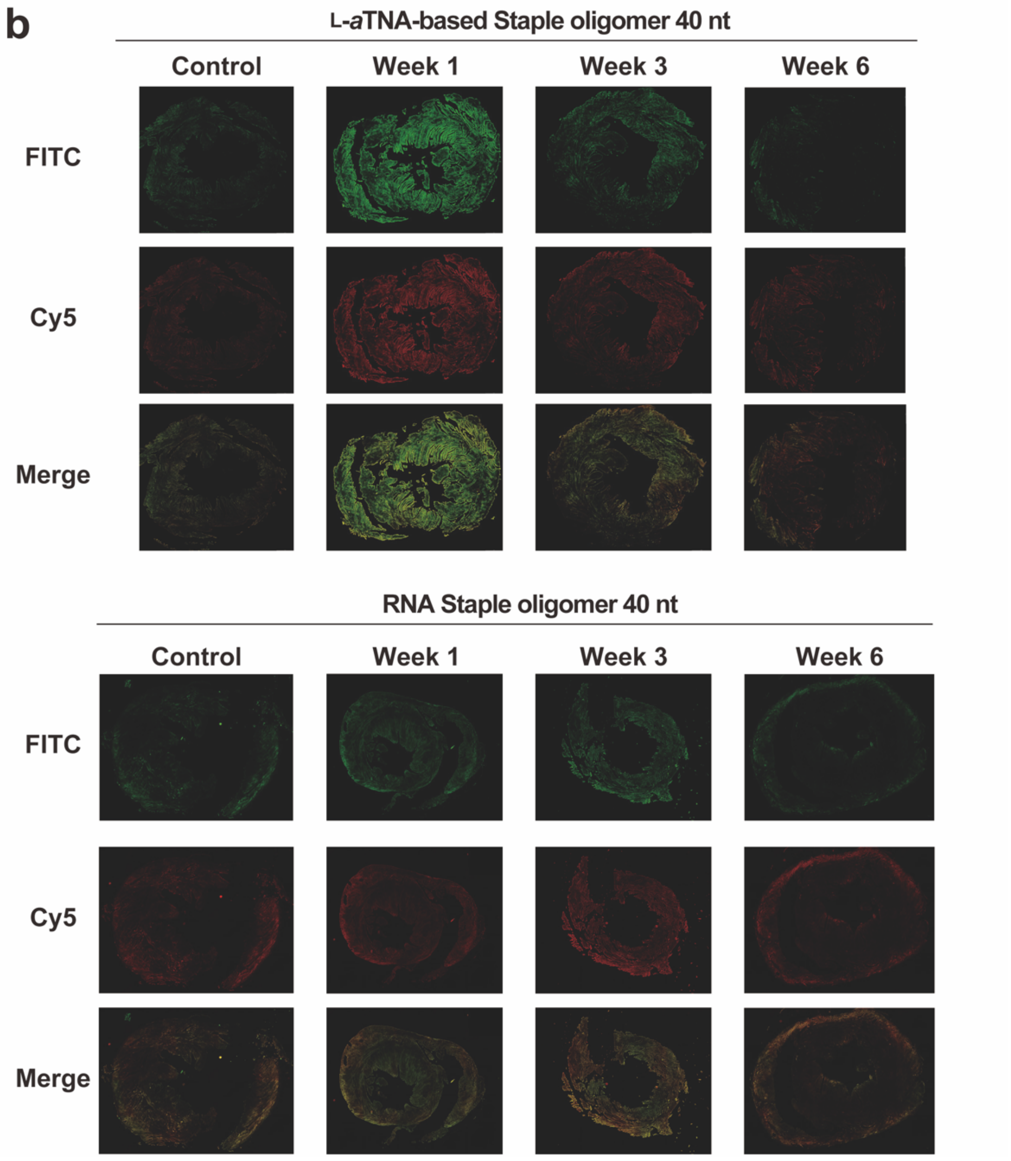

Supplementary Figure 16 | Cardiac delivery of L-*a*TNA-based or RNA Staple oligomer. a, Base sequence of 5′-Cy5 and 3′-FITC double-labeled L-*a*TNA-based and RNA Staple oligomer. **b,** Heart sections of mice treated with the double-fluorescently labeled L-*a*TNA-based and RNA Staple oligomer. Fluorescent signals of FITC (top) and Cy5 (middle) in heart sections at weeks 1, 3, and 6 after transfection of the double-fluorescently labeled L-*a*TNA-based or RNA Staple oligomers into mice. Merged panel shows that fluorescent signals from FITC and Cy5 well overlapped up to week 6, indicating that L-*a*TNA-based Staple oligomers were stable in the heart for 1 week and gradually eliminated without detectable degradation up to week 6. Conversely, fluorescence co-localization derived from RNA Staple oligomers was disrupted within 1 week.

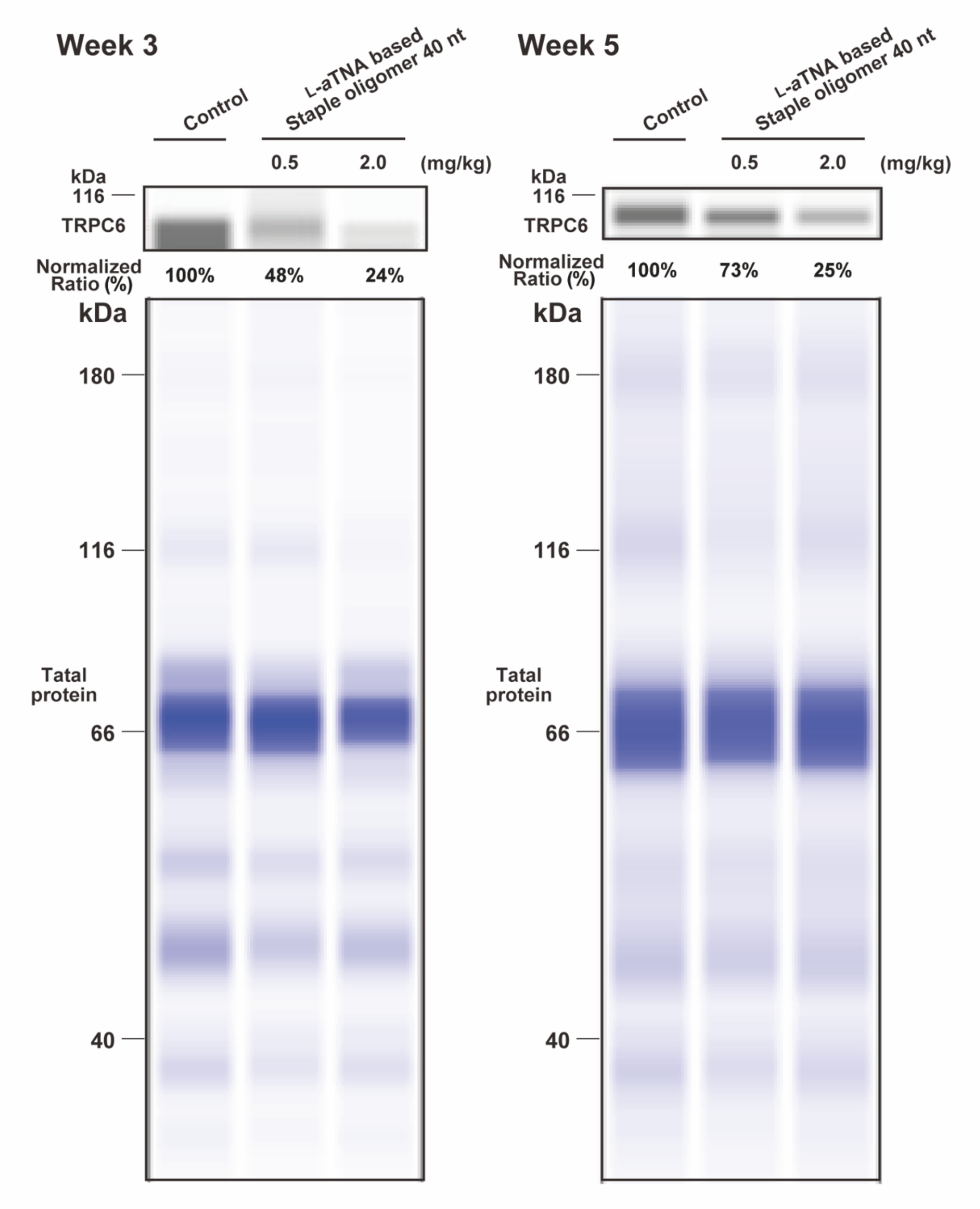

Supplementary Figure 17 | Effects of L-*a*TNA-based Staple oligomer on TRPC6 expression in mouse hearts. Western blot analysis revealed that mice treated with L-*a*TNA-based staple oligomers showed reduced expression of TRPC6 at weeks 3 and 5 after treatment. A dose-dependent decrease in protein expression was observed at concentrations of 0.5 and 2.0 mg/kg (upper panel). TRPC6 signals were normalized to total protein (lower panel).

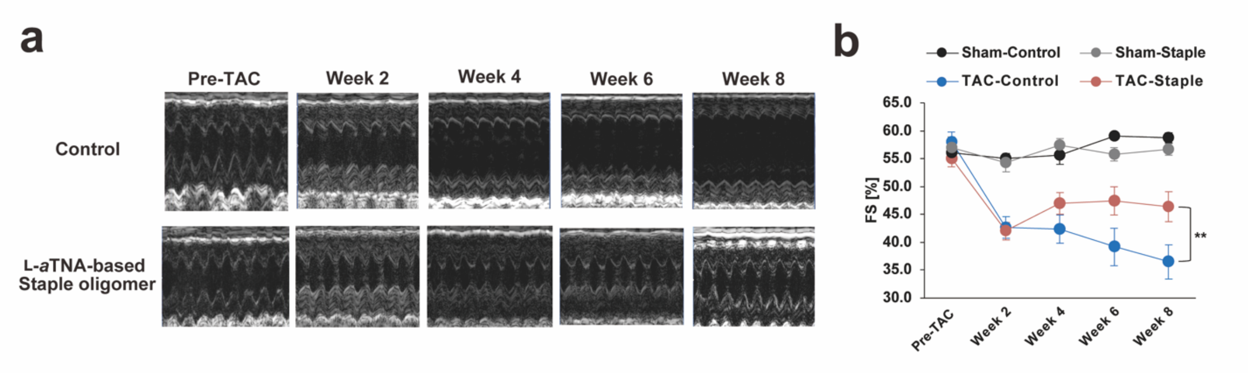

Supplementary Figure 18 | Effects of L-*a*TNA-based Staple oligomer on cardiac function in TAC-treated mouse hearts. a, Echocardiography showed no significant decrease in cardiac function after TAC treatment in mice treated with 2.0 mg/kg L-*a*TNA-based Staple oligomer (lower panel), but a marked decrease in FS values in Staple-oligomer-untreated mice (upper panel) up to 8 weeks. **b,** Echocardiographic evaluation (FS values) revealed that cardiac function was maintained in the presence of 2.0 mg/kg L-*a*TNA-based Staple oligomers. Data are shown as mean ± S.E. (n=8 mice/Sham group, n=9 mice/TAC Control, or n=13 mice/TAC Staple, biologically independent samples). Statistical significance was determined by two-tailed ANCOVA using number of weeks as a covariate (two-sided without adjustment): **P=0.0041, compared with mock-transfected mice.

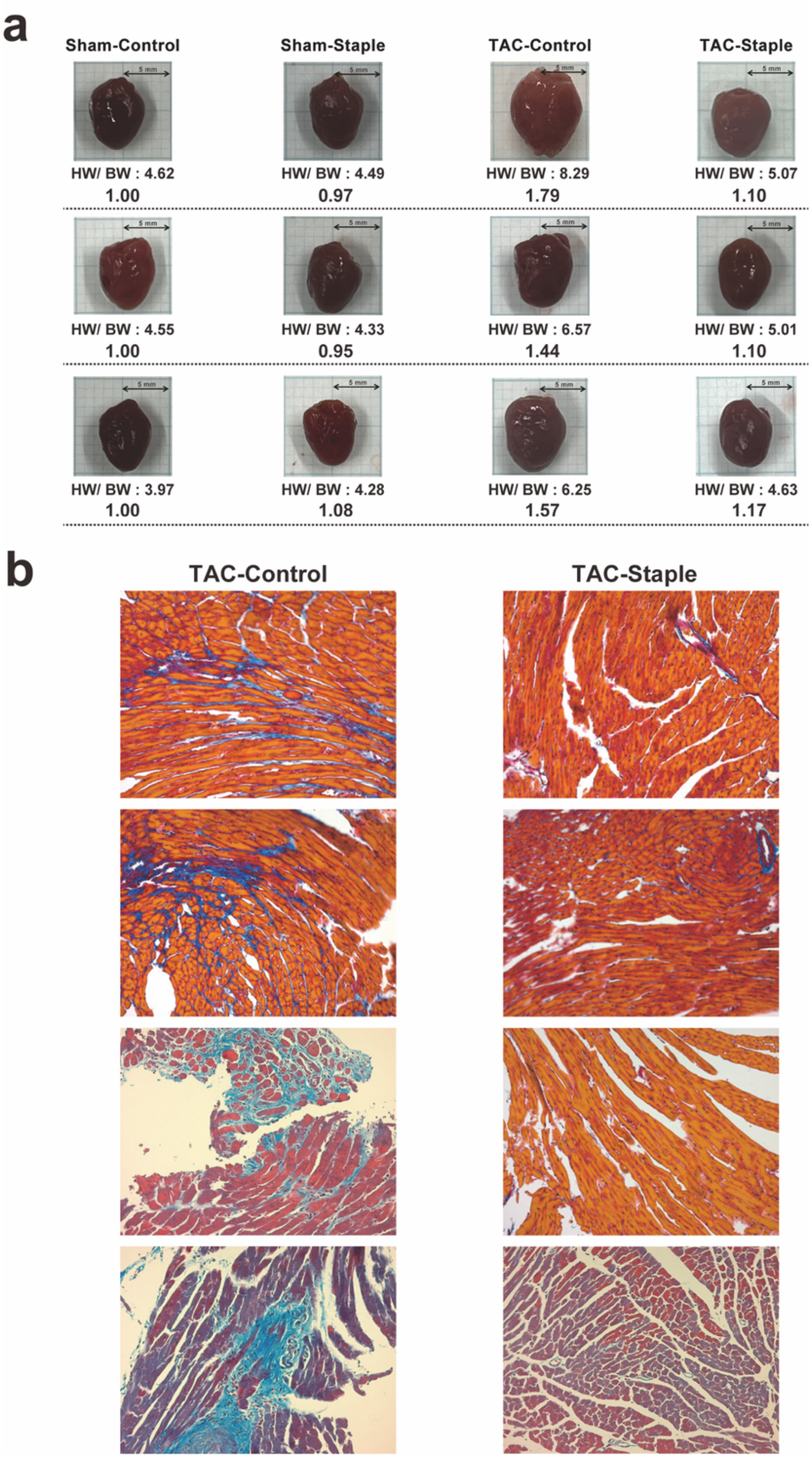

Supplementary Figure 19 | Effects of L-*a*TNA-based Staple oligomers on myocardial hypertrophy in TAC-treated mouse hearts. a, Representative images of hearts from Sham and TAC-treated mice transfected with Mock or 2.0 mg/kg L-*a*TNA-based Staple oligomer, respectively. Myocardial enlargement in TAC-treated mice was significantly suppressed by the L-*a*TNA-based Staple oligomers. **b,** Evaluation of myocardial fibrosis by Masson's staining (blue area: area of fibrosis in the heart) showed that myocardium treated with 2.0 mg/kg L-*a*TNA-based Staple oligomer was significantly less fibrotic.

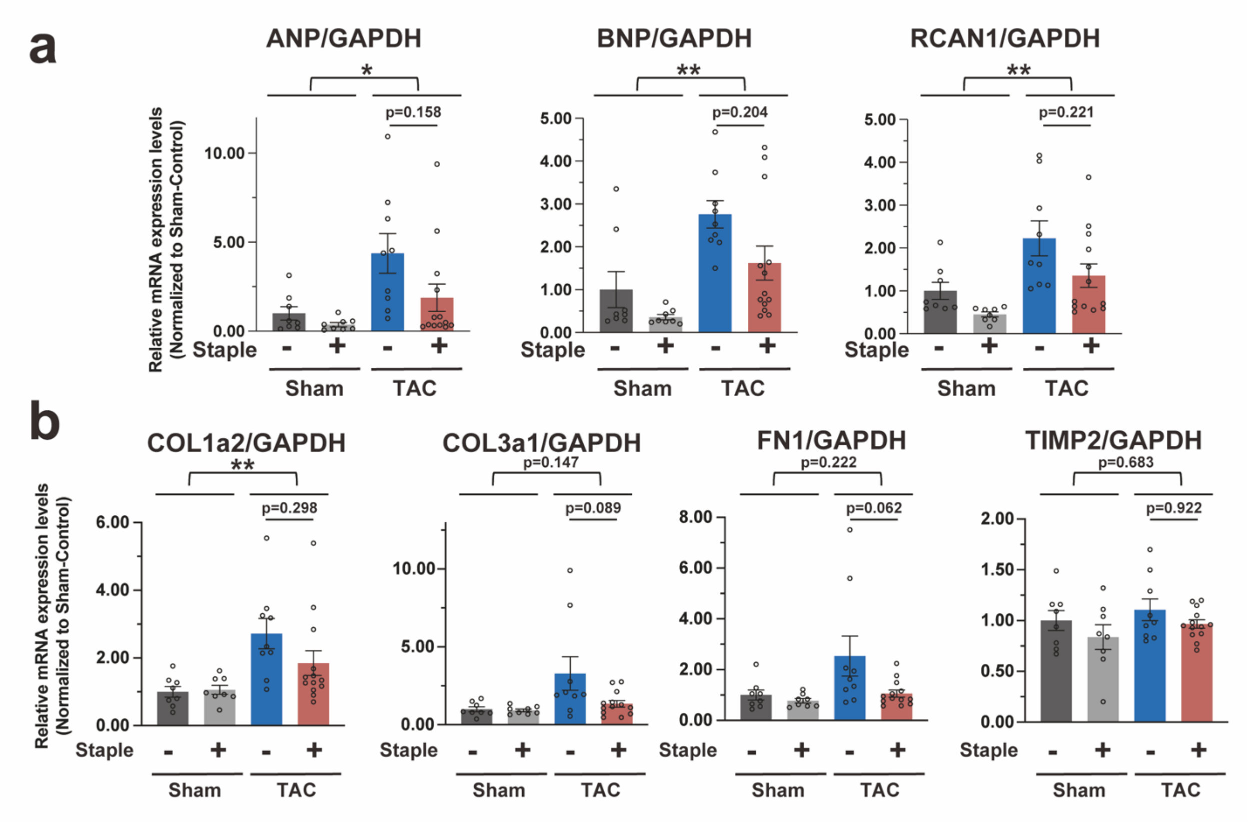

Supplementary Figure 20 | Effects of L-*a*TNA-based Staple oligomers on gene expression in the TAC-treated mouse hearts. a, Evaluation of mRNA expression levels of ANP, BNP, and RCAN1, known markers of cardiac hypertrophy, by qPCR. The mRNA expression levels were increased by TAC treatment (blue bars), but the presence of 2.0 mg/kg L-*a*TNA-based Staple oligomers suppressed these increases (red bars). Data are shown as mean ± S.E. (n=8 mice/Sham group, n=9 mice/TAC Control, or n=13 mice/TAC Staple, biologically independent samples). Statistical significance was determined by two-way ANOVA (two-sided, ANP; *P=0.0143, BNP; **P=0.00571, RCAN1; **P= 0.00357). **b,** Evaluation of mRNA expression levels of COL1a2, COL3a1, FN1, and TIMP2, known markers of myocardial fibrosis, by qPCR. The mRNA expression levels were increased by TAC treatment (blue bars), but the presence of 2.0 mg/kg L-*a*TNA-based Staple oligomers suppressed these increases (red bars). Data are shown as mean ± S.E. (n=8 mice/Sham group, n=9 mice/TAC Control, or n=13 mice/TAC Staple, biologically independent samples). Statistical significance was determined by two-way ANOVA (COL1a2; **P=0.00185).

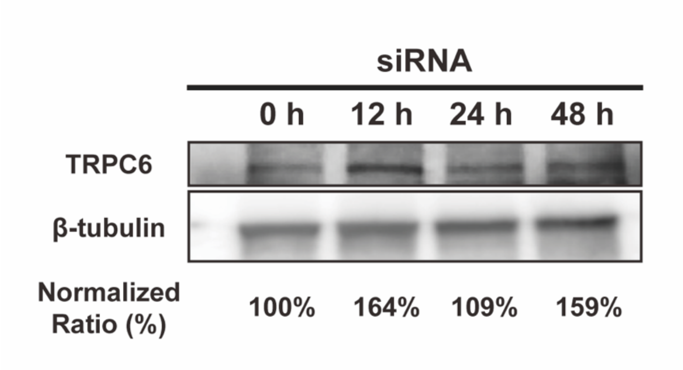

Supplementary Figure 21 | Effect of siRNA on TRPC6 protein expression in NIH3T3 cells. The effects of siRNA for the TRPC6 gene on TRPC6 protein expression in NIH3T3 cells were evaluated by western blotting. Protein expression levels were quantified relative to the expression of β-tubulin using ImageJ software.

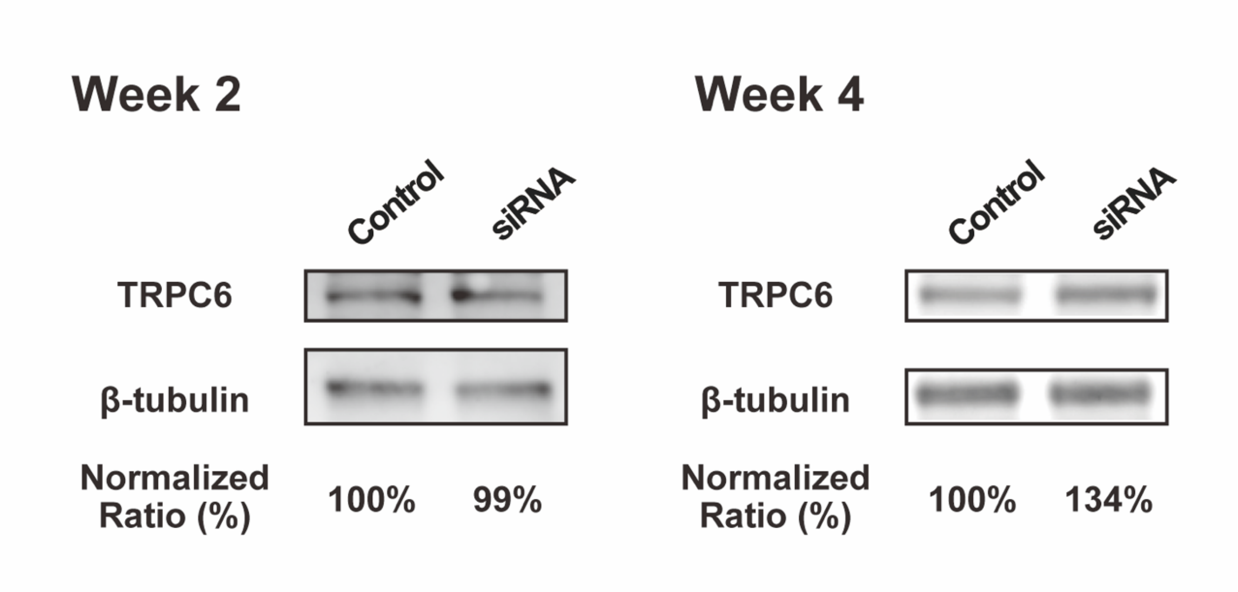

Supplementary Figure 22 | Effects of siRNA on TRPC6 expression in mouse hearts. Western blot analysis revealed that the effect of siRNA on suppression of TRPC6 gene expression was not detectable in the expression levels of TRPC6 at weeks 2 and 4 after AAV6 transduction. Protein expression levels were quantified relative to the expression of β-tubulin using ImageJ software.

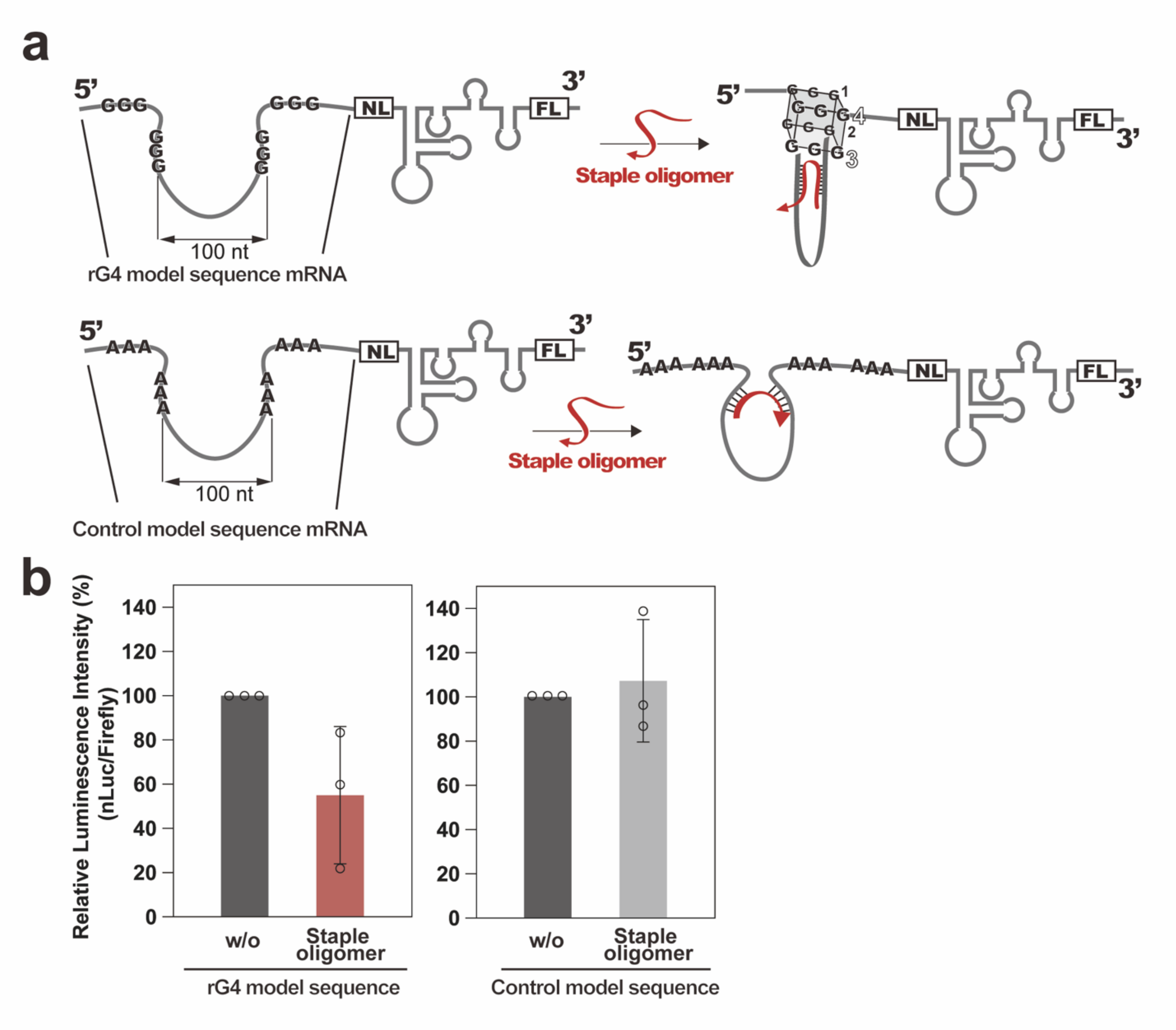

Supplementary Figure 23 | Validation of the cooperation of RNAh technology with RNAi. a, Dual reporter genes encoding Nano luciferase (nLuc) and Firefly luciferase (FL) in tandem were used to validate the cooperation of RNA Staple oligomers in RNAi activation against target RNAs in cells. rG4 and control model sequences were placed in the 5′UTR of nLuc. FL was placed downstream of an IRES as an internal standard. Upon hybridization, RNA Staple oligomers induce rG4 formation in the rG4 model sequence (upper panel), while no such effect is observed in the control model sequence (lower panel). **b,** RNA Staple oligomers do not cooperate in RNAi activation against target RNAs in cells. The RNA Staple oligomers suppressed intracellular nLuc expression in cells from mRNA with the rG4 model sequence (red bar in left panel), but not from mRNA with the control model sequence (gray bar in right panel). Black bars in both panels are shown without RNA Staple oligomer expression. These results strongly suggest that hybridization of RNA Staple oligomers to target mRNAs was independent of the translational downregulation of the target mRNA, indicating that RNAi effects would not be involved in RNAh activity. nLuc luminescence was normalized by FL luminescence. Data are mean ± S.D. of three independent experiments.

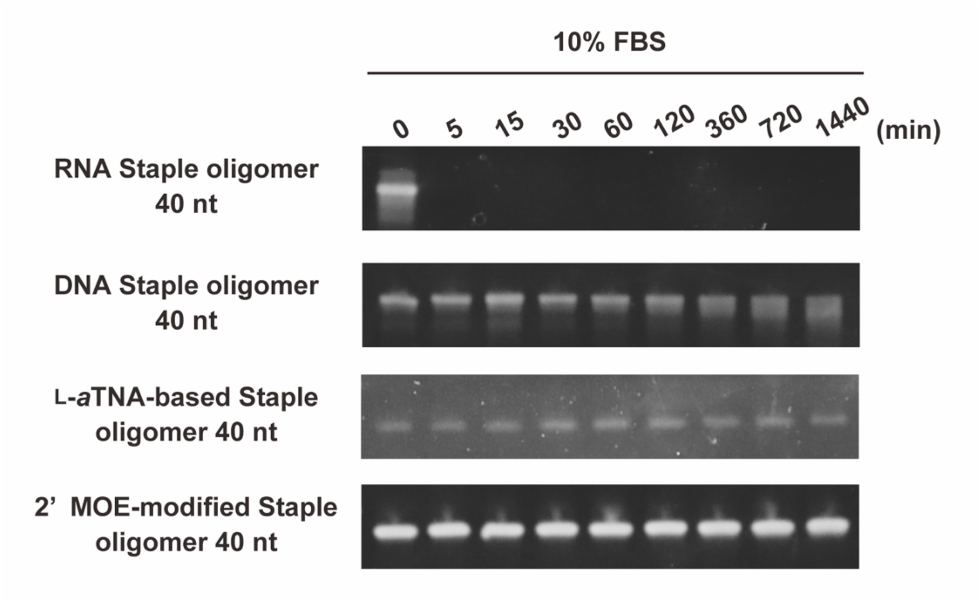

Supplementary Figure 24 | Evaluation of biological stability of various Staple oligomers under 10% FBS conditions by denaturing PAGE. RNA Staple oligomers were degraded within 5 min. DNA Staple oligomers remained stable for up to 60 min. Neither artificial nucleic-acid-based Staple oligomer showed detectable degradation after 24 h.

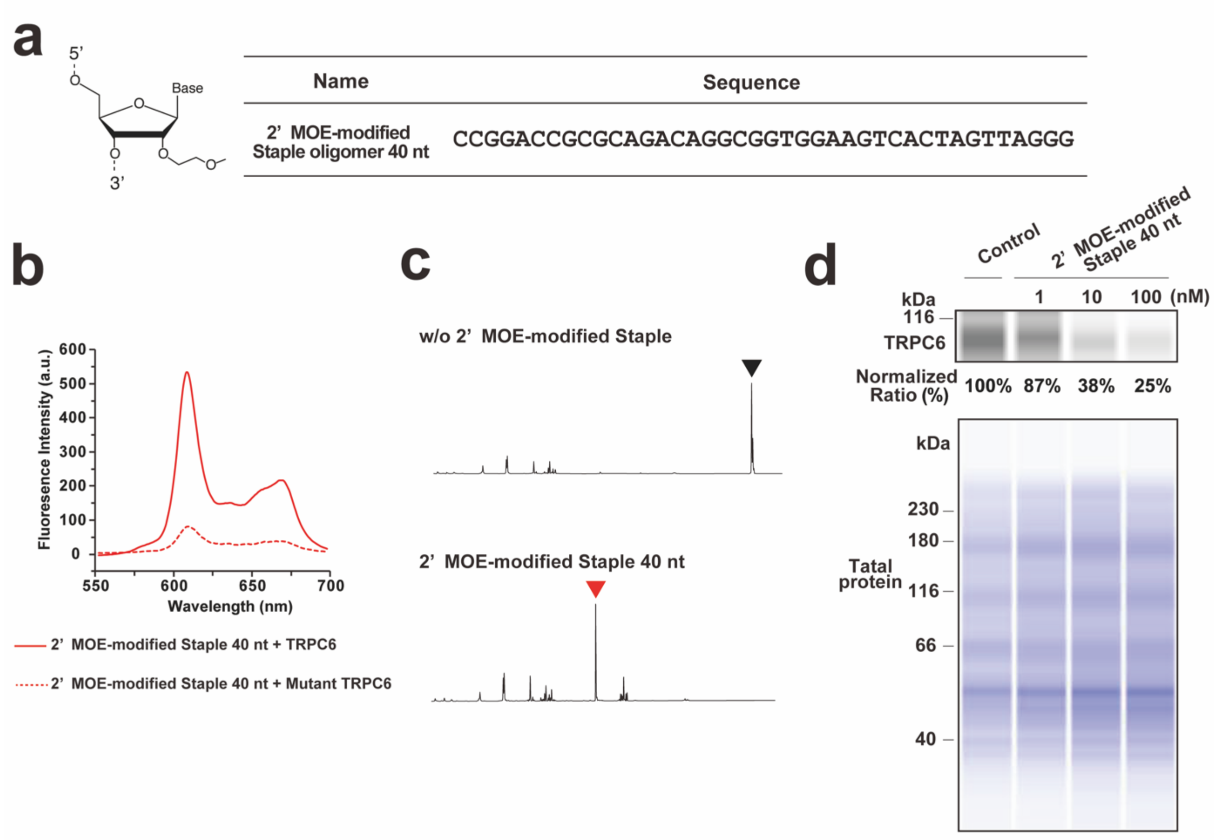

Supplementary Figure 25 | Characterization of RNAh technology with 2′MOE-modified Staple oligomer. a. Chemical structure of 2′MOE and nucleotide sequence of a 40-nt 2′MOE-modified Staple oligomer. **b.** Evaluation of rG4 formation with NMM. The solid and dashed curves show the fluorescence emission spectra of NMM in the presence of the 2′MOE-modified Staple oligomers with TRPC6 sequence and its mutant, respectively. **c.** Identification of rG4 formation with the 2′MOE-modified Staple oligomer on the TRPC6 sequence by RTase stop assay. Red arrowhead indicates the site of arrest of RTase with rG4 induced by the 2′MOE-modified Staple oligomers, and black arrowhead indicates the RTase elongation end without rG4 induction. **d.** Evaluation of the effects of the 2′MOE-modified Staple oligomer on TRPC6 expression in C2C12 cells by Western blotting. TRPC6 expression was suppressed by the 2′MOE-modified Staple oligomers in a dose-dependent manner (upper panel). TRPC6 signals were normalized to total protein (lower panel).

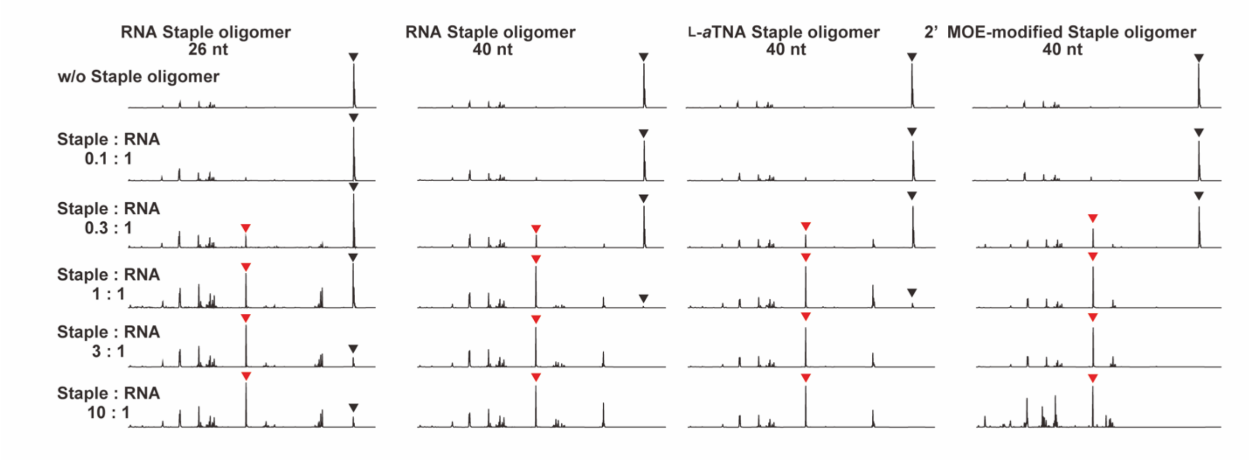

Supplementary Figure 26 | Evaluation of concentration dependence with Staple oligomers. rG4 formation by various concentration of Staple oligomers on the TRPC6 sequence was identified by RTase stop assay. Reverse-transcription reaction was suppressed by induced rG4 in a dose-dependent manner. Red arrowheads indicate the site of arrest of RTase with rG4 induced by Staple oligomers, and black arrowheads indicate the RTase elongation end without rG4 induction.

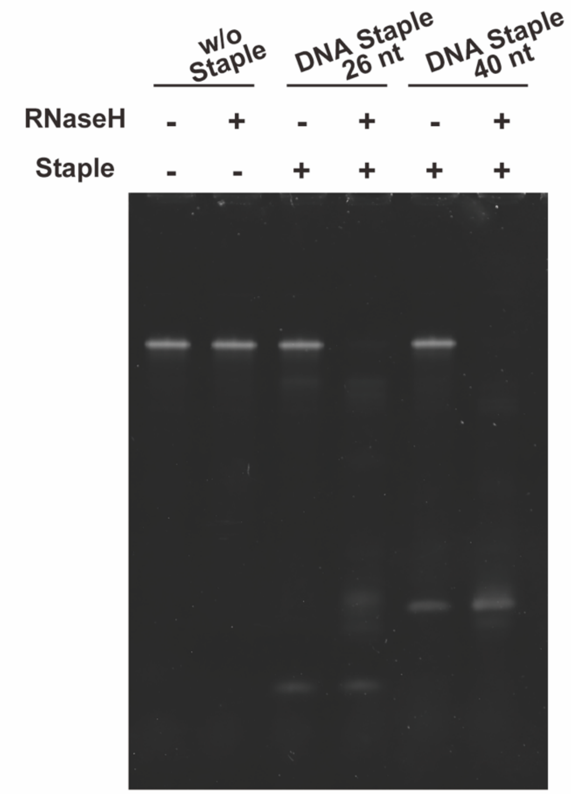

Supplementary Figure 27 | Validation of the cooperation of RNAh technology with RNaseH. DNA Staple oligomers activate RNaseH-mediated cleavage of target RNA. The residual target RNA after RNaseH treatment with DNA Staple oligomers was characterized by denaturing PAGE.

Supplementary Table 1 | Nucleotide sequences of the target RNA. The solid underlines represent the 40-nt Staple oligomer recognition sites.

Names	Sequence
2+2-63nt<br>(rG4 model sequence)	GAUUAGCAUACGCUACUGCAGUGGGUGGGUGUCGACCUAGAUUAAUGCAAUUCGUACGAAGUUCAUAGCAUUUCCAGCACCC<br>AAUUGAAGCUUUGGGUGGGUGCAUGCCAUGACUUGCAAGCUAUUCAGCAGGUAUACAUAGUUGGAA
2+2-63nt<br>(Control model sequence)	GAUUAGCAUACGCUACUGCAGUAAAUAAAUGUCGACCUAGAUUAAUGCAAUUCGUACGAAGUUCAUAGCAUUUCCAGCACCC<br>AAUUGAAGCUUUAAAUAAAUGCAUGCCAUGACUUGCAAGCUAUUCAGCAGGUAUACAUAGUUGGAA
2+2-100nt<br>(rG4 model sequence)	GAUUAGCAUACGCUACUGCAGUGGGUGGGUGUCGACCUAGAUUAAUGCAAUUCGUGUGAAGAUCCUUUUUGAUAAUCUCAUG<br>ACCAAAAUCCCUUAACGUGAGUUCAUUUCCAGCACCCAAUUGAAGCUUUGGGUGGGUGCAUGCCAUGACUUGCAAGCUAUUC<br>AGCAGGUAUACAUAGUUGGAA
2+2-100nt<br>(Control model sequence)	GAUUAGCAUACGCUACUGCAGUAAAUAAAUGUCGACCUAGAUUAAUGCAAUUCGUGUGAAGAUCCUUUUUGAUAAUCUCAUG<br>ACCAAAAUCCCUUAACGUGAGUUCAUUUCCAGCACCCAAUUGAAGCUUUAAAUAAAUGCAUGCCAUGACUUGCAAGCUAUUC<br>AGCAGGUAUACAUAGUUGGAA
2+2-140nt<br>(rG4 model sequence)	GAUUAGCAUACGCUACUGCAGUGGGUGGGUGUCGACCUAGAUUAAUGCAAUUCGUGUGAAGAUCCUUUUUGAUAAUCUCAUG<br>ACCAAAAUCCCUUAACGUGAGUUUUCGUUCCACUGAGCGUCAGACCCCGUAGAAAAGAUCAAACAUUUCCAGCACCCAAUUG<br>AAGCUUUGGGUGGGUGCAUGCCAUGACUUGCAAGCUAUUCAGCAGGUAUACAUAGUUGGAA
2+2-140nt<br>(Control model sequence)	GAUUAGCAUACGCUACUGCAGUAAAUAAAUGUCGACCUAGAUUAAUGCAAUUCGUGUGAAGAUCCUUUUUGAUAAUCUCAUG<br>ACCAAAAUCCCUUAACGUGAGUUUUCGUUCCACUGAGCGUCAGACCCCGUAGAAAAGAUCAAACAUUUCCAGCACCCAAUUG<br>AAGCUUUAAAUAAAUGCAUGCCAUGACUUGCAAGCUAUUCAGCAGGUAUACAUAGUUGGAA
3+1-63nt<br>(rG4 model sequence)	GAUUAGCAUACGCUACUGCAGUGGGUGGGUGGGUGUCGACCUAGAUUAAUGCAAUUCGUACGAAGUUCAUAGCAUUUCCAGC<br>ACCCAAUUGAAGCUUUGGGUGCAUGCCAUGACUUGCAAGCUAUUCAGCAGGUAUACAUAGUUGGAA
3+1-63nt<br>(Control model sequence)	GAUUAGCAUACGCUACUGCAGUAAAUAAAUAAAUGUCGACCUAGAUUAAUGCAAUUCGUACGAAGUUCAUAGCAUUUCCAGC<br>ACCCAAUUGAAGCUUUAAAUGCAUGCCAUGACUUGCAAGCUAUUCAGCAGGUAUACAUAGUUGGAA
3+1-100nt<br>(rG4 model sequence)	GAUUAGCAUACGCUACUGCAGUGGGUGGGUGGGUGUCGACCUAGAUUAAUGCAAUUCGUGUGAAGAUCCUUUUUGAUAAUCU<br>CAUGACCAAAAUCCCUUAACGUGAGUUCAUUUCCAGCACCCAAUUGAAGCUUUGGGUGCAUGCCAUGACUUGCAAGCUAUUC<br>AGCAGGUAUACAUAGUUGGAA
3+1-100nt<br>(Control model sequence)	GAUUAGCAUACGCUACUGCAGUAAAUAAAUAAAUGUCGACCUAGAUUAAUGCAAUUCGUGUGAAGAUCCUUUUUGAUAAUCU<br>CAUGACCAAAAUCCCUUAACGUGAGUUCAUUUCCAGCACCCAAUUGAAGCUUUAAAUGCAUGCCAUGACUUGCAAGCUAUUC<br>AGCAGGUAUACAUAGUUGGAA
3+1-140nt<br>(rG4 model sequence)	GAUUAGCAUACGCUACUGCAGUGGGUGGGUGGGUGUCGACCUAGAUUAAUGCAAUUCGUGUGAAGAUCCUUUUUGAUAAUCU<br>CAUGACCAAAAUCCCUUAACGUGAGUUUUCGUUCCACUGAGCGUCAGACCCCGUAGAAAAGAUCAAACAUUUCCAGCACCCA<br>AUUGAAGCUUUGGGUGCAUGCCAUGACUUGCAAGCUAUUCAGCAGGUAUACAUAGUUGGAA
3+1-140nt<br>(Control model sequence)	GAUUAGCAUACGCUACUGCAGUAAAUAAAUAAAUGUCGACCUAGAUUAAUGCAAUUCGUGUGAAGAUCCUUUUUGAUAAUCU<br>CAUGACCAAAAUCCCUUAACGUGAGUUUUCGUUCCACUGAGCGUCAGACCCCGUAGAAAAGAUCAAACAUUUCCAGCACCCA<br>AUUGAAGCUUUAAAUGCAUGCCAUGACUUGCAAGCUAUUCAGCAGGUAUACAUAGUUGGAA
2+2-200nt<br>(rG4 model sequence)	GAUUAGCAUACGCUACUGCAGUGGGUGGGUGUCGACCUAGAUUAAUGCAAUUCGUGUGAAGAUCCUUUUUGAUAAUCUCAUG<br>ACCAAAAUCCCUUAACGUGAGUUUUCGUUCCACUGAGCGUCAGACCCCGUAGAAAAGAUCAAAGGAUCUUCUUGAGAUCCUU<br>UUUUUCUGCGCGUAAUCUGCUGCUUGCAAACAAAAAAACCACAUUUCCAGCACCCAAUUGAAGCUUUGGGUGGGUGCAUGCC<br>AUGACUUGCAAGCUAUUCAGCAGGUAUACAUAGUUGGAA
1+1-100nt<br>(Control model sequence)	GAUUAGCAUACGCUACUGCAGUGGGUGUCGACCUAGAUUAAUGCAAUUCGUGUGAAGAUCCUUUUUGAUAAUCUCAUGACCA<br>AAAUCCCUUAACGUGAGUUCAUUUCCAGCACCCAAUUGAAGCUUUGGGUGCAUGCCAUGACUUGCAAGCUAUUCAGCAGGUA<br>UACAUAGUUGGAA
5' UTR of TPM3 mRNA	GGUCACAUCCGGGCGGGUUGGUGAGUUCCGGUAUUUCAGGGCGUAGCAGGCGGAAGUAAGGGUGAGAGGAGGCUGCAACGCC<br>GAGCGGAGGAGGCAGGAACCGGAGCGCGAGCAGUAGCUGGGUGGGCACCCAGGUAUACAUAGUUGGAAAUCUCUGGAAGAUC<br>CGCGCGUACCGAGUUCUAAUUCACUGGCCGUCGUUUUA
5' UTR of MYD88 mRNA	GAGAUUCCUACUUCUUACGCCCCCCACAUCACCCGCCUCGAGACCUCAAGGGUAGAGGUGGGCACCCCCGCCUCCGCACUUU<br>UGCUCGGGGCUCCAGAUUGUAGGGCAGGGCGGCGCUUCUCGGAAAGCGAAAGCCGGCGGGGCGGGGCGGGUGCCGCAGGAGA<br>AAGAGGAAGCGCUGGCAGACACAGGUAUACAUAGUUGG
5' UTR of TRPC6 mRNA	CGCCUGUGCCCUCUGCCUGGGAGCCUGGGGCCGCCUGUCUGCGCGGUCCGGAUGCGCUCAGGUCAAGGUUCCUUUCGCGGCU<br>GUCUCCCAAGCCCCUAACUAGUGACUUCCACUGUGGCGGGCAGGGAAGCCAUUGGCAGAACCUAGCCAGUCAGGAAUCUGCA<br>UCUCUUCCCUCAUUAUCCUCUCCCUGGCAUUGCUUUGCUCGGGUCCUCCACGGAAGCAGGGUGCAGGCCGGCCAGGCACUGU<br>GCCAUG
Mutant 5' UTR of TRPC6 mRNA	CGCCUGUGCCCUCUGCCUAAAAGCCUAAAACCGCCUGUCUGCGCGGUCCGGAUGCGCUCAGGUCAAGGUUCCUUUCGCGGCU<br>GUCUCCCAAGCCCCUAACUAGUGACUUCCACUAUAACAAACAAAAAAGCCAUUGGCAGAACCUAGCCAGUCAGGAAUCUGCA<br>UCUCUUCCCUCAUUAUCCUCUCCCUGGCAUUGCUUUGCUCGGGUCCUCCACGGAAGCAGGGUGCAGGCCGGCCAGGCACUGU<br>GCCAUG
CDS of Nano luciferase mRNA	AUGGUCUUCACACUCGAAGAUUUCGUUGGGGACUGGCGACAGACAGCCGGCUACAACCUGGACCAAGUCCUUGAACAGGGAG<br>GGGUGUCCAGUUUGUUUCAGAAUCUCGGGGUGUCCGUAACUCCGAUCCAAAGGAUUGUCCUGAGCGGGGAAAAUGGGCUGAA<br>GAUCGACAUCCAUGUCAUCAUCCCGUAUGAAGGUCUGAGCGGGGACCAAAUGGGCCAGAUCGAAAAAAUUUUUAAGGUGGUG<br>UACCCUGUGGAUGAUCAUCACUUUAAGGUGAUCCUGCACUAUGGCACACUGGUAAUCGACGGGGUUACGCCGAACAUGAUCG<br>ACUAUUUCGGACGGCCGUAUGAAGGCAUCGCCGUGUUCGACGGCAAAAAGAUCACUGUAACAGGGACCCUGUGGAACGGCAA<br>CAAAAUUAUCGACGAGCGCCUGAUCAACCCCGACGGCUCCCUGCUGUUCCGAGUAACCAUCAACGGAGUGACCGGCUGGCGG<br>CUGUGCGAACGCAUUCUGGCGUAA

Supplementary Table 2 | Nucleotide sequences of Staple oligomers for rG4 model sequence, TPM gene, MYD88 gene, and mutated Nano luciferase gene.

Names	Sequences
DNA Staple oligomer 10 nt (rG4 model sequence)	TCGACAAGCT
DNA Staple oligomer 12 nt (rG4 model sequence)	GTCGACAAGCTT
DNA Staple oligomer 14 nt (rG4 model sequence)	GGTCGACAAGCTTC
DNA Staple oligomer 16 nt (rG4 model sequence)	AGGTCGACAAGCTTCA
DNA Staple oligomer 18 nt (rG4 model sequence)	TAGGTCGACAAGCTTCAA
DNA Staple oligomer 20 nt (rG4 model sequence)	CTAGGTCGACAAGCTTCAAT
DNA Staple oligomer 22 nt (rG4 model sequence)	TCTAGGTCGACAAGCTTCAATT
DNA Staple oligomer 24 nt (rG4 model sequence)	ATCTAGGTCGACAAGCTTCAATTG
DNA Staple oligomer 26 nt (rG4 model sequence)	AATCTAGGTCGACAAGCTTCAATTGG
DNA Staple oligomer 28 nt (rG4 model sequence)	TAATCTAGGTCGACAAGCTTCAATTGGG
DNA Staple oligomer 30 nt (rG4 model sequence)	TTAATCTAGGTCGACAAGCTTCAATTGGGT
DNA Staple oligomer 32 nt (rG4 model sequence)	ATTAATCTAGGTCGACAAGCTTCAATTGGGTG
DNA Staple oligomer 34 nt (rG4 model sequence)	CATTAATCTAGGTCGACAAGCTTCAATTGGGTGC
DNA Staple oligomer 36 nt (rG4 model sequence)	GCATTAATCTAGGTCGACAAGCTTCAATTGGGTGCT
DNA Staple oligomer 38 nt (rG4 model sequence)	TGCATTAATCTAGGTCGACAAGCTTCAATTGGGTGCTG
DNA Staple oligomer 40 nt (rG4 model sequence)	TTGCATTAATCTAGGTCGACAAGCTTCAATTGGGTGCTGG
Hybridization oligomer 40 nt (rG4 model sequence)	AAGCTTCAATTGGGTGCTGGTTGCATTAATCTAGGTCGAC
DNA Staple oligomer 40 nt (TPM3)	TGAAATACCGGAACTCACCAGCTACTGCTCGCGCTCCGGT
RNA Staple oligomer 26 nt (TPM3)	CCGGAACUCACCAGCUACUGCUCGCG
DNA Staple oligomer 40 nt (MYD88)	CTTCCTCTTTCTCCTGCGGCACAATCTGGAGCCCCGAGCA
DNA Staple oligomer 40 nt (Nano luciferase)	GACATGGATGTCGATCTTCAGTTCAAGGACTTGGTCCAGG

Supplementary Table 3 | Nucleotide sequences of Staple oligomer or siRNA for TRPC6 gene.

Names	Sequences
DNA Staple oligomer 10 nt	AGGCGGTGGA
RNA Staple oligomer 10 nt	AGGCGGUGGA
DNA Staple oligomer 12 nt	CAGGCGGTGGAA
RNA Staple oligomer 12 nt	CAGGCGGUGGAA
DNA Staple oligomer 14 nt	ACAGGCGGTGGAAG
RNA Staple oligomer 14 nt	ACAGGCGGUGGAAG
DNA Staple oligomer 16 nt	GACAGGCGGTGGAAGT
RNA Staple oligomer 16 nt	GACAGGCGGUGGAAGU
DNA Staple oligomer 18 nt	AGACAGGCGGTGGAAGTC
RNA Staple oligomer 18 nt	AGACAGGCGGUGGAAGUC
DNA Staple oligomer 20 nt	CAGACAGGCGGTGGAAGTCA
RNA Staple oligomer 20 nt	CAGACAGGCGGUGGAAGUCA
DNA Staple oligomer 22 nt	GCAGACAGGCGGTGGAAGTCAC
RNA Staple oligomer 22 nt	GCAGACAGGCGGUGGAAGUCAC
DNA Staple oligomer 24 nt	CGCAGACAGGCGGTGGAAGTCACT
RNA Staple oligomer 24 nt	CGCAGACAGGCGGUGGAAGUCACU
DNA Staple oligomer 26 nt	GCGCAGACAGGCGGTGGAAGTCACTA
RNA Staple oligomer 26 nt	GCGCAGACAGGCGGUGGAAGUCACUA
DNA Staple oligomer 28 nt	CGCGCAGACAGGCGGTGGAAGTCACTAG
RNA Staple oligomer 28 nt	CGCGCAGACAGGCGGUGGAAGUCACUAG
DNA Staple oligomer 30 nt	CCGCGCAGACAGGCGGTGGAAGTCACTAGT
RNA Staple oligomer 30 nt	CCGCGCAGACAGGCGGUGGAAGUCACUAGU
DNA Staple oligomer 32 nt	ACCGCGCAGACAGGCGGTGGAAGTCACTAGTT
RNA Staple oligomer 32 nt	ACCGCGCAGACAGGCGGUGGAAGUCACUAGUU
DNA Staple oligomer 34 nt	GACCGCGCAGACAGGCGGTGGAAGTCACTAGTTA
RNA Staple oligomer 34 nt	GACCGCGCAGACAGGCGGUGGAAGUCACUAGUUA
DNA Staple oligomer 36 nt	GGACCGCGCAGACAGGCGGTGGAAGTCACTAGTTAG
RNA Staple oligomer 36 nt	GGACCGCGCAGACAGGCGGUGGAAGUCACUAGUUAG
DNA Staple oligomer 38 nt	CGGACCGCGCAGACAGGCGGTGGAAGTCACTAGTTAGG
RNA Staple oligomer 38 nt	CGGACCGCGCAGACAGGCGGUGGAAGUCACUAGUUAGG
DNA Staple oligomer 40 nt	CCGGACCGCGCAGACAGGCGGTGGAAGTCACTAGTTAGGG
RNA Staple oligomer 40 nt	CCGGACCGCGCAGACAGGCGGUGGAAGUCACUAGUUAGGG
RNA Staple oligomer 40 nt	Cy5-CCGGACCGCGCAGACAGGCGGUGGAAGUCACUAGUUAGGG-FITC
L-*a*TNA-based Staple oligomer 40 nt-1	CCGGACCGCGCAGACAGGCGGTGGAAGTCACTAGTTAGGG
L-*a*TNA-based Staple oligomer 40 nt-1	Cy5-CCGGACCGCGCAGACAGGCGGTGGAAGTCACTAGTTAGGG-FITC
DNA or L-*a*TNA-based Staple oligomer 40 nt-2	CCGGACCGCGCAGACAGGCGCCACAGTGGAAGTCACTAGT
2' MOE-modified Staple oligomer 40 nt	CCGGACCGCGCAGACAGGCGGTGGAAGTCACTAGTTAGGG
DNA Staple oligomer 42 nt	TCCGGACCGCGCAGACAGGCGGTGGAAGTCACTAGTTAGGGG
RNA Staple oligomer 42 nt	UCCGGACCGCGCAGACAGGCGGUGGAAGUCACUAGUUAGGGG
DNA Staple oligomer 44 nt	ATCCGGACCGCGCAGACAGGCGGTGGAAGTCACTAGTTAGGGGC
RNA Staple oligomer 44 nt	AUCCGGACCGCGCAGACAGGCGGUGGAAGUCACUAGUUAGGGGC
DNA Staple oligomer 46 nt	CATCCGGACCGCGCAGACAGGCGGTGGAAGTCACTAGTTAGGGGCT
RNA Staple oligomer 46 nt	CAUCCGGACCGCGCAGACAGGCGGUGGAAGUCACUAGUUAGGGGCU
DNA Staple oligomer 48 nt	GCATCCGGACCGCGCAGACAGGCGGTGGAAGTCACTAGTTAGGGGCTT
RNA Staple oligomer 48 nt	GCAUCCGGACCGCGCAGACAGGCGGUGGAAGUCACUAGUUAGGGGCUU
DNA Staple oligomer 50 nt	CGCATCCGGACCGCGCAGACAGGCGGTGGAAGTCACTAGTTAGGGGCTTG
RNA Staple oligomer 50 nt	CGCAUCCGGACCGCGCAGACAGGCGGUGGAAGUCACUAGUUAGGGGCUUG
siRNA-1	UUAACAUUGAGGGAAUGAC
siRNA-2	UAAUCUUCUGAGCUCCUUG
siRNA-3	UUCUAAUGAGCUCUGCUAG

Supplementary Methods

Preparation of pUC19-TRPC6-5′UTR and pUC19-TRPC6-5′UTR-mutant Constructs

pUC19-TRPC6-5′UTR:

The mouse TRPC6-5′UTR DNA fragment was prepared by overlap extension PCR. Two double-stranded DNA (dsDNA) fragments (Section1 and Section2) were prepared by DNA polymerase reaction, using synthetic oligonucleotides [5′-TAA TAC GAC TCA CTA TAG GGC GCC TGT GCC CTC TGC CTG GGA GCC TGG GGC CGC CTG TCT GCG CGG TCC GGA TGC GC-3′] and [5′-CCA CAG TGG AAG TCA CTA GTT AGG GGC TTG GGA GAC AGC CGC GAA AGG AAC CTT GAC CTG AGC GCA TCC GGA CCG C-3′] (Section1), or synthetic oligonucleotides [5′-CTA GTG ACT TCC ACT GTG GCG GGC AGG GAA GCC ATT GGC AGA ACC TAG CCA GTC AGG AAT CTG CAT CTC TTC CC-3′] and [5′-GCA CCC CTG CTT CCG TGG AGG ACC CGA GCA AAG CAA TGC CAG GGA GAG GAT AAT GAG GGA AGA GAT GCA GAT TCC-3′] (Section2), respectively. Using the dsDNAs as templates, overlap extension PCR was performed to prepare the TRPC6-5′UTR DNA fragment, using a forward primer [5′-CGG TAC CCG GGG ATC TAA TAC GAC TCA CTA TAG GG-3′] and a reverse primer [5′-CGA CTC TAG AGG ATC GGC ACA GTG CCT GGC CGG CCT GCA CCC CTG C-3′]. The PCR products were subcloned into the BamH I site in pUC19 vector by using HiFi DNA Assembly Cloning Kit (New England Biolabs), then pUC19-TRPC6-5′UTR construct was obtained.

pUC19-TRPC6-5′UTR-mutant:

The mouse TRPC6-5′UTR-mutant DNA fragment was prepared by overlap extension PCR. Four dsDNA fragments (Section3, Section4, Section5 and Section6) were prepared by DNA polymerase reaction, using synthetic oligonucleotides [5′-TAA TAC GAC TCA CTA TAG GGC GCC TGT GCC CTC TGC CTA AAA GCC-3′] and [5′-GCG CAT CCG GAC CGC GCA GAC AGG CGG TTT TAG GCT TTT AGG CAG AG-3′] (Section3), [5′-GCG GTC CGG ATG CGC TCA GGT CAA GGT TCC TTT CGC GGC TGT CTC CC-3′] and [5′-GTT ATA GTG GAA GTC ACT AGT TAG GGG CTT GGG AGA CAG CCG CG-3′] (Section4), [5′-CTA GTG ACT TCC ACT ATA ACA AAC AAA AAA GCC ATT GGC AGA ACC TAG CC-3′] and [5′-GAG AGG ATA ATG AGG GAA GAG ATG CAG ATT CCT GAC TGG CTA GGT TCT GC-3′] (Section5), or [5′-CTC ATT ATC CTC TCC CTG GCA TTG CTT TGC TCG GGT CCT CCA CGG AAG C-3′] and [5′-GAG GAT CGG CAC AGT GCC TGG CCG GCC TGC ACC CCT GCT TCC GTG GAG G-3′] (Section6), respectively. Using each set of the dsDNAs Section3 and Section4, or Section5 and Section6, overlap extension PCR was performed, using a forward primer [5′-CGG TAC CCG GGG ATC TAA TAC GAC TCA CTA TAG GG-3′] and a reverse primer [5′-GTT ATA GTG GAA GTC ACT AG-3′], or a forward primer [5′-CTA GTG ACT TCC ACT ATA AC-3′] and a reverse primer [5′-CGA CTC TAG AGG ATC GGC ACA GTG CCT GGC CGG CCT GCA CCC CTG C-3′], respectively. The two PCR products were used as templates for a subsequent overlap extension PCR. The overlap extension PCR was performed to prepare the TRPC6-5′UTR-mutant DNA fragment, using a forward primer [5′-CGG TAC CCG GGG ATC TAA TAC GAC TCA CTA TAG GG-3′] and a reverse primer [5′-CGA CTC TAG AGG ATC GGC ACA GTG CCT GGC CGG CCT GCA CCC CTG C-3′]. The PCR products were subcloned into the BamH I site in pUC19 vector by using HiFi DNA Assembly Cloning Kit (New England Biolabs), and then pUC19-TRPC6-5′UTR-mutant construct was obtained.

Preparation of pIRES-TRPC6-5′UTR-FL-RL and pIRES-TRPC6-5′UTR-mutant-FL-RL Constructs

pIRES-TRPC6-5′UTR-FL-RL:

The dsDNA for Renilla luciferase (RL) was amplified by PCR using psiCHECK-2 vector (Promega) as a template with a forward primer [5′-TCG ACC CGG GCG GCC ATG GCT TCC AAG GTG TAC G-3′] and a reverse primer [5′-TAA AGG GAA GCG GCC TTA CTG CTC GTT CTT CAG C-3′]. The PCR products were subcloned into the Not I site in pIRES vector (Clontech Laboratories) using the In-Fusion HD cloning kit, and then pIRES-RL construct was obtained. The dsDNA for firefly luciferase (FL) was amplified by PCR using psiCHECK-2 vector as a template with a forward primer [5′-CTA GCC TCG AGA ATT CGC CAC CAT GGC CGA TGC TAA GAA C-3′] and a reverse primer [5′-CTC GAC GCG TGA ATT TTA CAC GGC GAT CTT GCC-3′]. The PCR products were subcloned into the EcoR I site in pIRES-RL constructs using the In-Fusion HD cloning kit, and then pIRES-FL-RL construct was obtained.

pIRES-TRPC6-5′UTR-mutant-FL-RL:

The mouse TRPC6-5′UTR and TRPC6-5′UTR-mutant DNA fragments were prepared by overlap extension PCR. The TRPC6-5′UTR DNA fragment was amplified by PCR with a forward primer [5′-CGA CTC ACT ATA GGC TAG CCC GCC TGT GCC CTC TG-3′] and a reverse primer [5′-TCG GCC ATG GTG GCG AAT

TCG GCA CAG TGC CTG G-3′] using pUC19-TRPC6-5′UTR construct as a template. The TRPC6-5′UTR-mutant DNA was amplified by PCR with a forward primer [5′-CGA CTC ACT ATA GGC TAG CCC GCC TGT GCC CTC TG-3′] and a reverse primer [5′-TCG GCC ATG GTG GCG AAT TCG GCA CAG TGC CTG G-3′] using pUC19-TRPC6-5′UTR-mutant construct as a template. Each PCR products was subcloned into the EcoR I site in pIRES-FL-RL construct using HiFi DNA Assembly Cloning Kit (New England Biolabs), and then pIRES-TRPC6-5′UTR-FL-RL or pIRES-TRPC6-5′UTR-mutant-FL-RL constructs were obtained.

Preparation of pMD19-rG4-model and pMD19-rG4-model-control Constructs

pMD19-rG4-model:

The rG4-model DNA fragment prepared by DNA polymerase reaction, using synthetic oligonucleotides [5′-TAA TAC GAC TCA CTA TAG ATT AGC ATA CGC TAC TGC AGA TGC GC-3′] and [5′-GCT GAA TAG CTT GCA AGT CAT GGC ATG CGC ATC TGC AGT AGC GT-3′]. The dsDNA was subsequently amplified by PCR, using a forward primer [5′-TAA TAC GAC TCA CTA TAG-3′] and a reverse primer [5′-TCC AAC TAT GTA TAC CTG CTG AAT AGC TTG CAA GTC-3′]. The PCR product was subcloned into the EcoR V site in pMD19 vector by using HiFi DNA Assembly Cloning Kit (New England Biolabs), then pMD19-rG4-model construct was obtained.

pMD19-rG4-model-2+2-63 nt:

The 2+2-63nt DNA fragment was prepared by PCR using a synthetic oligonucleotide [5′-ACA CAG GAA ACA GCT ATG ACC ATG ATT ACG CCA AGT TTG CAC GCC TGC CGT TCG ACG ATT TAA TAC GAC TCA CTA TAG ATT AGC ATA CGC TAC TGC AGT GGG TGG GTG TCG ACC TAG ATT AAT GCA ATT CGT ACG AAG TTC ATA GCA TTT CCA GCA CCC AAT TGA AGC TTT GGG TGG GTG CAT GCC ATG ACT TGC AAG CTA TTC AGC AGG TAT ACA TAG TTG GAA ATC TCT GGA AGA TCC GCG CGT ACC GAG TTC TAA TTC ACT GGC CGT CGT TTT ACA ACG TCG TGA CTG GGA AAA CCC TGG CGT TAC CCA-3′] as a template with a forward primer [5′-TAA TAC GAC TCA CTA TAG A-3′] and a reverse primer [5′-TCC AAC TAT GTA TAC CTG-3′]. As vector preparation, the pMD19-rG4-model construct was linearized by PCR using a forward primer [5′-CAG GTA TAC ATA GTT GGA AAT CTC TGG AAG ATC CG-3′] and a reverse primer [5′-CTA TAG TGA GTC GTA TTA AAT CGT CGA ACG GCA GG-3′]. The 2+2-63nt DNA fragment was subcloned into the linearized pMD19-rG4-model vector using HiFi DNA Assembly Cloning Kit (New England Biolabs), and then pMD19-rG4-2+2-63nt construct was obtained.

pMD19-rG4-model-2+2-100 nt:

The 2+2-100nt DNA fragment was prepared by PCR using pUC19 vector as a template with a forward primer [5′-CCT AGA TTA ATG CAA TTC GTG TGA AGA TCC TTT TTG ATA ATC TCA TGA C-3′] and a reverse primer [5′-TCA ATT GGG TGC TGG AAA TGA ACT CAC GTT AAG GGA TTT TGG TCA TGA G-3′]. As vector preparation, the pMD19-rG4-model-2+2-63nt construct was linearized by PCR using a forward primer [5′-CAT TTC CAG CAC CCA ATT GAA GCT T-3′] and a reverse primer [5′-ACG AAT TGC ATT AAT CTA GGT CGA C-3′]. The 2+2-100nt DNA fragment was subcloned into the linearized pMD19-rG4-model-2+2-63nt vector using HiFi DNA Assembly Cloning Kit (New England Biolabs), and then pMD19-model-2+2-100nt construct was obtained.

pMD19-rG4-model-2+2-140nt :

The 2+2-140nt DNA fragment was prepared by PCR using pUC19 vector as a template with a forward primer [5′-CCT AGA TTA ATG CAA TTC GTG TGA AGA TCC TTT TTG ATA ATC TCA TGA C-3′] and a reverse primer [5′-TCA ATT GGG TGC TGG AAA TGT TTG ATC TTT TCT ACG GGG TCT GAC GCT CAG-3′]. The 2+2-140nt DNA fragment was subcloned into the linearized pMD19-rG4-model-2+2-63nt vector using HiFi DNA Assembly Cloning Kit (New England Biolabs), and then pMD19-rG4-model-2+2-140nt construct was obtained.

pMD19-rG4-model-3+1-63nt:

The 3+1-63nt DNA fragment was prepared by PCR using a synthetic oligonucleotide [5′-ACA CAG GAA ACA GCT ATG ACC ATG ATT ACG CCA AGT TTG CAC GCC TGC CGT TCG ACG ATT TAA TAC GAC TCA CTA TAG ATT AGC ATA CGC TAC TGC AGT GGG TGG GTG GGT GTC GAC CTA GAT TAA TGC AAT TCG TAC GAA GTT CAT AGC ATT TCC AGC ACC CAA TTG AAG CTT TGG GTG CAT GCC ATG ACT TGC AAG CTA TTC AGC AGG TAT ACA TAG TTG GAA ATC TCT GGA AGA TCC GCG CGT ACC GAG TTC TAA TTC ACT GGC CGT CGT TTT ACA ACG TCG TGA CTG GGA AAA CCC TGG CGT TAC CCA-3′] as a template with a forward primer [5′-TAA TAC GAC TCA CTA TAG AA-3′] and a reverse primer [5′-TCC AAC TAT GTA TAC CTG-3′]. The 3+1-63nt DNA fragment was subcloned into the linearized

pMD19-rG4-model vector using HiFi DNA Assembly Cloning Kit (New England Biolabs), and then pMD19-rG4-3+1-63nt construct was obtained.

pMD19-rG4-model-3+1-100nt:

The 3+1-100nt DNA fragment was prepared by PCR using pUC19 vector as a template with a forward primer [5′-CCT AGA TTA ATG CAA TTC GTG TGA AGA TCC TTT TTG ATA ATC TCA TGA C-3′] and a reverse primer [5′-TCA ATT GGG TGC TGG AAA TGA ACT CAC GTT AAG GGA TTT TGG TCA TGA G-3′]. As vector preparation, the pMD19-rG4-model-3+1-63nt construct was linearized by PCR using a forward primer [5′-CAT TTC CAG CAC CCA ATT GAA GCT T-3′] and a reverse primer [5′-ACG AAT TGC ATT AAT CTA GGT CGA C-3′]. The 3+1-100nt DNA fragment was subcloned into the linearized pMD19-rG4-model-3+1-63nt vector using HiFi DNA Assembly Cloning Kit (New England Biolabs), and then pMD19-model-3+1-100nt construct was obtained.

pMD19-rG4-model-3+1-140nt:

The 3+1-140nt DNA fragment was prepared by PCR using pUC19 vector as a template with a forward primer [5′-CCT AGA TTA ATG CAA TTC GTG TGA AGA TCC TTT TTG ATA ATC TCA TGA C-3′] and a reverse primer [5′-TCA ATT GGG TGC TGG AAA TGT TTG ATC TTT TCT ACG GGG TCT GAC GCT CAG-3′]. The 3+1-140nt DNA fragment was subcloned into the linearized pMD19-rG4-model-3+1-63nt vector using HiFi DNA Assembly Cloning Kit (New England Biolabs), and then pMD19-rG4-model-3+1-140nt construct was obtained.

pMD19-Control-model-2+2-63nt:

The 2+2-63nt-control insert DNA was prepared by PCR using a synthetic oligonucleotide [5′-ACA CAG GAA ACA GCT ATG ACC ATG ATT ACG CCA AGT TTG CAC GCC TGC CGT TCG ACG ATT TAA TAC GAC TCA CTA TAG ATT AGC ATA CGC TAC TGC AGT AAA TAA ATG TCG ACC TAG ATT AAT GCA ATT CGT ACG AAG TTC ATA GCA TTT CCA GCA CCC AAT TGA AGC TTT AAA TAA ATG CAT GCC ATG ACT TGC AAG CTA TTC AGC AGG TAT ACA TAG TTG GAA ATC TCT GGA AGA TCC GCG CGT ACC GAG TTC TAA TTC ACT GGC CGT CGT TTT ACA ACG TCG TGA CTG GGA AAA CCC TGG CGT TAC CCA-3′] as a template with a forward primer [5′-TAA TAC GAC TCA CTA TAG AA-3′] and a reverse primer [5′-TCC AAC TAT GTA TAC CTG-3′]. As a vector preparation, the pMD19-rG4-model construct was linearized by PCR, using a forward primer [5′-CAG GTA TAC ATA GTT GGA AAT CTC TGG AAG ATC CG-3′] and a reverse primer [5′-CTA TAG TGA GTC GTA TTA AAT CGT CGA ACG GCA GG-3′]. The 2+2-63-nt-control DNA fragment was subcloned into the linearized pMD19-rG4-model vector using HiFi DNA Assembly Cloning Kit (New England Biolabs), and then pMD19-model-2+2-63nt control construct was obtained.

pMD19-Control-model-2+2-100nt:

The 2+2-100nt-control insert DNA was prepared by PCR using pUC19 vector as a template with a forward primer [5′-CCT AGA TTA ATG CAA TTC GTG TGA AGA TCC TTT TTG ATA ATC TCA TGA C-3′] and a reverse primer [5′-TCA ATT GGG TGC TGG AAA TGA ACT CAC GTT AAG GGA TTT TGG TCA TGA G-3′]. As a vector preparation, pMD19-2+2-63nt-control construct was linearized by PCR, using a forward primer [5′-CAT TTC CAG CAC CCA ATT GAA GCT T-3′] and a reverse primer [5′-ACG AAT TGC ATT AAT CTA GGT CGA C-3′]. The 2+2-100-nt-control DNA fragment was subcloned into the linearized pMD19-2+2-63nt-control vector using HiFi DNA Assembly Cloning Kit (New England Biolabs), and then pMD19-model-2+2-100-nt-control constructs were obtained.

pMD19-Control-model-2+2-140nt:

The 2+2-140nt-control insert DNA was prepared by PCR using pUC19 vector as a template with a forward primer [5′-CCT AGA TTA ATG CAA TTC GTG TGA AGA TCC TTT TTG ATA ATC TCA TGA C-3′] and a reverse primer [5′-TCA ATT GGG TGC TGG AAA TGT TTG ATC TTT TCT ACG GGG TCT GAC GCT CAG-3′]. The 2+2-140-nt-control DNA fragment was subcloned into the linearized pMD19-2+2-63nt-control vector using HiFi DNA Assembly Cloning Kit (New England Biolabs), and then pMD19-model-2+2-140-nt-control constructs were obtained.

pMD19-Control-model-3+1-63nt:

The 3+1-63nt-control insert DNA was prepared by PCR using a synthetic oligonucleotide [5′-ACA CAG GAA ACA GCT ATG ACC ATG ATT ACG CCA AGT TTG CAC GCC TGC CGT TCG ACG ATT TAA TAC GAC TCA CTA TAG ATT AGC ATA CGC TAC TGC AGT AAA TAA ATA AAT GTC GAC CTA GAT TAA TGC AAT TCG TAC GAA GTT CAT AGC ATT TCC AGC ACC CAA TTG AAG CTT TAA ATG CAT GCC ATG ACT TGC AAG CTA TTC AGC AGG TAT ACA TAG TTG GAA ATC TCT GGA AGA TCC GCG CGT ACC GAG

TTC TAA TTC ACT GGC CGT CGT TTT ACA ACG TCG TGA CTG GGA AAA CCC TGG CGT TAC CCA-3′] as a template with a forward primer [5′-TAA TAC GAC TCA CTA TAG AA-3′] and a reverse primer [5′-TCC AAC TAT GTA TAC CTG-3′]. As a vector preparation, the pMD19-rG4-model construct was linearized by PCR, using a forward primer [5′-CAG GTA TAC ATA GTT GGA AAT CTC TGG AAG ATC CG-3′] and a reverse primer [5′-CTA TAG TGA GTC GTA TTA AAT CGT CGA ACG GCA GG-3′]. The 3+1-63-nt-control DNA fragment was subcloned into the linearized pMD19-rG4-model vector using HiFi DNA Assembly Cloning Kit (New England Biolabs), and then pMD19-model-3+1-63nt control construct was obtained.

pMD19-Control-model-3+1-100nt:

The 3+1-100nt-control insert DNA was prepared by PCR using pUC19 vector as a template with a forward primer [5′-CCT AGA TTA ATG CAA TTC GTG TGA AGA TCC TTT TTG ATA ATC TCA TGA C-3′] and a reverse primer [5′-TCA ATT GGG TGC TGG AAA TGA ACT CAC GTT AAG GGA TTT TGG TCA TGA G-3′]. As a vector preparation, pMD19-3+1-63nt-control construct was linearized by PCR, using a forward primer [5′-CAT TTC CAG CAC CCA ATT GAA GCT T-3′] and a reverse primer [5′-ACG AAT TGC ATT AAT CTA GGT CGA C-3′]. The 3+1-100-nt-control DNA fragment was subcloned into the linearized pMD19-3+1-63nt-control vector using HiFi DNA Assembly Cloning Kit (New England Biolabs), and then pMD19-model-3+1-100-nt-control constructs were obtained.

pMD19-Control-model-3+1-140nt:

The 3+1-140nt-control insert DNA was prepared by PCR using pUC19 vector as a template with a forward primer [5′-CCT AGA TTA ATG CAA TTC GTG TGA AGA TCC TTT TTG ATA ATC TCA TGA C-3′] and a reverse primer [5′-TCA ATT GGG TGC TGG AAA TGT TTG ATC TTT TCT ACG GGG TCT GAC GCT CAG-3′]. The 3+1-140-nt-control DNA fragment was subcloned into the linearized pMD19-3+1-63nt-control vector using HiFi DNA Assembly Cloning Kit (New England Biolabs), and then pMD19-model-3+1-140-nt-control constructs were obtained.

Preparation of pIRES-model-5′UTR-FL-RL Constructs

Six model-5′UTR DNA fragments (2+2-63nt, 2+2-100nt, 2+2-140nt, 3+1-63nt, 3+1-100nt, 3+1-140nt) were amplified by PCR using pMD19-rG4-model constructs as templates, with a forward primer [5′-CTA GCC TCG AGA ATT GAT TAG CAT ACG CTA CTG C-3′] and a reverse primer [5′-CCA TGG TGG CGA ATT CTG AAT AGC TTG CAA GTC AT-3′]. Each PCR product was subcloned into the EcoR I site in pIRES-FL-RL construct by using HiFi DNA Assembly Cloning Kit (New England Biolabs), and then pIRES-model-5′UTR-2+2-63nt-FL-RL, pIRES-model-5′UTR-100nt-FL-RL, and pIRES-model-5′UTR-140nt-FL-RL constructs and pIRES-model-5′UTR-3+1-63nt-FL-RL, pIRES-model-5′UTR-100nt-FL-RL, and pIRES-model-5′UTR-140nt-FL-RL constructs were obtained.

Preparation of pIRES-model-5′UTR-NL-FL Constructs

rG4 model-5′UTR DNA fragments (2+2-100nt) were amplified by PCR using pMD19-rG4-model-2+2-100nt or pMD19-Control-model-2+2-100nt constructs as templates, with a forward primer [5′-ATA GGC TAG CCA TTA GCA TAC GCT ACT GC-3′] and a reverse primer [5′-GAA GAC CAT GAA TTC CTG AAT AGC TTG CAA GTC-3′]. Each PCR product was subcloned into the Xho I site in pIRES-NL-FL construct by using HiFi DNA Assembly Cloning Kit (New England Biolabs), and then pIRES-model-5′UTR-2+2-100nt-NL-FL constructs were obtained.

Preparation of pIRES-NL-RL Constructs

The dsDNA fragments (Section1 and Section2) for Nano luciferase (NL) that was forcibly mutated to possess four G-tracts, were amplified by PCR using psiCHECK-2 vector (Promega) as a template with a forward primer [5′-ATG GTC TTC ACA CTC GAA GAT TTC G-3′] and a reverse primer [5′-CAA ACT GGA CAC CCC TCC CTG TTC-3′] (Section1), [5′-GAA CAG GGA GGG GTG TCC AGT TTG-3′] and [5′-GAT CTT CAG CCC ATT TTC CCC GCT CAG GAC-3′] (Section2), [5′-GTC CTG AGC GGG GAA AAT GGG CTG AAG ATC G-3′] and [5′-GGC CCA TTT GGT CCC CGC TCA GAC C-3′] (Section3) , [5′-GGT CTG AGC GGG GAC CAA ATG GGC C-3′] and [5′-TCG GCC ATG GTG GCG AAT TCT TAC GCC AGA ATG CGT TCG C-3′] (Section4). Using each set of the dsDNAs Section1 and Section2, or Section3 and Section4, overlap extension PCR was performed, using a forward primer [5′-CGA CTC ACT ATA GGC TAG CCG CCA CCA TGG TCT TCA CAC TCG AAG-3′] and a reverse primer [5′-GAT CTT CAG CCC ATT TTC CCC GCT CAG GAC-3′], or a forward primer [5′-GTC CTG AGC GGG GAA AAT GGG CTG AAG ATC G-3′] and a reverse primer [5′-TCG GCC ATG GTG GCG AAT TCT TAC GCC AGA ATG CGT TCG C-3′], respectively. The two PCR products were used as templates for a subsequent overlap extension PCR. The overlap

extension PCR was performed to prepare the TRPC6-5′UTR-mutant DNA fragment, using a forward primer [5′-CGA CTC ACT ATA GGC TAG CCG CCA CCA TGG TCT TCA CAC TCG AAG-3′] and a reverse primer [5′-TCG GCC ATG GTG GCG AAT TCT TAC GCC AGA ATG CGT TCG C-3′]. The PCR products were subcloned into the *Eco*R I site in pIRES-RL vector by using HiFi DNA Assembly Cloning Kit (New England Biolabs), and then pIRES-NL-RL construct was obtained.

Construction of Staple Oligomer and siRNA Expression Vectors

pAAV-TRPC6-Staple:

The dsDNA fragments of the 26-nt and 40-nt Staple oligomers were prepared by annealing with synthetic oligonucleotides [5′-GAG AAA AGC CTC TAG GCG CAG ACA GGC GGT GGA AGT CAC TAT TTT TTC TAG TGA TAT CGA TA-3′] and [5′-TAT CGA TAT CAC TAG AAA AAA TAG TGA CTT CCA CCG CCT GTC TGC GCC TAG AGG CTT TTC TC-3′], and [5′-GAG AAA AGC CTC TAG ACC GGA CCG CGC AGA CAG GCG GTG GAA GTC ACT AGT TAG GGT TTT TTC TAG TGA TAT CGA TA-3′] and [5′-TAT CGA TAT CAC TAG AAA AAA CCC TAA CTA GTG ACT TCC ACC GCC TGT CTG CGC GGT CCG GTC TAG AGG CTT TTC TC-3′], respectively. Each dsDNA was subcloned into the Xba I-Spe I site in a pAAV-U6-ZsGreen1 vector (Takara Bio) using HiFi DNA Assembly Cloning Kit (New England Biolabs), and then pAAV-TRPC6-Staple-26-nt and -40-nt constructs were obtained.

pSuper-TPM3-Staple:

The dsDNA fragment of the 26-nt Staple oligomer was prepared by annealing with synthetic oligonucleotides [5′-GAT CCC CCC GGA ACT CAC CAG CTA CTG CTC GCG TTT TTA-3′] and [5′-AGC TTA AAA ACG CGA GCA GTA GCT GGT GAG TTC CGG GGG-3′]. The dsDNA was subcloned into the Bgl II-Hind III site in pSuper neo vector (Oligoengine) using DNA Ligation Kit (Takara Bio), and then pSuper-TPM3-Staple-26-nt construct was obtained.

pAAV-rG4-model:

The dsDNA fragments of the 40-nt Staple oligomers were prepared by annealing with synthetic oligonucleotides [5′-GAG AAA AGC CTC TAG GTT GCA TTA ATC TAG GTC GAC AAG CTT CAA TTG GGT GCT GGT TTT TTC TAG TGA TAT CGA TA-3′] and [5′-TAT CGA TAT CAC TAG AAA AAA CCA GCA CCC AAT TGA AGC TTG TCG ACC TAG ATT AAT GCA ACC TAG AGG CTT TTC TC-3′]. The dsDNA was subcloned into the Xba I-Spe I site in a pAAV-U6-ZsGreen1 vector (Takara Bio) using HiFi DNA Assembly Cloning Kit (New England Biolabs), and then pAAV-rG4 model-Staple-40-nt construct was obtained.

pAAV-siRNA:

The dsDNA fragments of the mouse TRPC6 siRNA-1, siRNA-2 and siRNA-3, were prepared by DNA polymerase reaction, using synthetic oligonucleotides: siRNA-1, [5′-GAG AAA AGC CTC TAG GTC ATT CCC TCA ATG TTA ACT GTG AAG CCA CAG ATG GG-3′] and [5′-TAT CGA TAT CAC TAG AAA AAA GTC ATT CCC TCA ATG TTA ACC CAT CTG TGG CTT CAC AG-3′]; siRNA-2, [5′-GAG AAA AGC CTC TAG GCA AGG AGC TCA GAA GAT TAC TGT GAA GCC ACA GAT GGG-3′] and [5′-TAT CGA TAT CAC TAG AAA AAA GCA AGG AGC TCA GAA GAT TAC CCA TCT GTG GCT TCA CAG-3′]; and siRNA-3, [5′-GAG AAA AGC CTC TAG GCT AGC AGA GCT CAT TAG AAC TGT GAA GCC ACA GAT GGG-3′] and [5′-TAT CGA TAT CAC TAG AAA AAA GCT AGC AGA GCT CAT TAG AAC CCA TCT GTG GCT TCA CAG-3′]. Each dsDNA was subcloned into the Xba I-Spe I site in pAAV-U6-ZsGreen1 vector (Takara Bio) by using HiFi DNA Assembly Cloning Kit (New England Biolabs), and then pAAV-siRNA-1, -2, and -3 constructs were obtained.

Preparation of RNA Templates for ThT or NMM Fluorescence Assay

The dsDNA for RNA transcription was prepared from pUC19-TRPC6-5′UTR, pUC19-rG4-model or pIRES NL-RL constructs by PCR-amplification with a primer set for TRPC6 5′UTR [5′-TAG AGT ACT TAA TAC GAC TCA CTA TAG GG-3′] and [5′-GGC ACA GTG CCT GGC CGG-3′], a primer set for rG4 model [5′-TAG AGT ACT TAA TAC GAC TCA CTA TAG GG-3′] and [5′-CTG AAT AGC TTG CAA G-3′], and a primer set for Nano luciferase gene [5′-TAG AGT ACT TAA TAC GAC TCA CTA TAG GG-3′] and [5′- TTA CGC CAG AAT GCG TTC GC-3′], respectively. The dsDNAs were transcribed into single stranded RNAs (ssRNAs) using ScriptMAX® Thermo T7 Transcription Kit (Toyobo). The ssRNAs were purified with After Tri Reagent RNA Clean Up Kit (Favorgen).

Preparation of RNA Templates for RTase Stop Assay or RNaseH assay

The dsDNA for RNA transcription was prepared from pUC19-TRPC6-5′UTR, pMD19-rG4-model or pIRES NL-RL constructs by PCR-amplification with primers set for TRPC6 5′UTR [5′-TAG AGT ACT TAA TAC

GAC TCA CTA TAG GG-3′] and [5′-CAG GTC GAC TCT AGA GGA TCC GCC AGG GTT TTC CCA GTC ACG AC-3′], a primer set for rG4 model [5′-TAG AGT ACT TAA TAC GAC TCA CTA TAG GG-3′] and [5′-TGG GTA ACG CCA GGG-3′] and a primer set for Nano luciferase gene [5′-TAG AGT ACT TAA TAC GAC TCA CTA TAG GG-3′] and [5′-CGC CAG GGT TTT CCC AGT CAC GAC AAC CCC GTC GAT TAC CAG TG-3′]. The dsDNA was transcribed to ssRNA using ScriptMAX® Thermo T7 Transcription Kit (Toyobo). The ssRNAs were purified with After Tri Reagent RNA Clean Up Kit (Favorgen).

Preparation of RNA Templates for In Vitro Translation

The dsDNA for RNA transcription was prepared from each pIRES construct by PCR-amplification with a primer set for TRPC6 5′UTR [5′-GCT AGA GTA CTT AAT ACG ACT CAC TAT AGG GCT AGC C-3′] and [5′-TTA CTG CTC GTT CTT C-3′], a primer set for model 5′UTR [5′-TAA TAC GAC TCA CTA TAG GGC TAG CCT CGA G-3′] and [5′-CTC GAC GCG TGA ATT TTA CAC GGC GAT CTT GCC-3′] and a primer set for Nano luciferase [5′- TAG AGT ACT TAA TAC GAC TCA CTA TAG GG-3′] and [5′- AAC CCT CAC TAA AGG GAA GCT TAC TGC TCG TTC TTC AG-3′]. The ssRNAs were transcribed from the dsDNA templates using ScriptMAX® Thermo T7 Transcription Kit (Toyobo). The ssRNAs were purified with After Tri Reagent RNA Clean Up Kit (Favorgen).
