# Supplementary material for: Staple oligomers induce a stable RNA G-quadruplex structure for protein translation inhibition in therapeutics
Source: Nat Biomed Eng. 2025 Oct 15;10(6):1124–35. doi: 10.1038/s41551-025-01515-4 (PMC13279272; doi:10.1038/s41551-025-01515-4)
Supplement: Supplementary file 2 — Reporting Summary [file 41551_2025_1515_MOESM2_ESM.pdf]

nature portfolio

Corresponding author(s): Yousuke Katsuda, Toshihiro Ihara, Masaki Hagihara, Shin-ichi Sato

Last updated by author(s): May 12, 2025

Reporting Summary

Nature Portfolio wishes to improve the reproducibility of the work that we publish. This form provides structure for consistency and transparency in reporting. For further information on Nature Portfolio policies, see our Editorial Policies and the Editorial Policy Checklist.

Statistics

For all statistical analyses, confirm that the following items are present in the figure legend, table legend, main text, or Methods section.

n/a	Confirmed	
☐	☒	The exact sample size (*n*) for each experimental group/condition, given as a discrete number and unit of measurement
☐	☒	A statement on whether measurements were taken from distinct samples or whether the same sample was measured repeatedly
☐	☒	The statistical test(s) used AND whether they are one- or two-sided<br>*Only common tests should be described solely by name; describe more complex techniques in the Methods section.*
☐	☒	A description of all covariates tested
☒	☐	A description of any assumptions or corrections, such as tests of normality and adjustment for multiple comparisons
☐	☒	A full description of the statistical parameters including central tendency (e.g. means) or other basic estimates (e.g. regression coefficient) AND variation (e.g. standard deviation) or associated estimates of uncertainty (e.g. confidence intervals)
☐	☒	For null hypothesis testing, the test statistic (e.g. *F*, *t*, *r*) with confidence intervals, effect sizes, degrees of freedom and *P* value noted<br>*Give P values as exact values whenever suitable.*
☒	☐	For Bayesian analysis, information on the choice of priors and Markov chain Monte Carlo settings
☒	☐	For hierarchical and complex designs, identification of the appropriate level for tests and full reporting of outcomes
☒	☐	Estimates of effect sizes (e.g. Cohen's *d*, Pearson's *r*), indicating how they were calculated

Our web collection on statistics for biologists contains articles on many of the points above.

Software and code

Policy information about availability of computer code

Data collection	GeneMapper Software 6 was used to collect RTase stop assay.<br>M-mode on Aplio300 was used to collect Echocardiography.<br>LAS X Mica 6.1.0 was used to collect fluorescent evaluation of cardiac delivery of RNA or L-aTNA-based Staple oligomer.
Data analysis	Microsoft Excel (v 16.78) was used to compile experimental data and two-way ANOVA analysis.<br>Microsoft Excel (v 16.78) was used to mRNA-seq experimental data analysis.<br>R (v.4.2.2) was used to compile ANCOVA, two-way ANOVA, Student's t-tests analysis and p values.<br>GeneMapper Software 6 was used to analyze RTase Stop assay fragments, and the traces were later manually adjusted using Adobe Illustrator (2023).<br>ImageJ 1.53a was used to perform Western blot analysis.<br>Compass for SW (v 6.1.0) was used to perform Western Blot analysis using the Abby Protein Simple System.

For manuscripts utilizing custom algorithms or software that are central to the research but not yet described in published literature, software must be made available to editors and reviewers. We strongly encourage code deposition in a community repository (e.g. GitHub). See the Nature Portfolio guidelines for submitting code & software for further information.

Data

Policy information about availability of data

All manuscripts must include a data availability statement. This statement should provide the following information, where applicable:

- Accession codes, unique identifiers, or web links for publicly available datasets
- A description of any restrictions on data availability
- For clinical datasets or third party data, please ensure that the statement adheres to our policy

The raw data are provided as Source Data.
Bioinformatic analysis resources are described in Code Availability Section.
This manuscript ensures that all data and relevant information from this study are fully disclosed.

Research involving human participants, their data, or biological material

Policy information about studies with human participants or human data. See also policy information about sex, gender (identity/presentation), and sexual orientation and race, ethnicity and racism.

Reporting on sex and gender	No human participants, their data were involved in this study.
Reporting on race, ethnicity, or other socially relevant groupings	No human participants, their data were involved in this study.
Population characteristics	See above
Recruitment	No human participants, their data were involved in this study.
Ethics oversight	No human participants, their data were involved in this study.

Note that full information on the approval of the study protocol must also be provided in the manuscript.

Field-specific reporting

Please select the one below that is the best fit for your research. If you are not sure, read the appropriate sections before making your selection.

☒ Life sciences ☐ Behavioural & social sciences ☐ Ecological, evolutionary & environmental sciences

For a reference copy of the document with all sections, see nature.com/documents/nr-reporting-summary-flat.pdf

Life sciences study design

All studies must disclose on these points even when the disclosure is negative.

Sample size	Statistical methods were not used to predetermine sample size. The sample size was determined based on previously published studies using the TAC model. All sample sizes are provided in the figure legends.
Data exclusions	In the TAC mouse experiments, Control groups that did not show a decrease in FS values to 50 or below after TAC were excluded. In the experiments using AAV in mice, samples with significantly low expression of ZsGreen mRNA were excluded, considering them to have low expression of Staple oligomer. The expression level of ZsGreen mRNA was determined based on the difference in Ct values with GAPDH mRNA (ΔCt value), and samples with a ΔCt value greater than 12 were excluded.
Replication	All attempts at replication were successful, and each main result was at least a duplicate of experiments.
Randomization	In the mouse experiments, the mice used were grouped to ensure uniformity in body weight at the time of introduction.
Blinding	The investigators remained blinded to group allocation throughout data acquisition and the initial analysis of FS values, ensuring unbiased data collection. However, blinding could not be maintained during the final statistical analysis because comparing outcomes between groups required knowledge of the group assignments. Group codes were therefore revealed at the analysis stage to allow for accurate group-wise statistical comparisons, after all raw data had been collected and processed in a blinded manner. This approach minimized bias in measurements while acknowledging the necessity of unblinding for the conclusive data analysis.

Reporting for specific materials, systems and methods

We require information from authors about some types of materials, experimental systems and methods used in many studies. Here, indicate whether each material, system or method listed is relevant to your study. If you are not sure if a list item applies to your research, read the appropriate section before selecting a response.

Materials & experimental systems

n/a	Involved in the study
☐	☒ Antibodies
☐	☒ Eukaryotic cell lines
☒	☐ Palaeontology and archaeology
☐	☒ Animals and other organisms
☒	☐ Clinical data
☒	☐ Dual use research of concern
☒	☐ Plants

Methods

n/a	Involved in the study
☒	☐ ChIP-seq
☒	☐ Flow cytometry
☒	☐ MRI-based neuroimaging

Antibodies

Antibodies used

1 .Anti-TRPC6 (Cell Signaling Technology, 16716, Lot:1)
2. Anti-TRPC6 (Proteintech, 18236-1-AP, Lot:00064901)
3. Anti-TRPC6 (Alomone Labs, ACC-017, Lot:ACC017AN4902)
4.Anti-TRPC6 (Alomone Labs, ACC-017, Lot:ACC017AN5002)
4. Anti-β-Tubulin (Proteintech, 66240-1-Ig, Lot:10004491)
5. Anti-β-Tubulin (Proteintech, 66240-1-Ig, Lot:10020237)
6. Anti-TPM3 (Abcam, ab180813, Lot: GR3207729-2)
7. Anti-rabit IgG, HRP-linked Antibody (Cell Signaling Technology, 7074S, Lot:28)
8. Anti-rabit IgG, HRP-linked Antibody (Cell Signaling Technology, 7074S, Lot:31)
9. Amersham ECL Anti-Mouse IgG, Horseradish Peroxidase-Linked Species-Specific Whole Antibody (GE Healthcare, NA931, Lot:16912704)
10. Amersham ECL Anti-Mouse IgG, Horseradish Peroxidase-Linked Species-Specific Whole Antibody (GE Healthcare, NA931, Lot:17556470)

Validation

All commercial antibodies were validated by their suppliers: Proteintech, Abcam, Cell Siganling, Alomone Labs and GE Healthcare.All antibodies are subjected to re-verification in a laboratory setting prior to their utilization.

Eukaryotic cell lines

Policy information about cell lines and Sex and Gender in Research

Cell line source(s)

MCF-7 (RCB, Human metastatic mammary carcinoma)
C2C12 (RCB, mouse striated muscle)
NIH3T3(RCB, NIH Swiss mouse embryo)
HEK293T(RCB, human embryonic kidney)

Authentication

None of the cell lines used were authenticated.

Mycoplasma contamination

Cell lines were not tested for mycoplasma contamination.

Commonly misidentified lines
(See ICLAC register)

No commonly misidentified lines were used.

Animals and other research organisms

Policy information about studies involving animals; ARRIVE guidelines recommended for reporting animal research, and Sex and Gender in Research

Laboratory animals

Male BALB/c and C57BL/6J mice were purchased from Oriental Yeast Co., Ltd and CLEA JAPAN Inc, respectively.BLAB/c (6-week-old) or C57BL/6J (7-week-old) mice were used for in vivo translation assay or for phenotype evaluation, respectively.

Wild animals

The study did not involve wild animals

Reporting on sex

In this study, considering the female reproductive cycle and hormonal balance, we limited our experiments to male subjects only.

Field-collected samples

The study did not involve samples collected from the field.

Ethics oversight

All animal procedures were performed in accordance with Kumamoto University animal care guidelines and the Guide for the Care and Use of Laboratory Animals published by the by the U.S. National Institutes of Health (Publication No. 85-23, revised 1996) and permitted by Animal Care and Use Committee of Kumamoto University.

Note that full information on the approval of the study protocol must also be provided in the manuscript.

Plants

Seed stocks	No plants samples were involved in this study.
Novel plant genotypes	No plants samples were involved in this study.
Authentication	No plants samples were involved in this study.
