# Supplementary material for: Staple oligomers induce a stable RNA G-quadruplex structure for protein translation inhibition in therapeutics
Source: Nat Biomed Eng. 2025 Oct 15;10(6):1124–35. doi: 10.1038/s41551-025-01515-4 (PMC13279272; doi:10.1038/s41551-025-01515-4)
Supplement: Supplementary file 3 — Source data for Figs. 2–4 and Supplementary Figs. 10, 15, 17, 18, 21, 22, 24, 25 and 27. [file 41551_2025_1515_MOESM3_ESM.pdf]

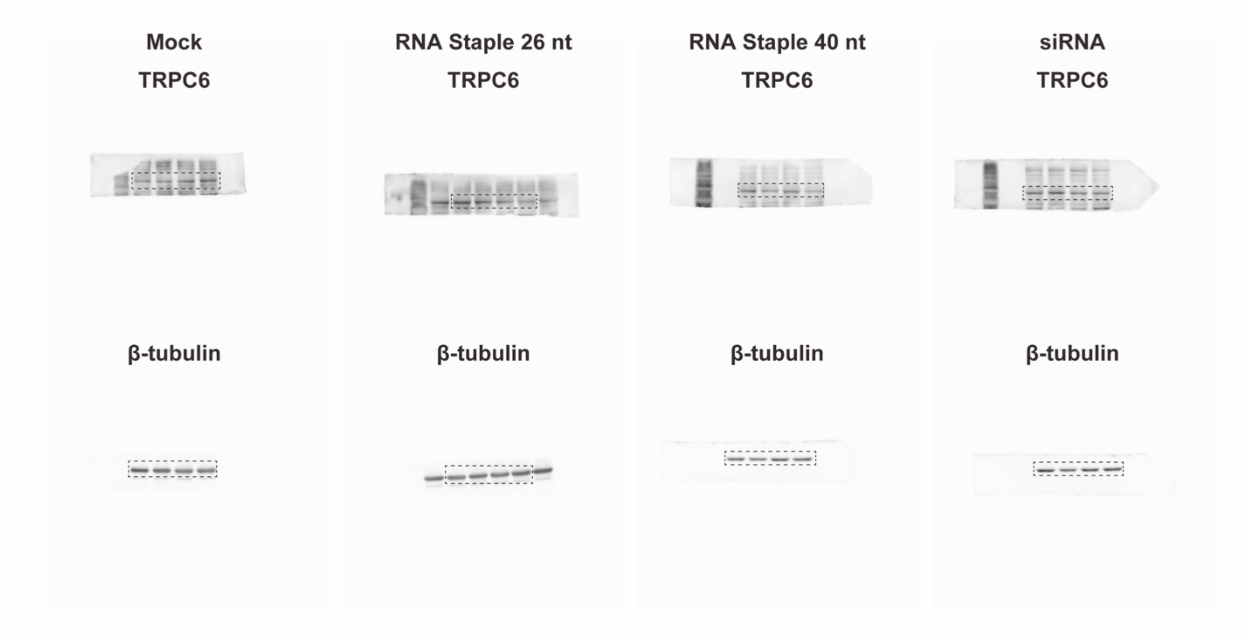

Fig. 2 | In vitro application of RNAh technology to the TRPC6 gene. d, Evaluation of the effects of RNA Staple oligomers and an siRNA on TRPC6 expression in C2C12 cells by western blotting. The gel images within the dash-dotted frame were used as the main figures.

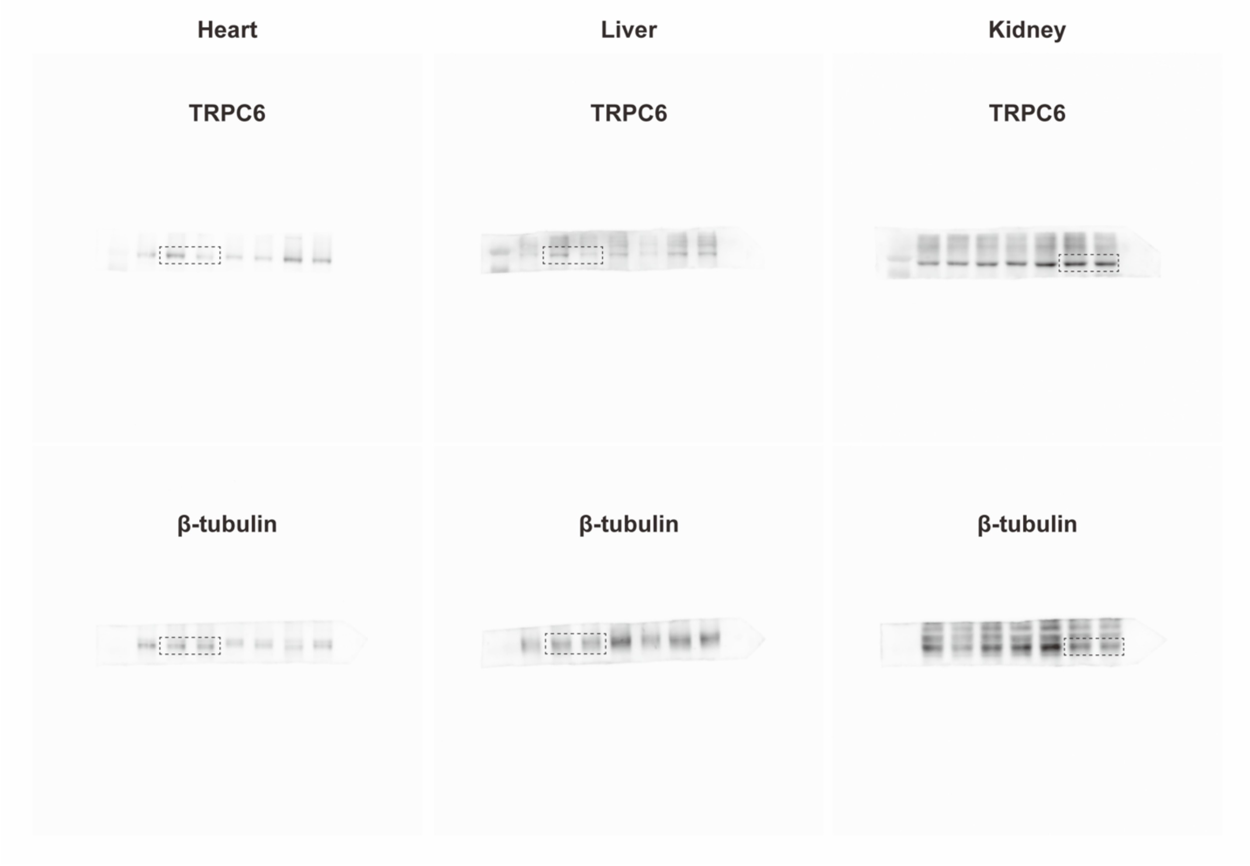

Fig. 3 | Effect of the Staple oligomers on TRPC6 gene expression in mice. Evaluation of protein expression levels of TRPC6 in each organ by western blotting. The gel images within the dash-dotted frame were used as the main figures.

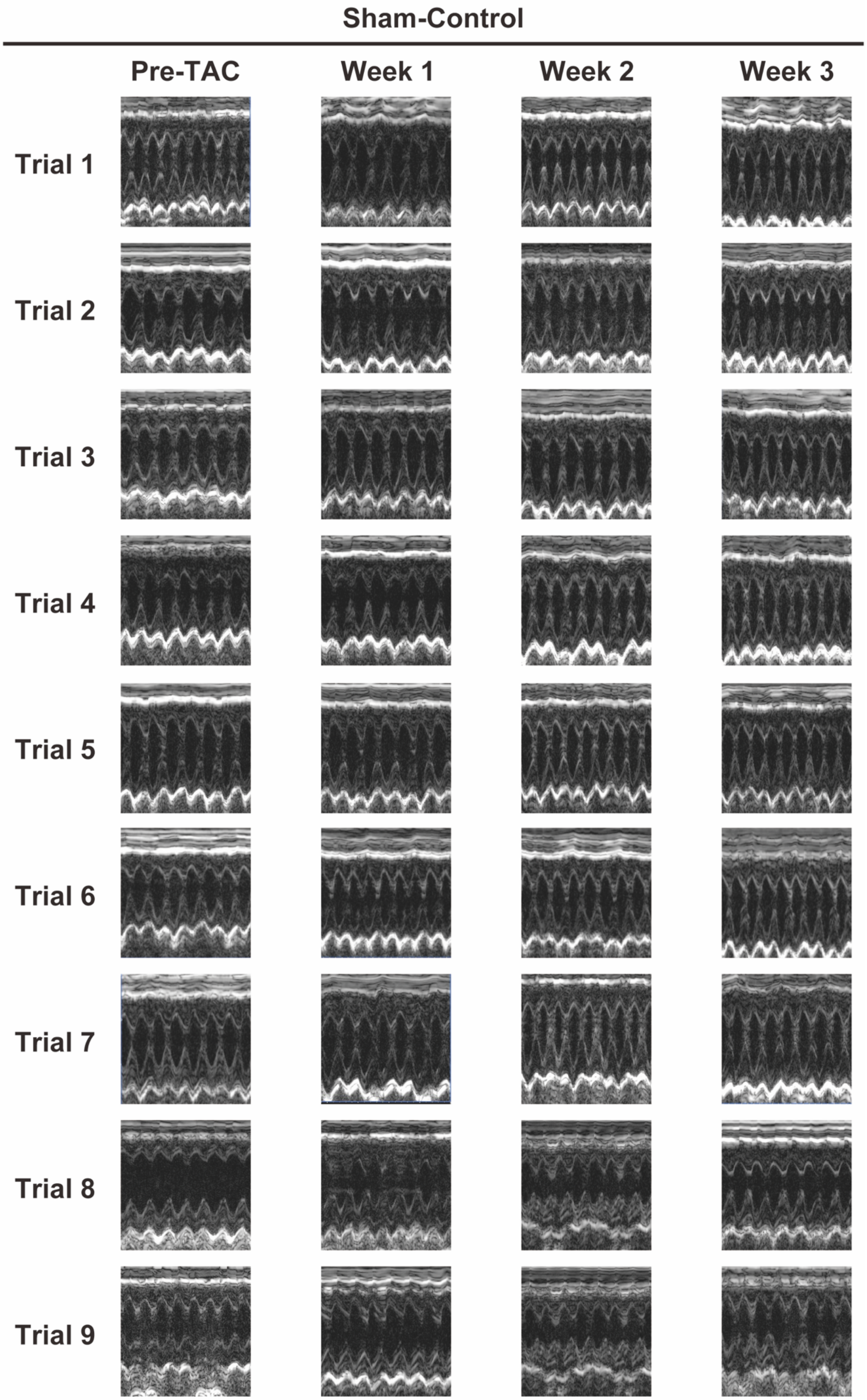
Sham-Control
Pre-TAC
Week 1
Week 2
Week 3
Trial 1
Trial 2
Trial 3
Trial 4
Trial 5
Trial 6
Trial 7
Trial 8
Trial 9

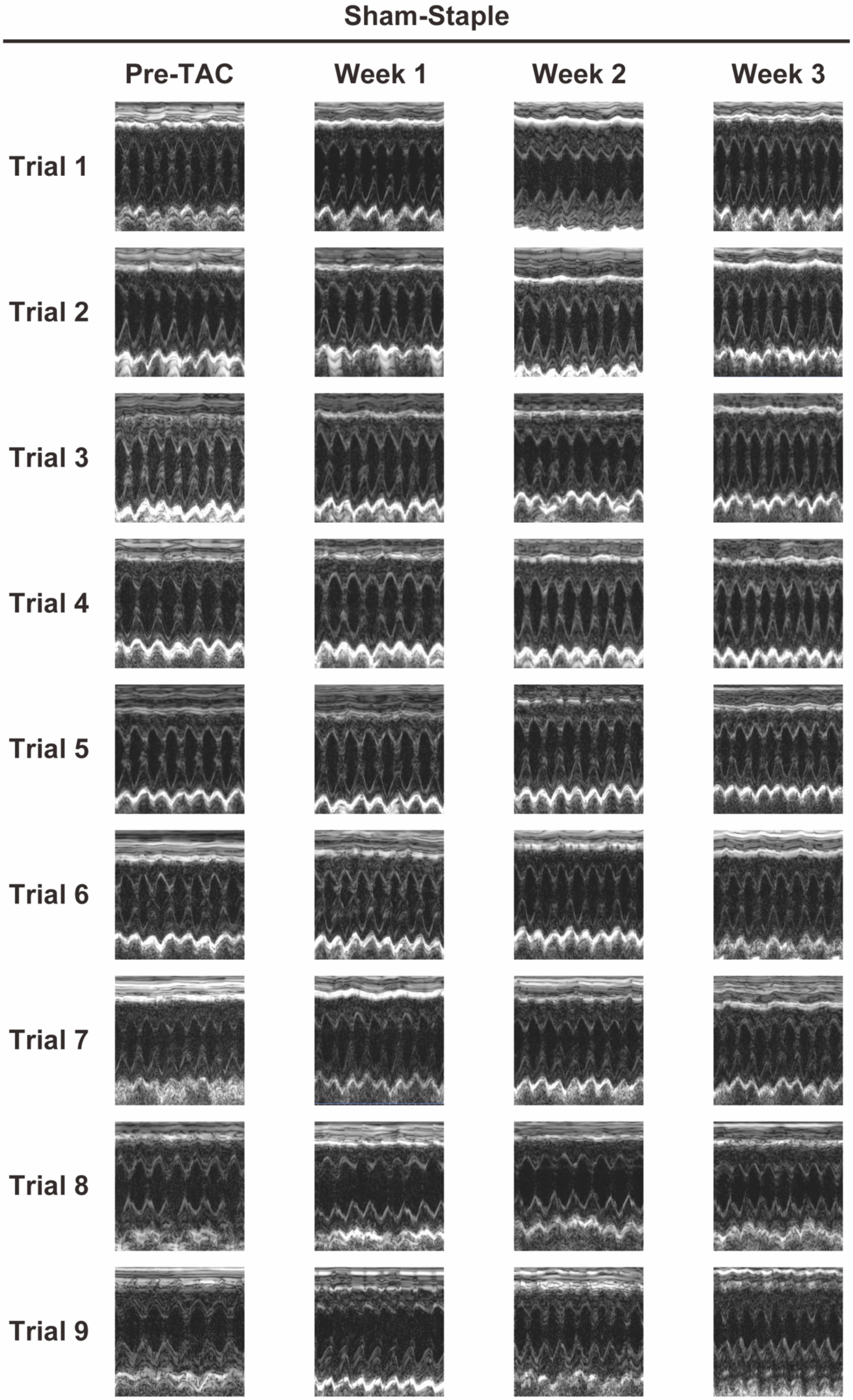
Sham-Staple
Pre-TAC
Week 1
Week 2
Week 3
Trial 1
Trial 2
Trial 3
Trial 4
Trial 5
Trial 6
Trial 7
Trial 8
Trial 9

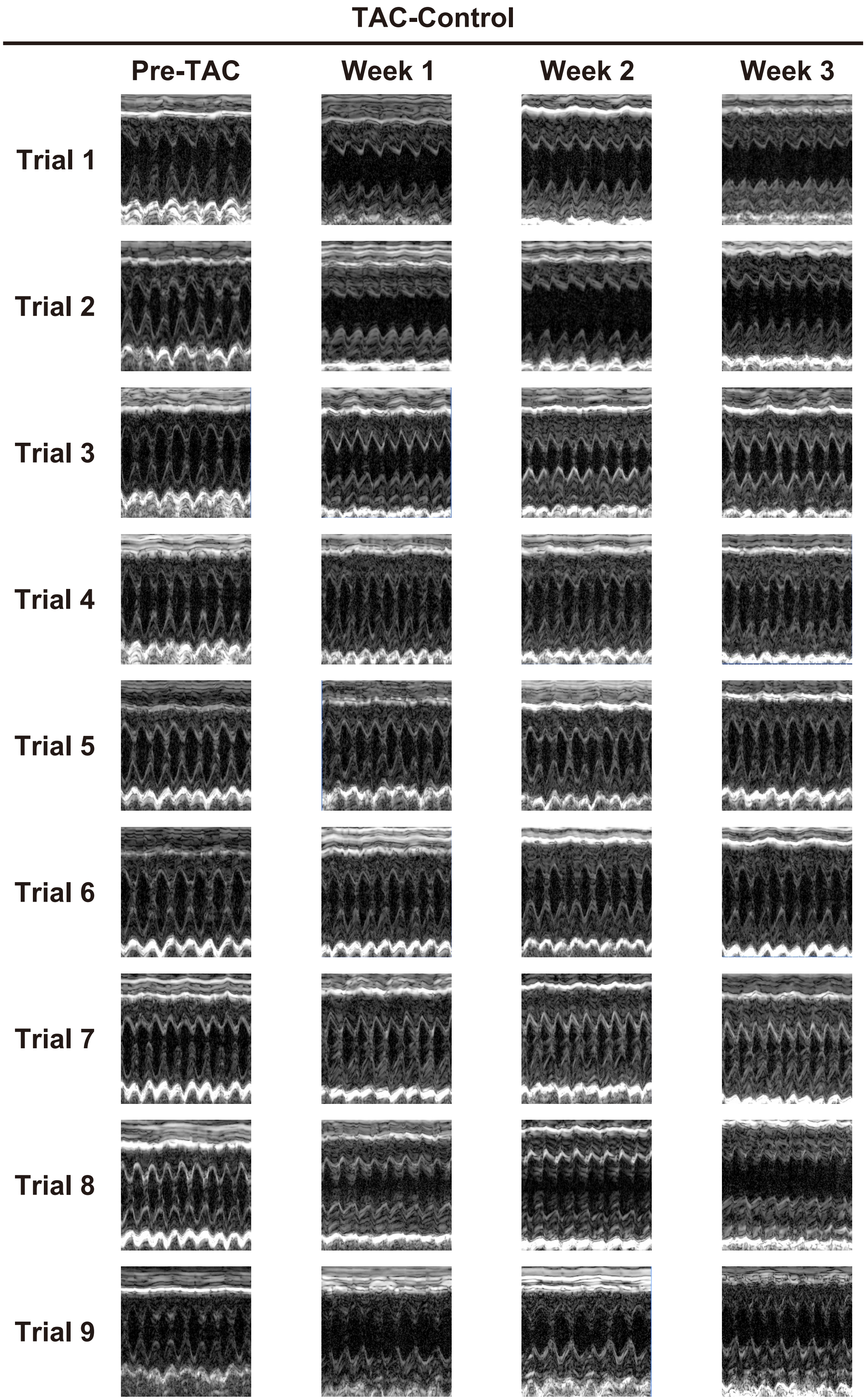
TAC-Control
Pre-TAC
Week 1
Week 2
Week 3
Trial 1
Trial 2
Trial 3
Trial 4
Trial 5
Trial 6
Trial 7
Trial 8
Trial 9

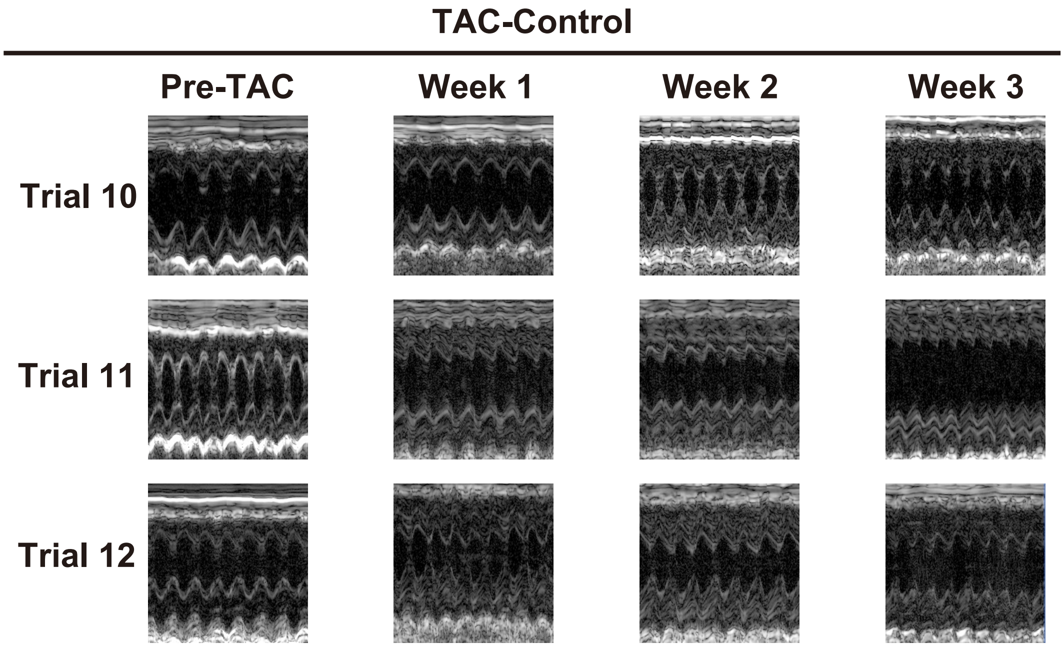

TAC-Control
Pre-TAC
Week 1
Week 2
Week 3
Trial 10
Trial 11
Trial 12

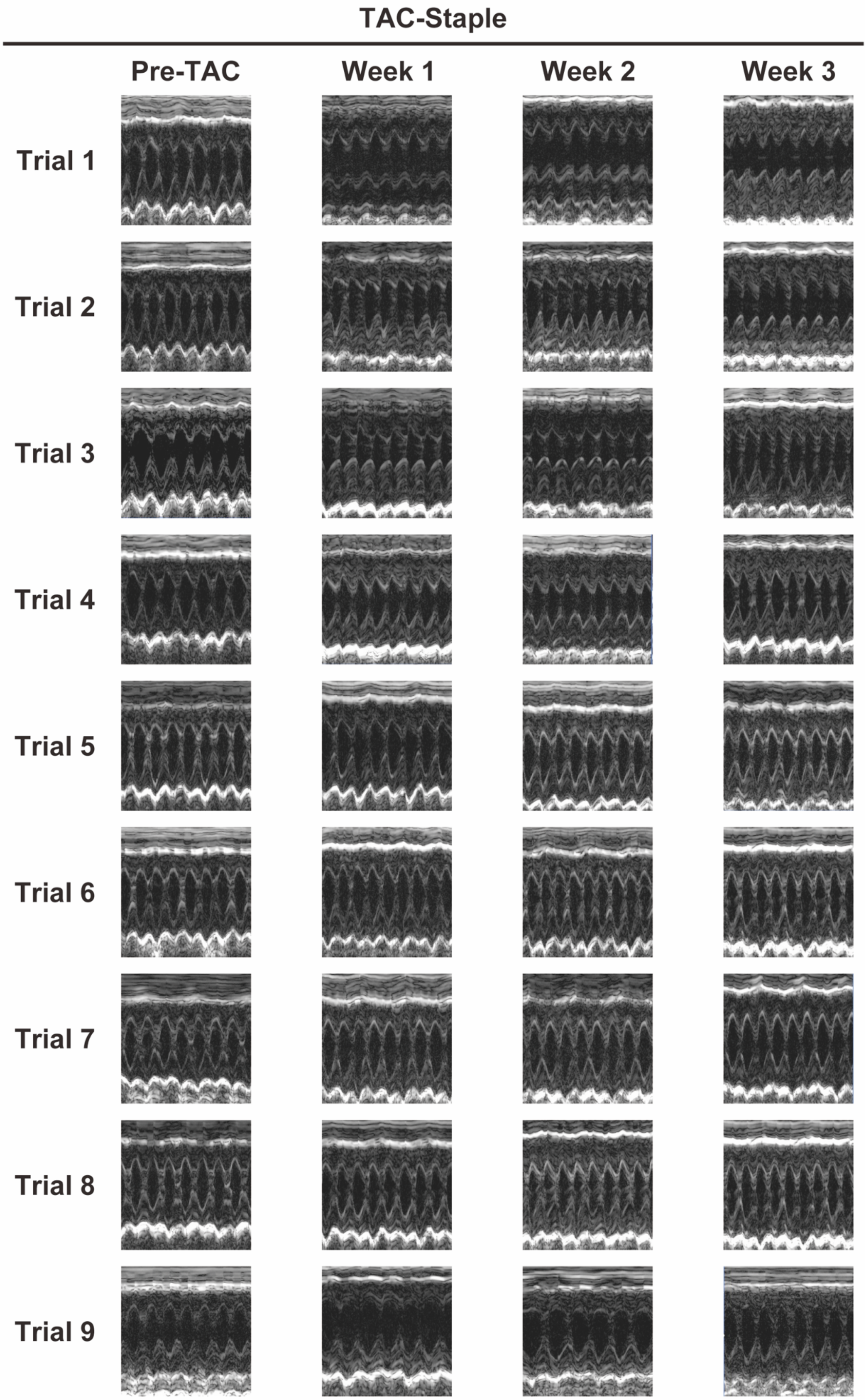
TAC-Staple
Pre-TAC
Week 1
Week 2
Week 3
Trial 1
Trial 2
Trial 3
Trial 4
Trial 5
Trial 6
Trial 7
Trial 8
Trial 9

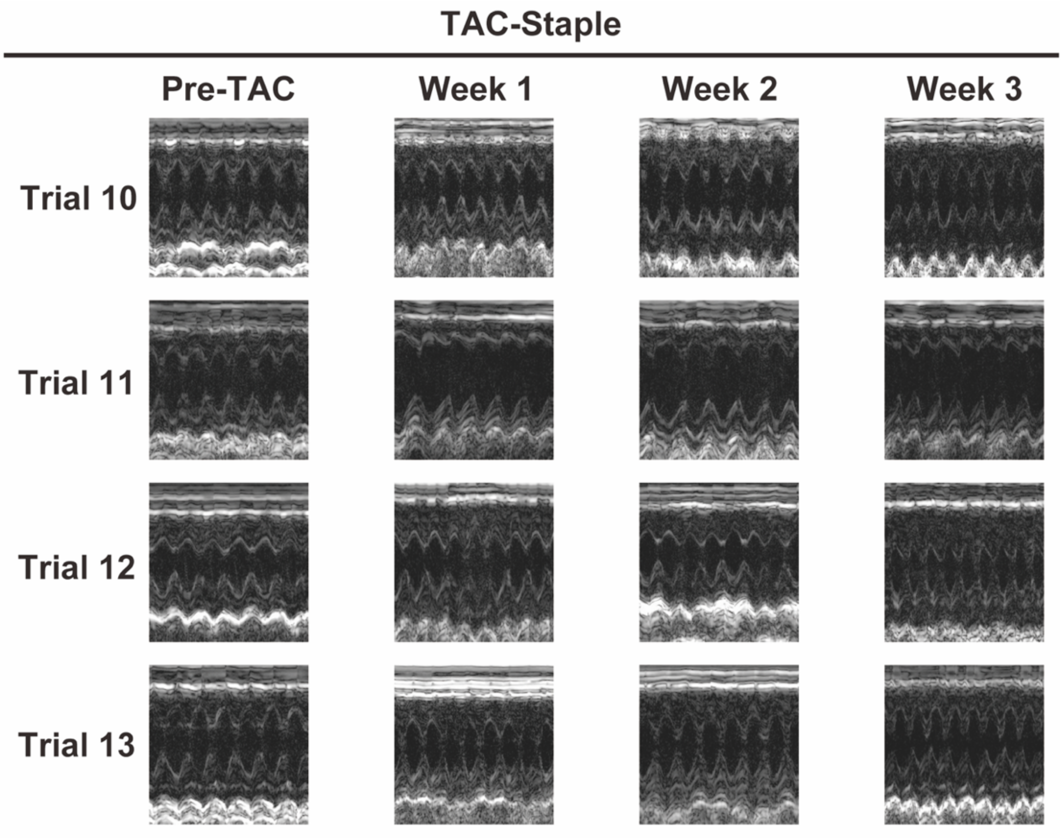

Fig 4 | Effects of Staple oligomers on myocardial hypertrophy by transverse aortic constriction (TAC). c, Echocardiographic evaluation revealed that cardiac function was maintained in the presence of the RNA Staple oligomers.

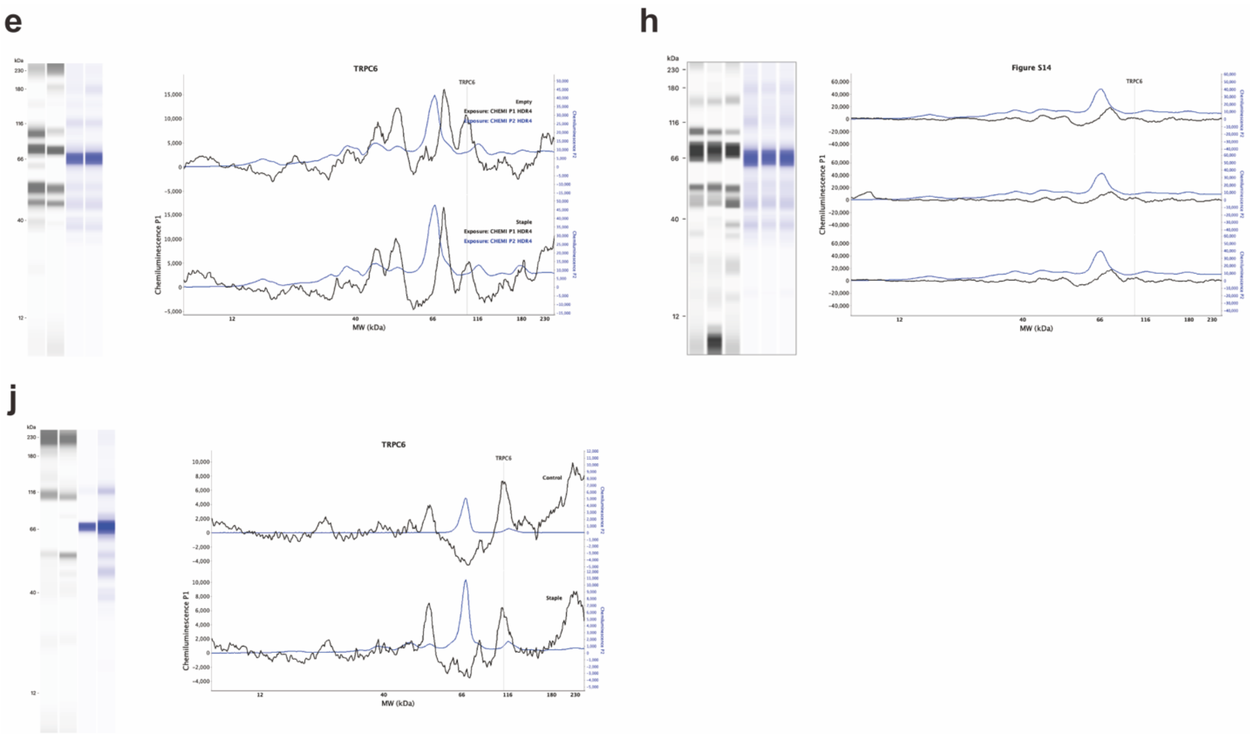

Fig. 4 | Effects of Staple oligomers on myocardial hypertrophy by transverse aortic constriction (TAC). e, h, j, Evaluation of protein expression level of TRPC6 in mouse heart by Abby. TRPC6 expression was analyzed by the band densitometry.

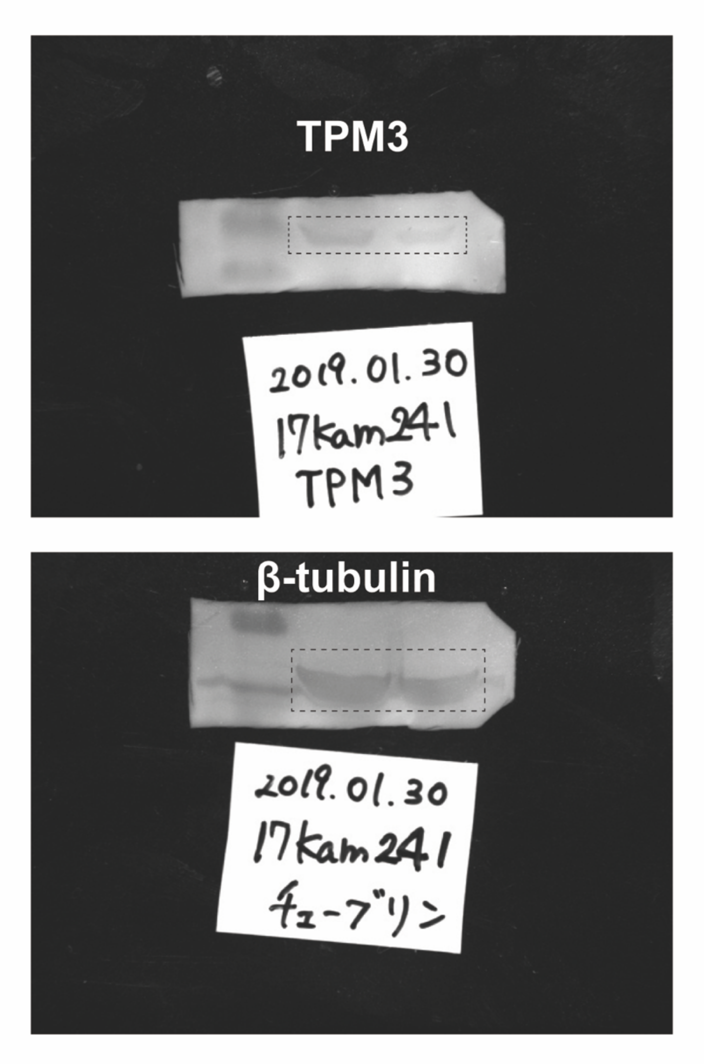

Supplementary Figure 10 | Effect of RNAh technology on target protein expression in mammalian living cells. b, Evaluation of the effect of 26-nt RNA Staple oligomers on TPM3 expression in MCF7 cells by western blotting. The gel images within the dash-dotted frame were used as the supplementary figures.

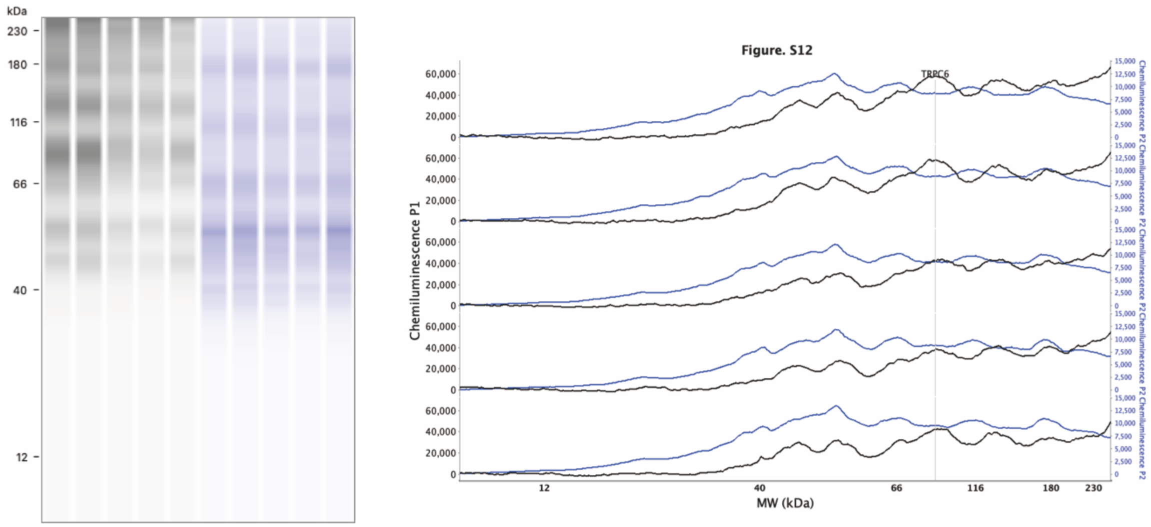

Supplementary Figure 15 | Characterization of RNAh technology with L-*a*TNA-based Staple oligomers. e, Evaluation of the effects of the L-*a*TNA-based Staple oligomers on TRPC6 expression in C2C12 cells by Abby. TRPC6 expression was analyzed by the band densitometry.

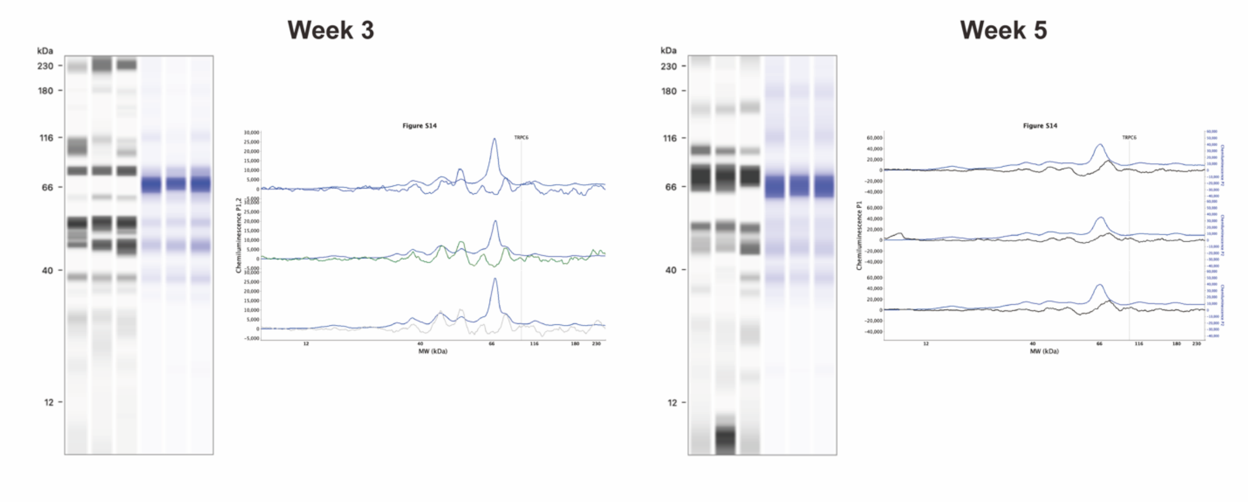

Supplementary Figure 17 | Effects of L-*a*TNA-based Staple oligomers on TRPC6 expression in mouse hearts. Western blotting analysis by Abby revealed that mice treated with L-*a*TNA-based Staple oligomers showed reduced expression of TRPC6 in a dose-dependent manner at weeks 3 and weeks 5 after treatment. TRPC6 expression was analyzed by the band densitometry.

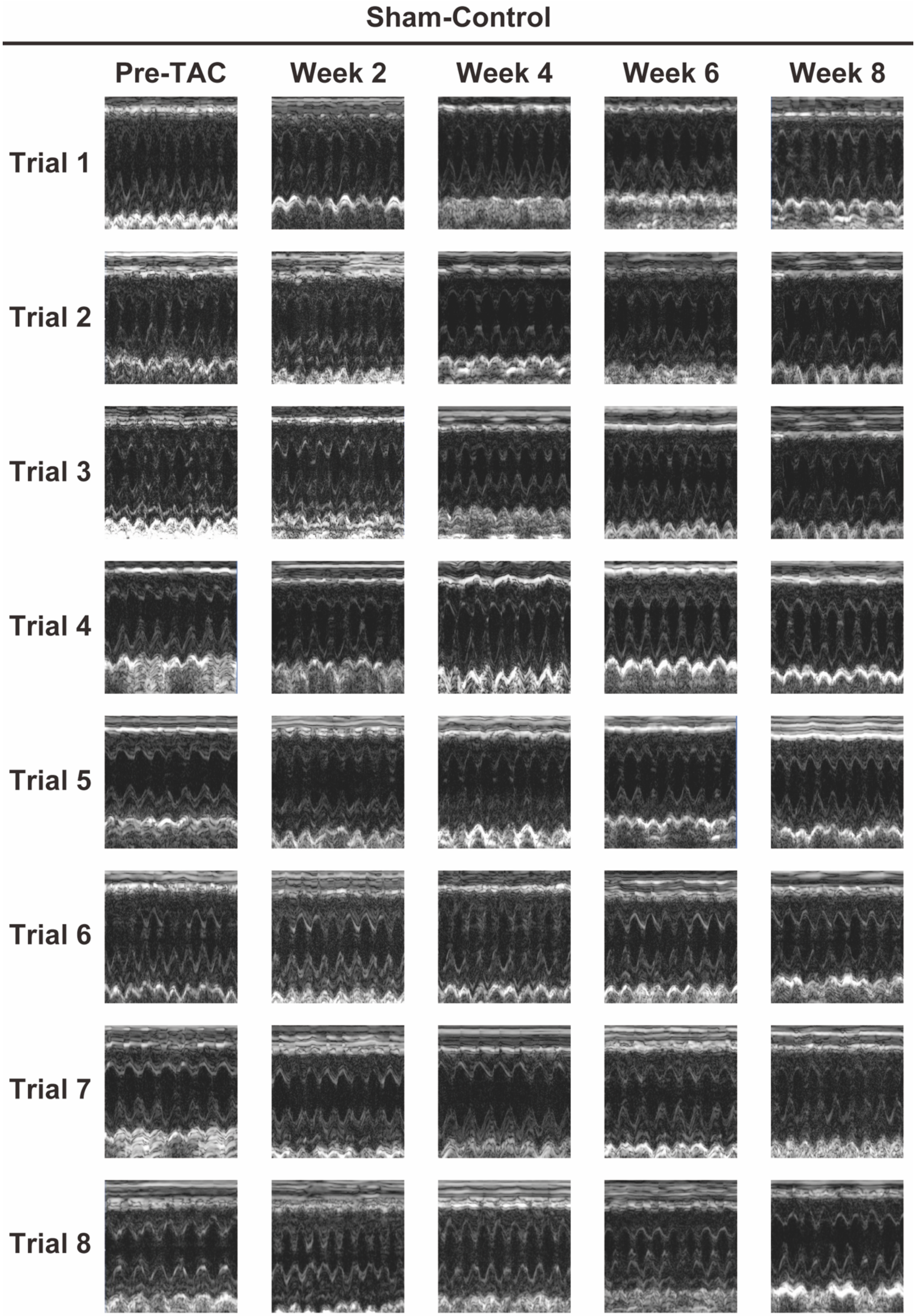
Sham-Control
Pre-TAC
Week 2
Week 4
Week 6
Week 8
Trial 1
Trial 2
Trial 3
Trial 4
Trial 5
Trial 6
Trial 7
Trial 8

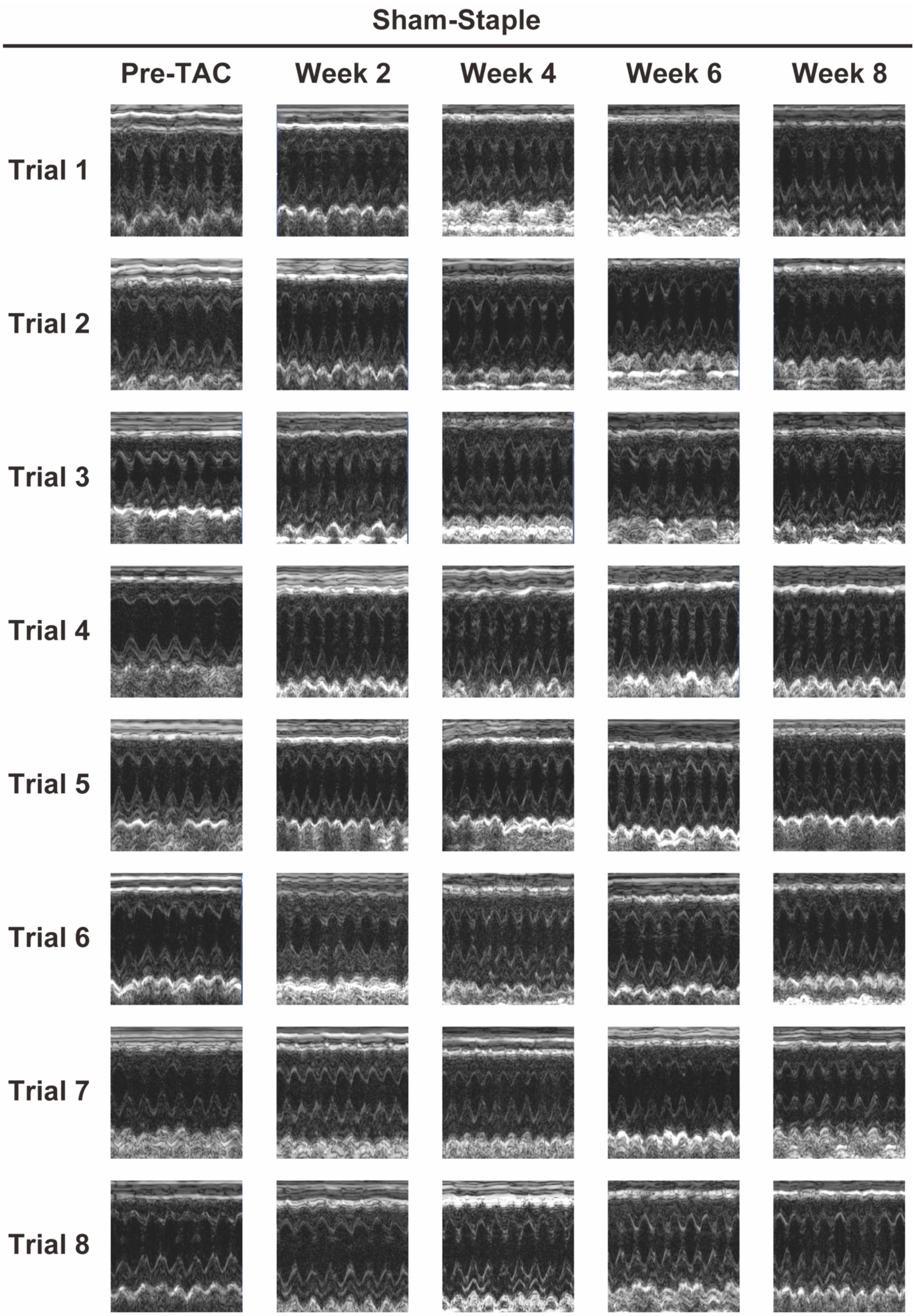
Sham-Staple
Pre-TAC
Week 2
Week 4
Week 6
Week 8
Trial 1
Trial 2
Trial 3
Trial 4
Trial 5
Trial 6
Trial 7
Trial 8

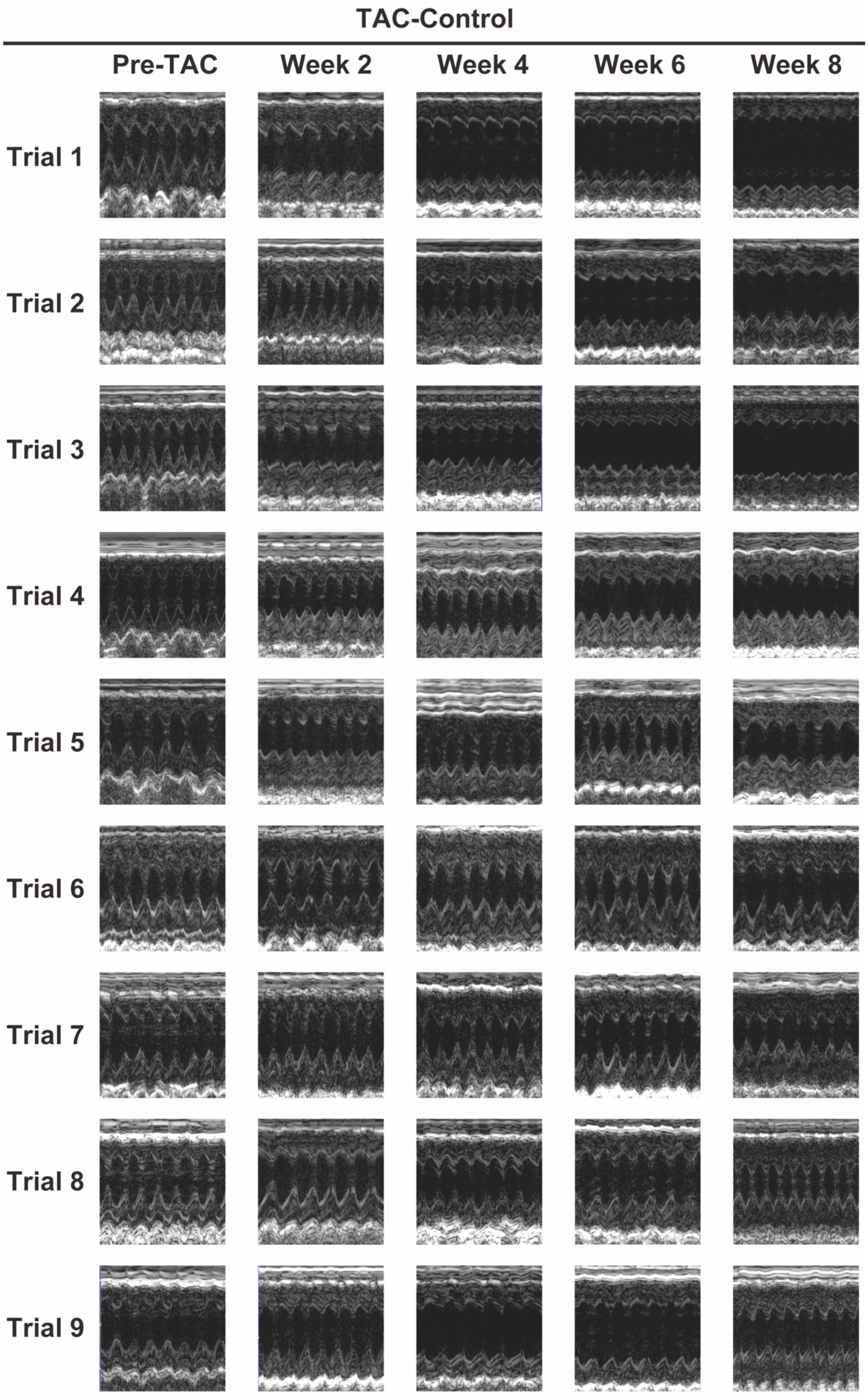

TAC-Control
Pre-TAC
Week 2
Week 4
Week 6
Week 8
Trial 1
Trial 2
Trial 3
Trial 4
Trial 5
Trial 6
Trial 7
Trial 8
Trial 9

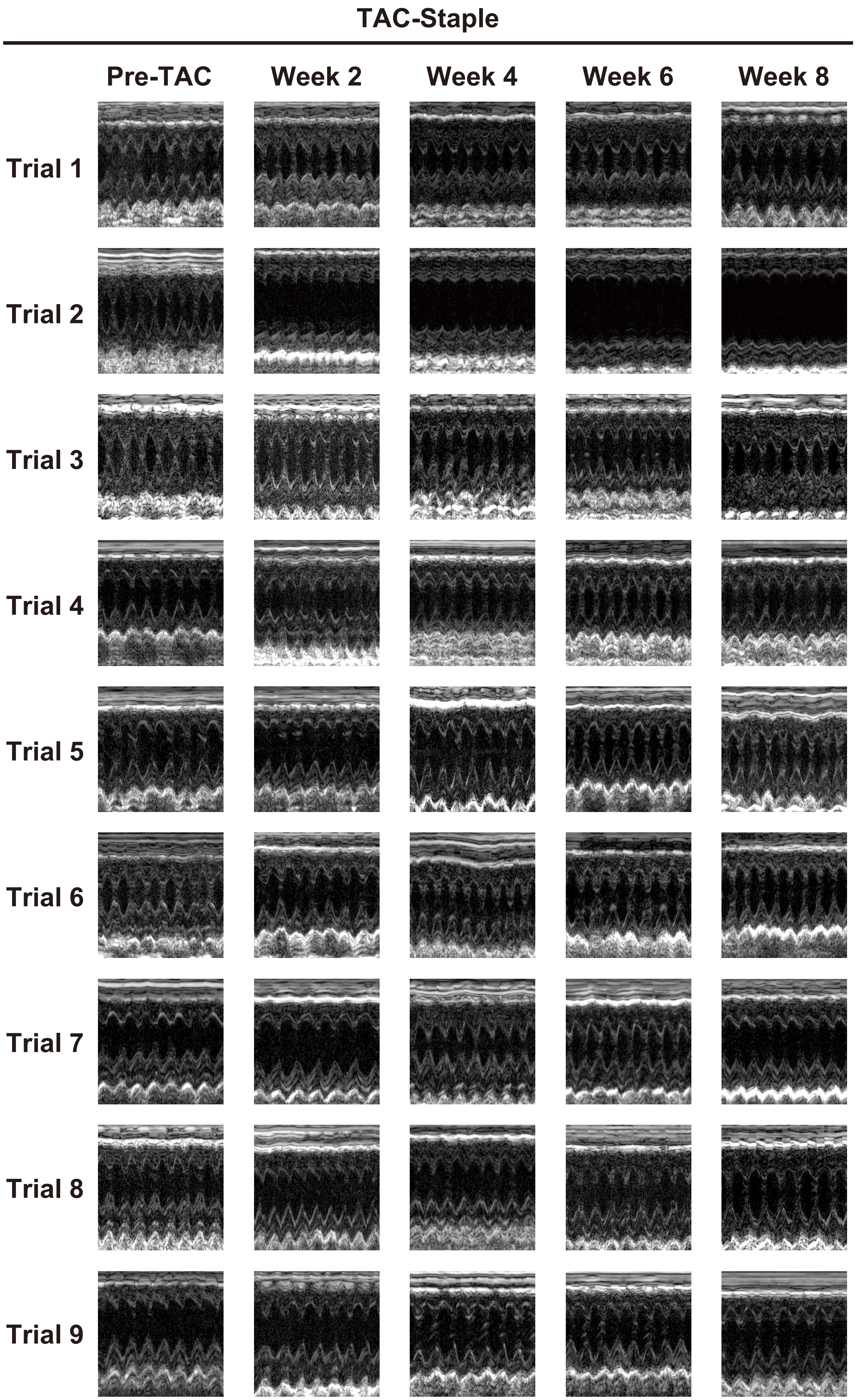
TAC-Staple
Pre-TAC
Week 2
Week 4
Week 6
Week 8
Trial 1
Trial 2
Trial 3
Trial 4
Trial 5
Trial 6
Trial 7
Trial 8
Trial 9

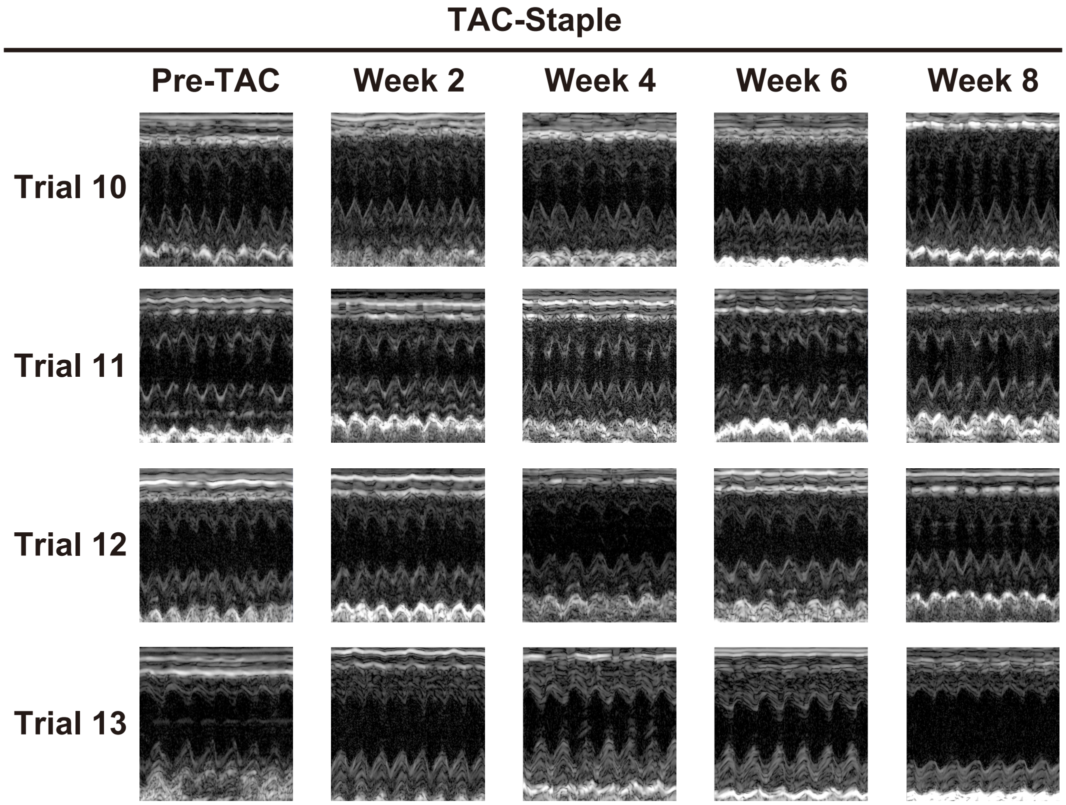

Supplementary Figure 18 | Effects of L-*a*TNA-based Staple oligomer on cardiac function in TAC-treated mouse hearts. b, Echocardiography showed no significant decrease in cardiac function after TAC treatment in the 2.0 mg/kg 40-nt L-*a*TNA Staple oligomer-treated mice, but a marked decrease in the non-Staple-oligomer-treated mice up to weeks 8.

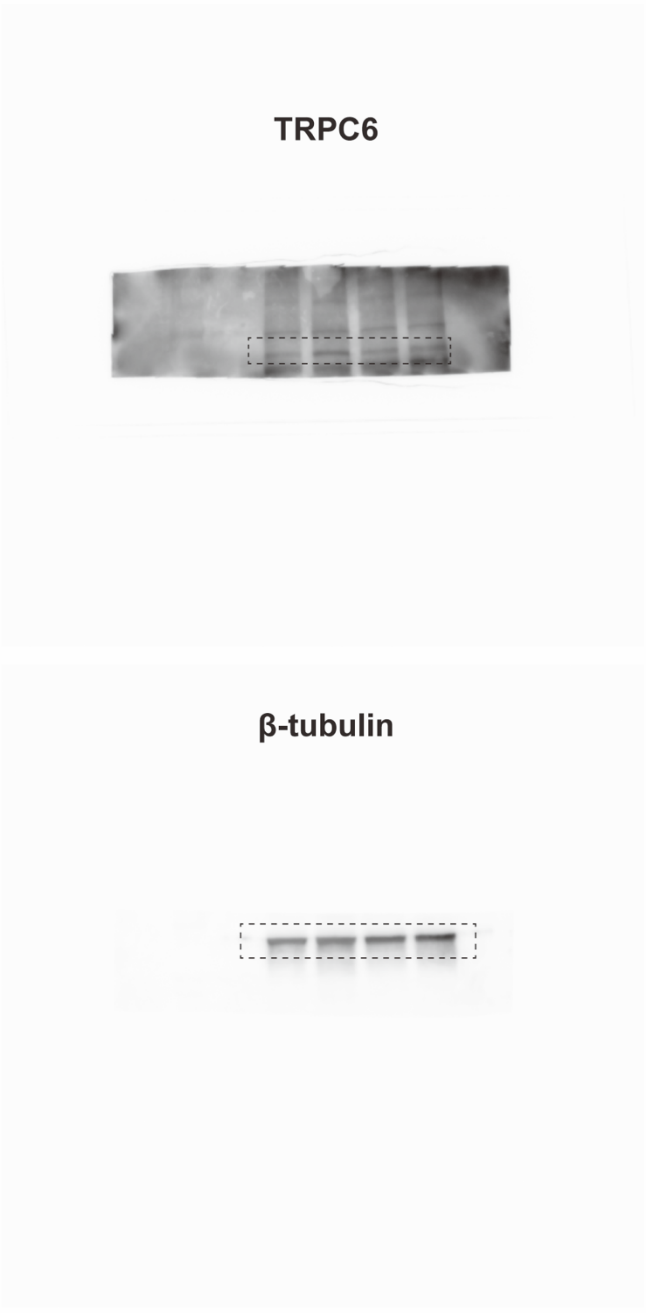

Supplementary Figure 21 | Effect of siRNA on TRPC6 protein expression in NIH3T3 cells. The gel images within the dash-dotted frame were used as the main figures.

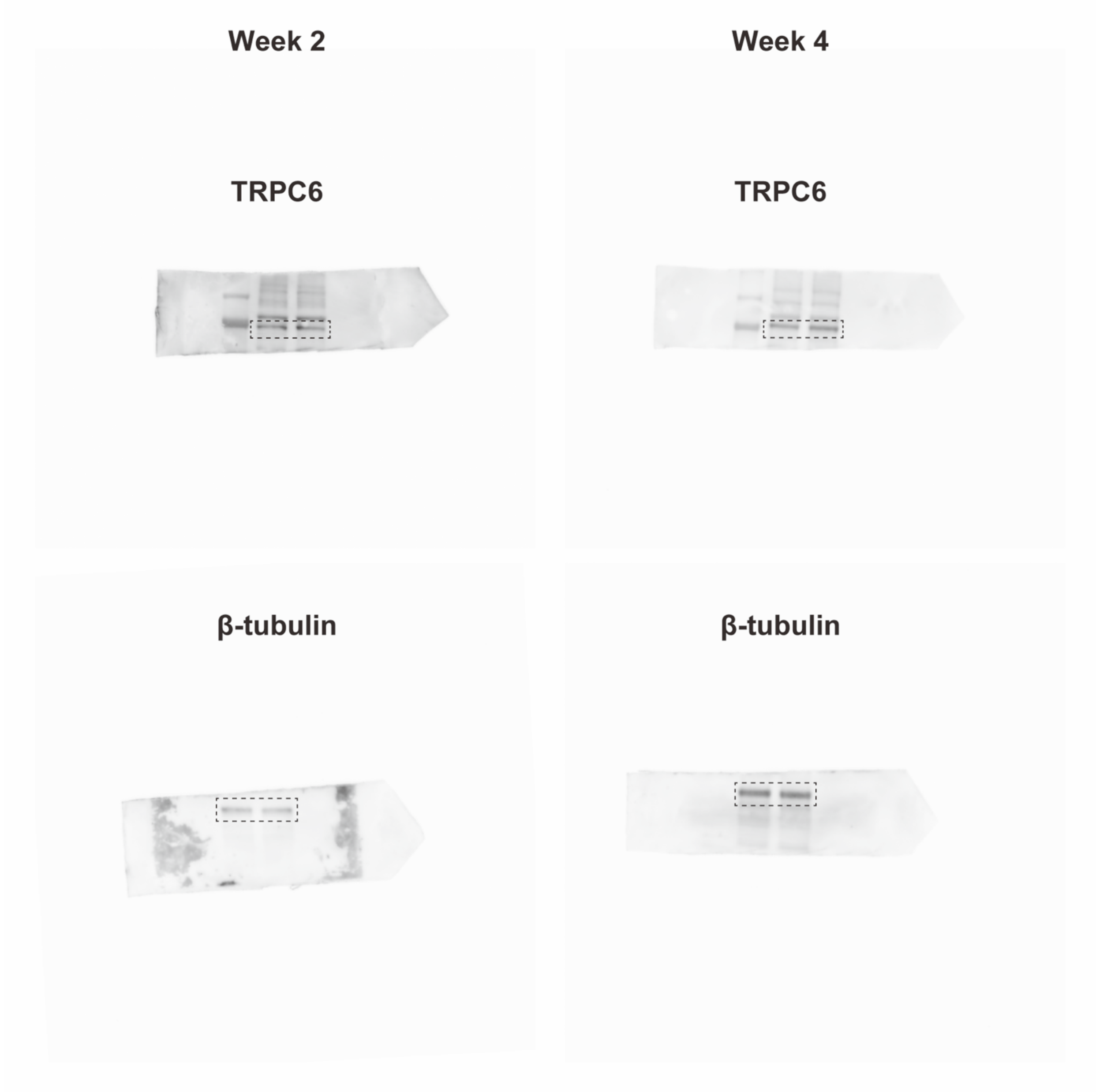

Supplementary Figure 22 | Effects of siRNA on TRPC6 expression in mouse hearts. The gel images within the dash-dotted frame were used as the main figures.

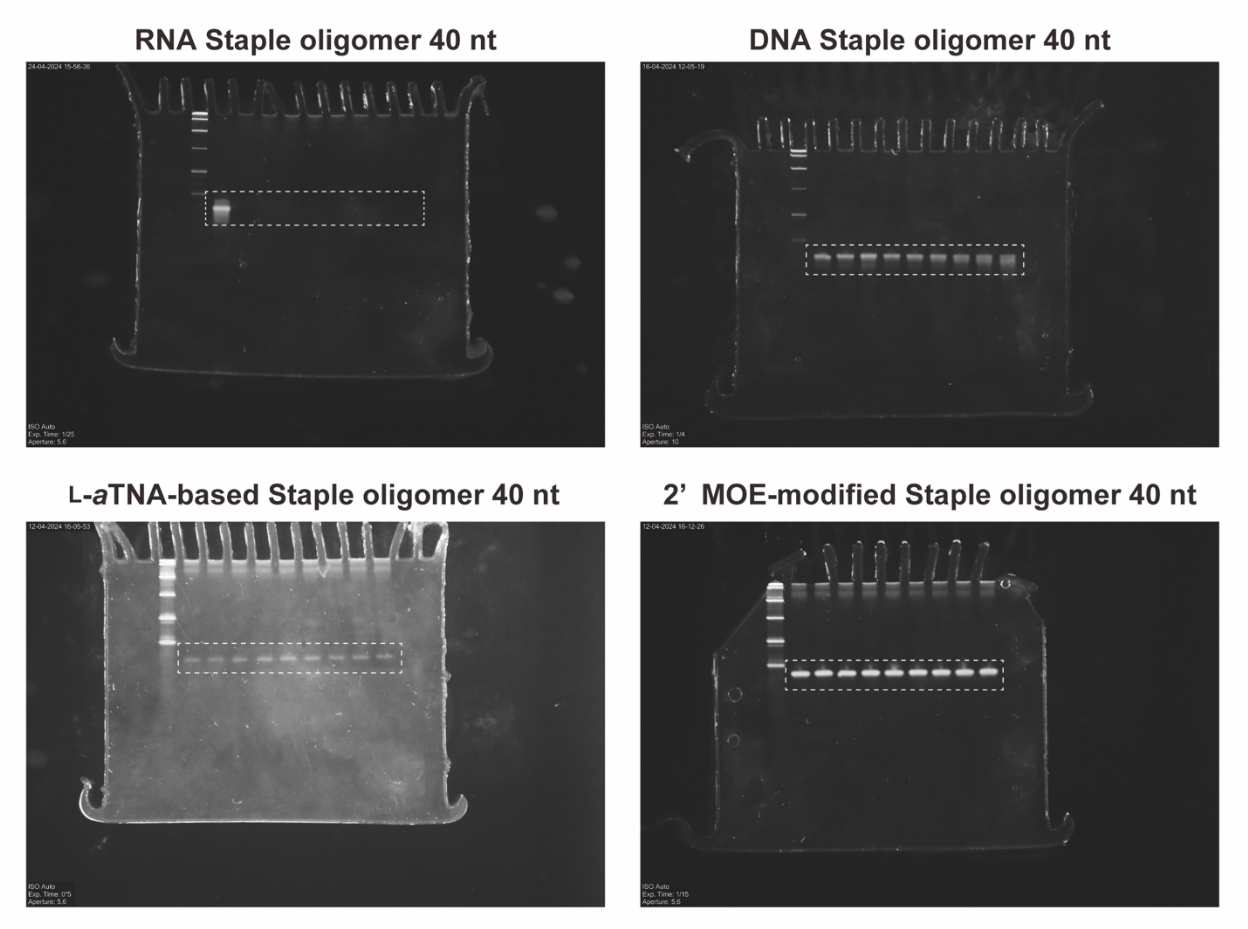

Supplementary Figure 24 | Evaluation of biological stability of various Staple oligomer under 10% FBS conditions by denaturing PAGE. The gel images within the dash-dotted frame were used as the main figures.

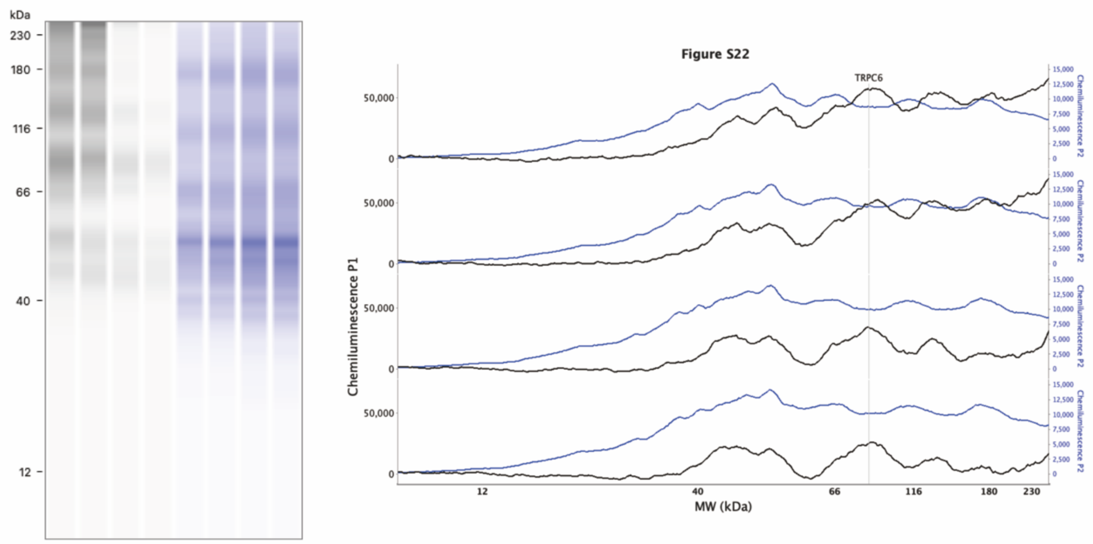

Supplementary Figure 25 | Characterization of RNAh technology with 2′MOE-modified Staple oligomer. d, Evaluation of the effects of the 2′MOE-modified Staple oligomers on TRPC6 expression in C2C12 cells by Abby. TRPC6 expression was analyzed by the band densitometry.

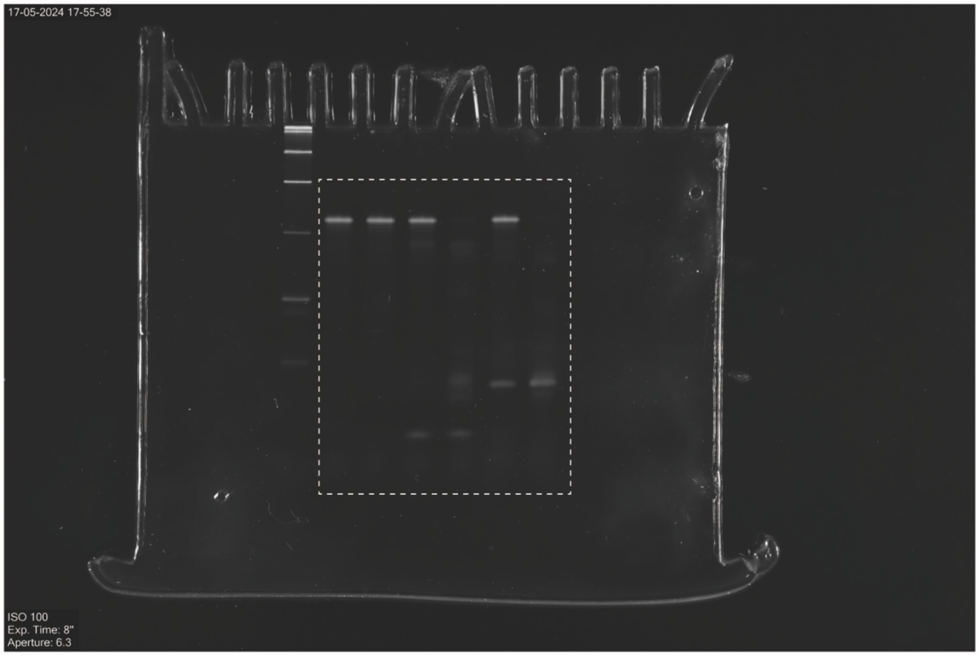

Supplementary Figure 27 | Validation of the cooperation of RNAh technology with RNaseH. DNA Staple oligomers activates RNaseH-mediated cleavage of target RNA. The residual target RNA after RNaseH treatment with DNA Staple oligomers was characterize by denaturing PAGE. The gel images within the dash-dotted frame were used as the main figures.
